# Primary Cilium Forces Neuroendocrine Shift in Prostate Cancer through YAP1 Repression and Reduced Mitochondrial Activity

**DOI:** 10.7150/thno.126688

**Published:** 2026-05-18

**Authors:** Yingbo Guo, Siyong Peng, Thibaud Jamet, Virginie Firlej, Marie Irondelle, Constance Nau, Christophe A. Girard, Kaushal Asrani, Tamara L. Lotan, Romain Huc, Pascale Soyeux, Matthieu Rouleau, Sandra Lacas-Gervais, Amandine Rovini, Samantha Luciano, Olivier Humbert, Renaud Schiappa, Marc Pujalte-Martin, Mathieu Vigneau, Pascal Peraldi, Frédéric Bost, Gwendal Lazennec, Francis Vacherot, Nathalie M. Mazure

**Affiliations:** 1 Université Côte d’Azur, INSERM U1065, C3M, 151 Route de St Antoine de Ginestière, BP2 3194, CEDEX 03, 06204 Nice, France – Equipe 5.; 2 Equipe labellisée Ligue contre le Cancer.; 3 Univ Paris Est Creteil, TRePCa, F-94010 Creteil, France.; 4 Université Paris-Est Créteil, Inserm U955, Institut Mondor de recherche biomédicale (IMRB), Créteil, France.; 5 Université Côte d’Azur, INSERM U1065, C3M, 151 Route de St Antoine de Ginestière, BP2 3194, CEDEX 03, 06204 Nice, France – Equipe 11.; 6 Department of Pathology, Johns Hopkins University School of Medicine, Baltimore, MD, USA.; 7 Université Côte d'Azur, CNRS, LP2M, Nice, France.; 8 Université Côte d'Azur, Centre Commun de Microscopie Appliquée, CCMA, Nice, France.; 9 Université de Limoges, INSERM UMR 1308, CAPTuR, Limoges, France.; 10 Centre Antoine Lacassagne, Biological Resource Center, 33 Av. de Valombrose, 06100 Nice, France.; 11 Centre Antoine Lacassagne, Department of Nuclear Medicine, 33 Av. de Valombrose, 06100 Nice, France.; 12 Centre Antoine Lacassagne, Department of Epidemiology, Biostatistics and Health Data, 33 Av. de Valombrose, 06100 Nice, France.France.; 13 University of Toulouse, CNRS UMR 5070, INSERM U1301, EFS, ENVT, Institut RESTORE,Toulouse, France.; 14 CNRS, UMR9005, SYS2DIAG, Cap delta, 1682 rue de la Valsière, Montpellier, France.

**Keywords:** glycolysis, hypoxia, mitochondria, neuroendocrine transdifferentiation, primary cilium, prostate cancer, YAP1

## Abstract

Primary cilia are increasingly recognized as regulators of cellular signaling and plasticity. Here, we examined their distribution and potential relevance in neuroendocrine (NE) prostate cancer. While typically absent in localized hormone-sensitive prostate tumor cells, we detected primary cilia in neuroendocrine-like cells both *in vitro* and in castration-resistant prostate cancer (CRPC) samples. *In vivo*, cilia were consistently observed in CRPC tumor cells exhibiting FDG-PET positivity and NE features, supporting an association between ciliogenesis, metabolic reprogramming, and disease progression. These aggressive tumors also displayed reduced mitochondrial activity, consistent with a shift away from oxidative metabolism. Building on our work in ccRCC, we identified a GLI1⁺/IFT20⁺ or GLI1⁺/IFT80⁺ signature enriched in ciliated, NE-prone subpopulations. *In vitro*, YAP1 inhibition alone did not induce ciliogenesis, whereas cytoskeletal remodeling with jasplakinolide restored cilium assembly and enabled partial NE transdifferentiation. Single-cell RNA-seq analyses further showed enrichment of ciliogenesis-related genes within NE clusters in CRPC. Together, these observations support a model in which primary cilia are closely associated with NE identity and metabolic adaptation, rather than serving solely as passive markers, and suggest a structural–metabolic axis that may represent a source of biomarkers and therapeutic vulnerabilities.

## Introduction

Prostate cancer (PCa) ranks as the second most diagnosed cancer and the fifth leading cause of cancer-related deaths among men worldwide. In 2020, there were approximately 1.4 million new cases of prostate cancer diagnosed globally, with around 375,000 deaths attributed to the disease 1-3. PCa primarily affects older men, with most cases diagnosed in men over the age of 50. Survival rates are generally high, particularly when diagnosed at an early stage, but decrease significantly for advanced or metastatic disease. Treatment options range from active surveillance to surgery, radiation therapy, hormone therapy, chemotherapy, and targeted therapy, with ongoing research aimed at improving early detection, diagnosis, and treatment outcomes. Castration-resistant prostate cancer (CRPC) often emerges as a consequence of androgen deprivation therapy (ADT), a standard treatment for advanced prostate cancer 4-6. Prolonged ADT reduces androgen levels, thereby inhibiting the growth and proliferation of androgen-sensitive PCa cells. However, despite initial response, many patients eventually develop CRPC, characterized by continued tumor growth and progression despite castrate levels of androgens. ADT can promote the transition from an epithelial-like to a neuroendocrine (NE)-like phenotype, a process known as Neuroendocrine Transdifferentiation (NED) 7-9. This adaptive response to androgen deprivation involves cellular stress, activation of specific molecular pathways, and may also favor the survival and expansion of NE cell populations within the tumor. The differentiation of adenocarcinoma to neuroendocrine prostate tumors occurs in 15 to 20% of patients with castration-resistant prostate cancer (CRPC). In these tumors, NE cells coexist with adenocarcinoma cells, and their presence is associated with aggressive disease behavior, resistance to conventional therapies, and poor clinical outcomes 10, 11. NED in prostate cancer is driven by key molecular alterations, including activation of the PI3K/Akt/mTOR pathway and disruption of the TP53 and RB1 tumor suppressors, which impair cell cycle control and promote NE features. Transcription factors such as ASCL1, FOXA2, and EZH2 further regulate NE marker expression, while crosstalk with androgen receptor signaling amplifies this process. In parallel, NE tumors exhibit remarkable metabolic diversity, reflecting variations in lineage origin, transcriptional programs, and adaptation to the tumor microenvironment. Recent evidence supports a context-dependent view in which mitochondrial function during NE differentiation can be maintained or even enhanced, depending on lineage and experimental conditions. In small-cell lung cancer (SCLC), Peinado *et al.* reported that electrically active ASCL1-positive NE cells exhibit high ATP demand and rely heavily on oxidative phosphorylation (OXPHOS) to sustain their secretory phenotype 12. Consistently, Solta *et al.* showed that ASCL1-driven SCLC subtypes are enriched in mitochondrial mass and respiratory chain activity, underscoring a metabolic heterogeneity across NE lineages 13. In prostate cancer, Crowell *et al.* demonstrated that androgen receptor (AR) inhibition triggers a metabolic rewiring characterized by increased mitochondrial respiration and DRP1-dependent remodeling, suggesting that mitochondrial plasticity contributes to therapy-induced NE features 14. Along the same lines, data from Zhang *et al.* revealed that PGC1α activation enhances oxidative metabolism and supports survival of NE-like cells 15. However, other studies indicate that specific NE states can also adopt a more glycolytic phenotype, with enhanced glucose utilization and metabolic reprogramming toward glycolysis 16-18, further illustrating the metabolic plasticity of NE tumors. Together, these studies indicate that NE tumors can retain, or even enhance, mitochondrial oxidative capacity, highlighting the complexity of metabolic regulation during NE differentiation. Identifying additional drivers of NED remains essential for the development of targeted therapies in advanced prostate cancer.

In the normal prostate epithelium, primary cilia (PC) are readily detectable and are thought to participate in epithelial differentiation and signaling homeostasis 19. In contrast, multiple studies have reported a marked loss or shortening of primary cilia in prostate adenocarcinoma, consistent with increased proliferation and cell cycle dysregulation 20, 21. Loss of the primary cilium has been described as an early event during prostate tumorigenesis and is associated with androgen receptor–driven proliferation and suppression of ciliogenesis-related pathways. However, the status of the primary cilium in advanced prostate cancer subtypes, particularly neuroendocrine prostate cancer (NEPC), remains poorly defined, largely due to limited access to patient material and the rarity of this aggressive entity 22. In parallel, growing evidence across multiple cancer types has revealed that the primary cilium is not merely lost during tumorigenesis but can be dynamically regulated and, in some contexts, re-expressed, where it actively contributes to signaling rewiring, metabolic adaptation and therapy resistance 23, 24.

The emerging role of the PC in cancer research has drawn significant attention in recent years, shedding light on its multifaceted involvement in tumorigenesis and tumor progression. Previously dismissed as vestigial, the PC is now acknowledged as pivotal in cellular functions like signaling, proliferation, and differentiation 20. Dysregulation of PC dynamics in cancer has been linked to tumor growth, metastasis, and therapy resistance. It serves as a signaling hub, governing pathways such as Hedgehog, Wnt, and mTOR, often dysregulated in cancer. Additionally, dysfunction of the PC correlates with hallmark features of cancer cells, such as genomic instability and aberrant cell cycle progression. Notably, the primary cilium's presence is inversely associated with cell proliferation 25, typically retracting before mitosis through resorption 26, involving processes like intraflagellar transport (IFT) regulation and microtubule de-acetylation 27. Within the PC, key Hedgehog pathway components, like Patched-1 (Ptch1) and Smoothened (Smo), are finely regulated. Activation of this pathway leads to nuclear translocation of GLI proteins, orchestrating transcriptional regulation vital for cellular processes including proliferation and survival 28. The PC thus serves as a critical platform for Hedgehog pathway modulation, and its dysregulation can contribute to cancer initiation and progression. Our previous investigation explored the regulation of ciliogenesis in clear cell Renal Cell Carcinoma (ccRCC), examining both renal cancer cells and patient samples 23. We identified a subgroup of ccRCC patients (*GLI1^+^*/*IFT20^+^*) characterized by PC re-expression, enhanced glycolysis, and features of epithelial-mesenchymal transition, consistent with aggressive tumor behavior irrespective of VHL status 23. These patients showed reduced sensitivity to sunitinib but potential responsiveness to immunotherapy, highlighting new therapeutic avenues that may extend to other cancers such as PCa, where the PC is absent.

This prompted us to draw a parallel with prostate cancer, where Pearson *et al.* demonstrated that the Yes-associated protein (YAP) status similarly stratifies tumor into two distinct molecular categories 29. They demonstrated that contrasting pro- or anti-cancer activities of YAP functionally categorize cancers into binary YAP^on^ or YAP^off^ classes, depending on whether they express or silence YAP, respectively. YAP^off^ solid cancers are predominantly neural/neuroendocrine and frequently associated with *RB1* gene mutations, such as retinoblastoma, small cell lung cancer, and NE prostate cancer. YAP1 silencing may either be intrinsic to the cell's origin or acquired through lineage switching and drug resistance. These binary cancer groups exhibit different adhesive behaviors dependent on YAP1 and have distinct vulnerabilities to pharmaceutical interventions, highlighting their clinical significance. Emerging evidence suggests that YAP activity may also influence primary cilium dynamics and function 29-33. Conversely, the PC may also modulate YAP1 signaling, potentially through interactions with mechanosensitive pathways or other signaling cascades.

In this study, we investigated the potential for PC re-expression in NE prostate cancer, an extremely aggressive and highly treatment-resistance subtype where access to patient tissue is even more limited. Using restricted patient-derived samples together with *in vitro* models of tumor progression, we assessed PC status with the GLI1+/IFT20+ signature and examined its interplay with YAP1 signaling and reduced mitochondrial activity during NED. Our findings reveal that the divergent behavior of a single organelle defines binary pan-cancer classes, highlighting therapeutic opportunities with broad relevance.

## Results

### Castration-resistant patients present PC in NE cells

**Figure 1:**
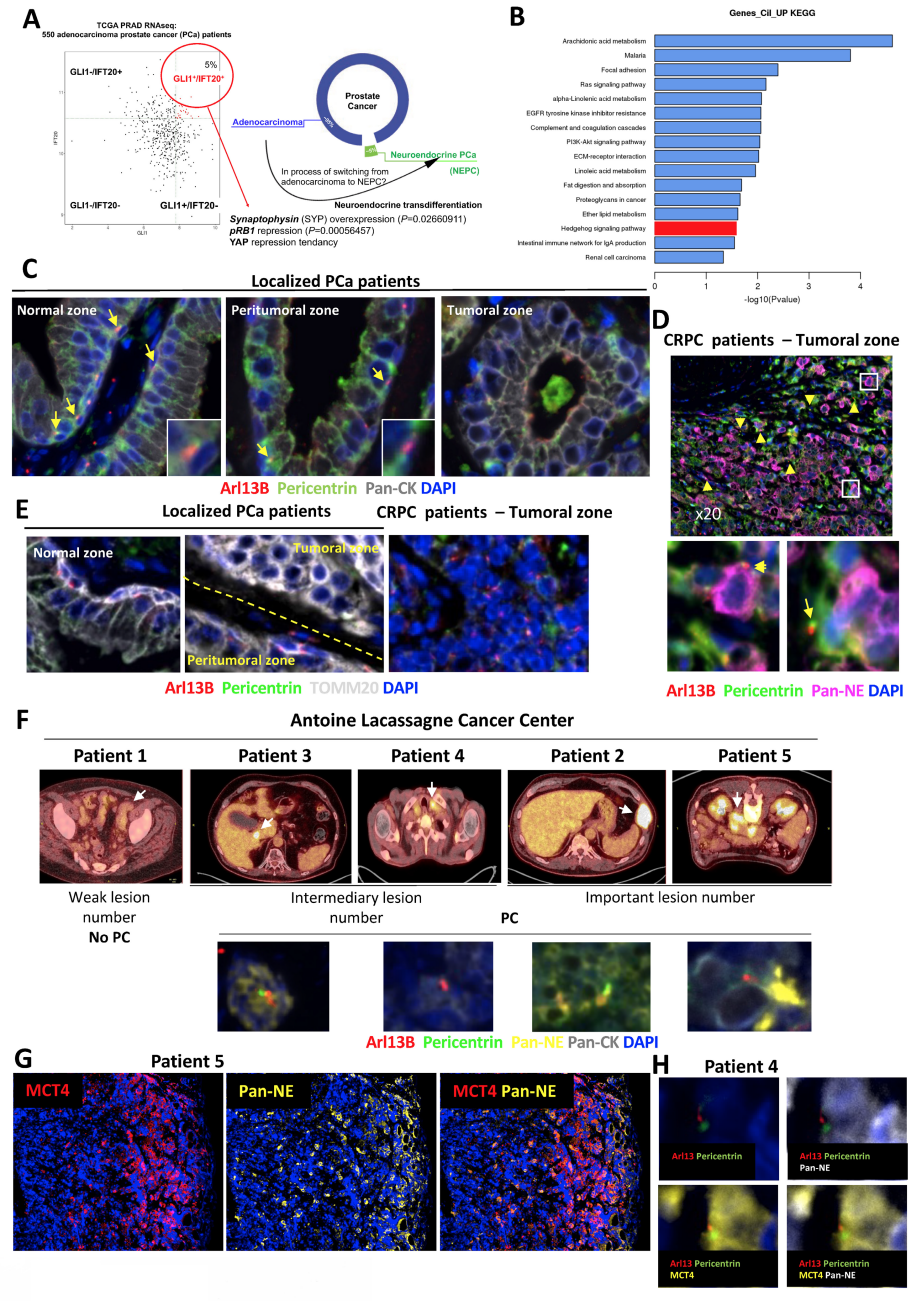
** Castration-resistant patients present primary cilia in neuroendocrine cells.** Schematic diagram illustrating the distribution of GLI1 and IFT20 expression among 550 prostate cancer patients from the TCGA-PRAD RNASeq dataset. The 5% patients expressing a GLI1+/IFT20+ signature also significant overexpression of Synaptophysin (SYP) and repression pRB1. YAP1 expression showed a tendency. (**B**) Histogram of the top 20 KEGG pathways from down-regulated genes from patients expressing GLI1^+^/IFT20^+^. (**C-D**) Representative immunofluorescence of localized PCa patients (n=5) (**C**) and CRPC patients (n=4) (**D**). Immunofluorescence labeled with (**C**) Pericentrin (green), Arl13b (red), pan-CK (grey) and DAPI (blue) of normal, peritumoral and tumoral zones and (**D**) Pericentrin (green), Arl13b (red), Pan-NE (pink) and DAPI (blue) of tumoral zone. Samples were studied to evaluate the prediction model of the absence or presence of the primary cilium. (**E**) Immunofluorescence labeled with Pericentrin (green), Arl13b (red), TOMM20 (grey) and DAPI (blue) of normal, peritumoral and tumoral zones in localized PCa patients and CRPC patients. Samples were studied to evaluate the prediction model of the absence or presence of the primary cilium. (**F**) *Top*, Maximum intensity projection FDG-PET images showing hypermetabolic metastatic lesions in the baseline castrate-sensitive state for patients 1 to 5 from Centre Antoine Lacassagne. White arrows are showing the SUVmax. Lesion sites were as follows: Patient 1 – left iliac lymph node; Patient 2 – left 8th rib; Patient 3 – segment IV of the liver; Patient 4 – left supraclavicular lymph node; and Patient 5 – large peritoneal mass with calcifications and heterogeneous density. *Bottom*, Representative immunofluorescence images of primary cilia (PC) labeled with Pericentrin (green), ARL13B (red), pan-cytokeratin (Pan-CK, grey), pan-neuroendocrine markers (Pan-NE, yellow), and nuclei (DAPI, blue) are shown for patients 2, 4, and 5. (**G**) Patient 5 – Co-localization of MCT4 with pan-NE markers in CRPC tissues. Representative immunofluorescence staining of a CRPC patient section showing MCT4 (red), pan-NE (yellow), and DAPI (blue). (**H**) Patient 4 – Higher magnification images stained for Arl13b (red) and Pericentrin (green) together with pan-NE (yellow), illustrating the presence of primary cilia in NE-like regions.

To validate our hypothesis based on the presence of PC in a small particular group of PCa patients, we analyzed the *GLI1*/*IFT20* signature in the TCGA-PRAD database, comprising 550 PCa patients (Figure 1A). Application of this signature revealed that 5% of patients diagnosed with PCa as adenocarcinoma exhibited a positive *GLI1*^+^/*IFT20*^+^ signature, suggesting the potential presence of PC in this subset of patients. The small number of patients represented in this signature led us to question the possibility that the cancer in these patients was in transition between adenocarcinoma and NE phenotype cancer. Therefore, we examined specific markers of NE cells and observed that Synaptophysin (SYP) was significantly overexpressed (p = 0.027), pRB1 was significantly repressed (p = 0.0006), and YAP1 was repressed but only trend-wise as expected in an NE population. The characterization of these patients revealed that they predominantly had Gleason scores of 7 or 9, corresponding to intermediate- to high-grade tumors (Figure S1A). Subsequently, we analyzed the gene expression in tumors from *GLI1*^+^/*IFT20*^+^ patients compared to all other patients using a Volcano plot (Figure S1B). We observed that the Hedgehog signaling pathway driven by the primary cilium was overexpressed (Figure 1B). Moreover, a strong suppression of mitochondrial function and energy metabolism was observed in the enrichment analysis of downregulated genes (Figure S1). KEGG (Figure S1C) and Reactome (Figure S1D) analyses highlighted significant downregulation of pathways involved in oxidative phosphorylation, respiratory electron transport, and TCA cycle activity. Gene Ontology terms confirmed these findings, showing reduced expression of genes related to ATP synthesis, mitochondrial electron transport, and purine metabolism (Figure S1E).

To validate the presence of PC in NE prostate cancer cells, we analyzed prostate tissue samples from patients with localized PCa who had not received hormone therapy (n = 5) and from patients with castration-resistant PCa (CRPC, n = 4) from the Henri Mondor Hospital cohort (CPP16169) (Figure S1F). Among the CRPC cases, two contained neuroendocrine tumor cell contingents (ChGA⁺), for which additional immunofluorescence staining with ChGA, SYP, and TUBB3 was performed. In non-treated patients, we were able to observe the normal, peritumoral, and tumoral zones (Figure 1C). However, we only had access to the tumoral zones for CRPC patients. Using ARL13B (red) and Pericentrin (green) to stain primary cilia, panCK (grey) to detect keratins, which are structural proteins present in epithelial cells, and DAPI (blue) for the nucleus, we observed that the normal areas of all non-treated patients presented numerous PC at the level of basal cells of the prostate (Figure 1C). The peritumoral zone still exhibited cilia but in lesser quantities, while the tumoral zone did not present any PC. In the four CRPC patients, the NE panel, containing a mixture of antibodies directed against TUBB3, ChgA and SYP) was used to label NE cells (Figure 1D). PC were present in significant quantities in the four CRPC patients. PC were detected in NE tumor cells suggesting that ciliogenesis may precede neuroendocrine differentiation. Surprisingly, PC was detected also in tumor cells that did not yet express NE markers. TOMM20, a key component of the outer mitochondrial membrane translocase complex and a marker of mitochondrial mass and integrity, was used to assess mitochondrial status. Interestingly, in localized PCa patients, TOMM20 was expressed in basal cells within both normal and peritumoral zones, particularly in cells expressing PC (Figure 1E). However, in the tumoral zone, TOMM20 expression was higher, but without the presence of PC. In CRPC patients, TOMM20 expression was completely absent, suggesting noticeable changes in mitochondrial morphology and function, which may reflect an adaptation of the metabolic state. To investigate this hypothesis, we took advantage of FDG-PET scans, which uniquely provide information on tumor glucose metabolism, performed in a rare subset of patients with suspected NE prostate cancer and screened the database at the Antoine Lacassagne Cancer (CAL) Center. While NEPC patients are generally reported to exhibit high FDG uptake 34 due to enhanced glycolytic activity, our study could provide direct pathological confirmation and could demonstrate how this metabolic signature might correlate with histological subtype and prognosis. Such scans are not routinely conducted in advanced PCa, making this cohort particularly valuable. We identified ten individuals who met three main criteria: availability of FDG-PET scans, access to preserved tissue samples, and suggestive neuroendocrine features. Upon further review, five of these cases were excluded—either due to severely degraded material or a lack of exploitable tissue—leaving five patients suitable for downstream analysis. This small number reflects the limited availability of high-quality, clinically annotated samples that combine metabolic imaging and tissue-based characterization in suspected NE prostate cancers.

Initially, immunostaining with a NE marker cocktail (Pan-NE) and a pan-cytokeratin antibody (Pan-CK) revealed highly heterogeneous distributions of NE cells (Figure S2A-D). In patients 2 and 3, clearly distinct clusters of adenocarcinoma and NE cells were observed. In contrast, patients 4 and 5 exhibited small, patchy NE cell populations. Four of the five patients exhibited PC (patients 2, 3, 4, and 5) (Figure 1F and Figure S2). However, the highest number of PC was observed in patient 5. Interestingly, patient 4 showed primary cilia in areas that were not neuroendocrine. Because MCT4 drives lactate efflux and extracellular acidification, it constitutes a robust functional readout of glycolytic activity. We therefore performed MCT4 immunostaining on CRPC patient tissues from patients 4 and 5 (Figure 1G). Although quantification is challenging in heterogeneous clinical samples, MCT4 signal was clearly enriched in neuroendocrine-like tumor cells compared with adjacent adenocarcinoma components. Notably, we observed that primary cilia were consistently present in MCT4-positive NE cells (Figure 1H). Consistently, primary cilium–bearing neuroendocrine tumor cells were Ki67-negative, indicating that these ciliated NE cells correspond to a non-proliferative subpopulation (Figure S3A, B). By integrating FDG uptake as a marker of glycolytic activity, together with lesion count and PC presence (Tables 1 and 2), we proposed a stratification into two signatures reflecting different states of tumor aggressiveness and potentially distinct stages of transdifferentiation (Table 3). Signature 1, with few lesions and loss of the primary cilium, may reflect a slower-progressing form of disease that remains more controlled despite multiple treatments. In contrast, Signature 2, defined by high lesion burden and preserved cilium expression, marks a highly plastic and aggressive state, prone to therapeutic resistance and neuroendocrine differentiation. Lesion count thus complements FDG uptake as a metabolic indicator, while cilium status emerges as a potential marker of tumor plasticity and progression, warranting validation in larger cohorts.

Taken together, analyses of the TCGA, Henri Mondor and CAL cohorts show that primary cilia are mainly detected in late-stage CRPC, mainly in tumors with NE features and reduced mitochondrial gene expression, consistent with a metabolic shift away from oxidative phosphorylation. Their progressive acquisition may act as both a marker and a driver of tumor plasticity and aggressive transformation, highlighting the PC as a potential biomarker and therapeutic target.

### The PC is only expressed in NE cells *in vitro*

**Figure 2:**
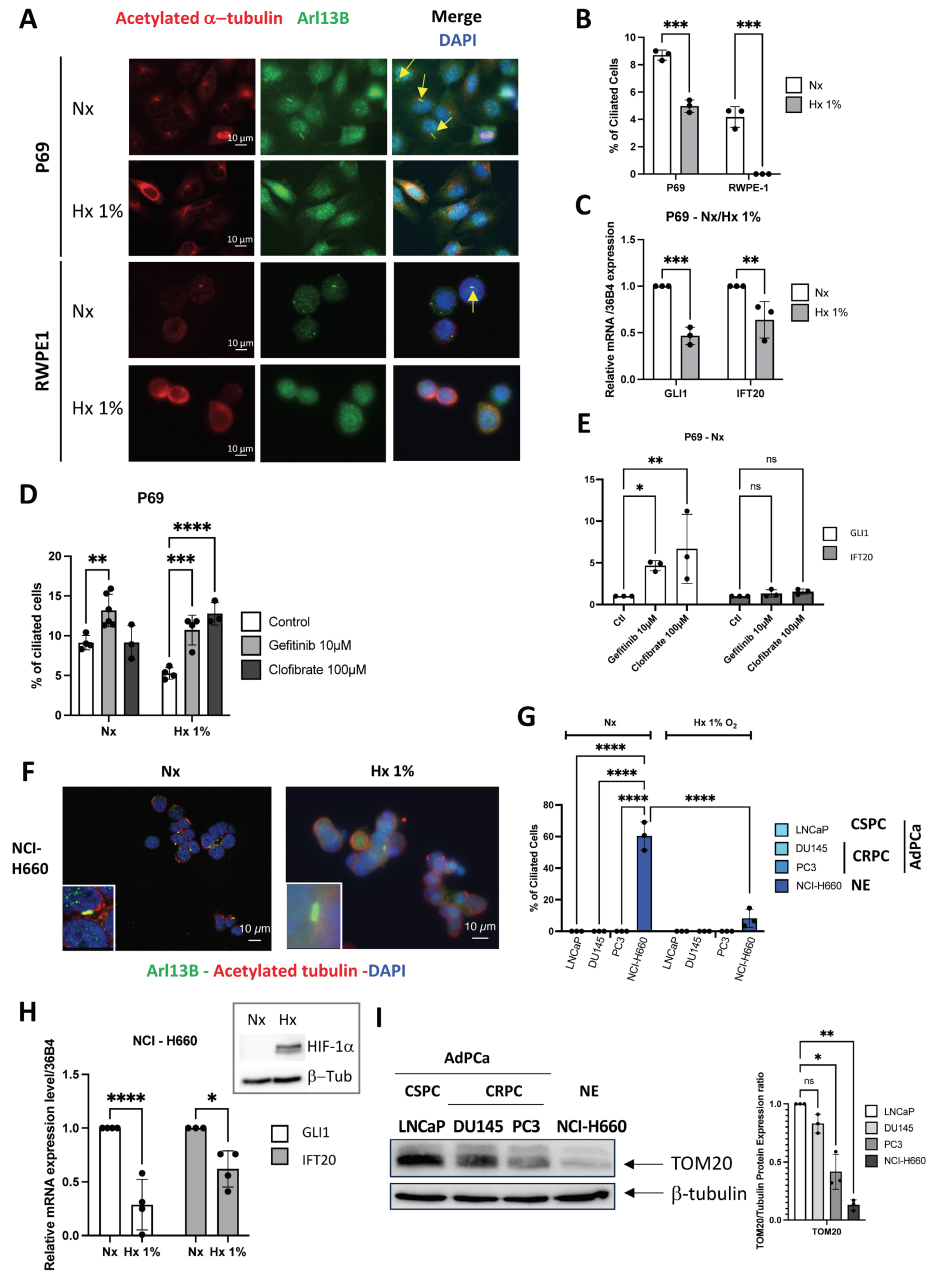
** Primary cilium is only expressed in NCI-H660 cells *in vitro.*** (**A**) Immunofluorescence of P69 and RWPE1 cells exposed to normoxia (Nx) and hypoxia (Hx – 1% O_2_) for 72h and labeled with acetylated α-tubulin (acetylated α-tub.) (red) and Arl13b (green) along with DAPI (blue) (magnification: x60, scale bar 10 µm). (**B**) Quantification of the percentage of ciliated cells in P69 and RWPE1 cell lines exposed to normoxia (Nx) and hypoxia (Hx – 1% O_2_) for 72h (n≥300). (**C**) Quantification of the transcriptional expression of GLI1 and IFT20 obtained through RT-qPCR conducted on P69 cells exposed to normoxia (Nx) and hypoxia (Hx – 1% O_2_) for 72h. Expression normalized to the control condition. (**D**) Quantification of the percentage of ciliated cells in P69 cells treated with Gefitinib (10µM) and Clofibrate (100µM) in normoxia (Nx) for 48h (n=250). (**E**) Quantification of the transcriptional expression of GLI1 and IFT20 obtained through RT-qPCR conducted on P69 cells exposed to normoxia (Nx) for 72h. Expression normalized to the control condition. (**F**) Immunofluorescence of NCI-H660 cells exposed to normoxia (Nx) and hypoxia (Hx – 1% O_2_) for 72h and labeled with acetylated α-tubulin (acetylated α-tub.) (red) and Arl13b (green) along with DAPI (blue) (magnification: x60, scale bar 10 µm). (**G**) Quantification of the percentage of ciliated cells in P69, LNCaP, DU145, PC3 and NCI-H660 cell lines exposed to normoxia (Nx) and hypoxia (Hx – 1% O_2_) for 72h (n≥300). Castration-Sensitive Prostate Cancer (CSPC), Castration-Resistant Prostate Cancer CRPC, Adenocarcinoma of the Prostate Cancer (AdPCa), Neuroendocrine (NE). (**H**) Quantification of the transcriptional expression of GLI1 and IFT20 obtained through RT-qPCR conducted on NCI-H660 cells exposed to normoxia (Nx) and hypoxia (Hx – 1% O_2_) for 72h. Expression normalized to the control condition. The inset confirms effective hypoxia through the stabilization of HIF-1a. (**I**) Immunoblot analysis of TOM20 protein expression in prostate cancer cell lines representing increasing tumor aggressiveness: LNCaP (CSPC), DU145 and PC3 (CRPC), and NCI-H660 (neuroendocrine). β-tubulin serves as a loading control. Right panel: densitometric quantification of TOM20 normalized to β-tubulin. Data are shown as mean ± SEM. All quantifications were performed using GraphPrism9 software. Statistical analysis: Two-way ANOVA; significant differences are indicated by * p < 0.05, ** p < 0.005, *** p < 0.0005, and **** p < 0.0001.

Considering the potential expression of the PC in NE cells, we investigated its dynamics throughout tumor progression, from normal cells to aggressive adenocarcinoma cells, and NE phenotypes. We then assessed whether the *GLI1*/*IFT20* signature could serve as a surrogate for PC detection, thereby avoiding systemic immunostaining. To this end, we tested conditions known to modulate ciliogenesis, including hypoxia (Hx), as previously reported in ccRCC^23^. Using normal prostate cells P69 and RWPE1, we visualized the PC with ARL13B and acetylated tubulin markers, revealing the presence of PC in 8.7±0.361% and 4.2±0.756% (Figure 2A-B), respectively. Notably, PC presence was significantly repressed under hypoxic conditions at 1% oxygen. Under hypoxic conditions, we observed decreased expression of both *GLI1* and *IFT20* in P69 cell line (Figure 2C). We then utilized both Gefitinib and Clofibrate to enhance PC expression in P69 cells as previously described^35^. These compounds were confirmed to be effective on their specific targets, HIF-1α and CPT1, respectively (Figure S4A-B). Both compounds could increase the percentage of ciliated cells in hypoxia (Hx 1%), while in normoxia (Nx) only Gefitinib induced a detectable increase in the percentage of ciliated cells (Figure 2D). However, only GLI1 mRNA expression was significantly induced in Nx and showed a trend in Hx 1%, while IFT20 mRNA expression remained unchanged (Figure 2E - Figure S4C).

Similarly, we examined the presence or absence of the PC in castration- sensitive (CSPC) LNCaP, castration-resistant (CRPC) DU145 and PC3, prostate adenocarcinoma cell lines, and in prostate neuroendocrine NCI-H660 cell line. Given the known fragility of NCI-H660 cells *in vitro*, cell viability was assessed under normoxic and hypoxic conditions and was maintained in both settings (Figure S4D), ruling out differential survival as a confounding factor in the analysis of PC expression. Only NCI-H660 cells exhibited a high percentage of PC (60.37±8.91%), while all other adenocarcinoma cells showed a null percentage of PC (Figure S4E and Figure 2F-G). As expected, PC in NCI-H660 cells was repressed in Hx 1%, and the expression of *GLI1* and *IFT20* was significantly reduced (Figure 2H). The use of Gefitinib (Figure S4F) and Clofibrate (Figure S4G-H) did not lead to the re-expression of the PC, indicating a significant limitation in these adenocarcinoma cells (Figure S4I-J). In line with the observations made in patient samples (Figure 1E), immunoblot analyses demonstrated a marked decrease in TOM20 protein levels along prostate cancer progression, thereby completing the characterization of these cell lines and indicating reduced mitochondrial content in aggressive and neuroendocrine cells (Figure 2I).

Based on these findings, GLI1 and IFT20 expression closely parallels PC status in PCa. PC were confined to NE NCI-H660 cells and strongly repressed in adenocarcinoma models. Hypoxia further reduced both PC and GLI1/IFT20, highlighting their role in dynamic ciliogenesis. Altogether, GLI1+/IFT20+ tumors may acquire PC during NED in advanced stages, thereby fostering aggressive progression.

### The exclusive absence of YAP1 expression alone does not lead to the re-expression of the PC in adenocarcinoma cells

**Figure 3:**
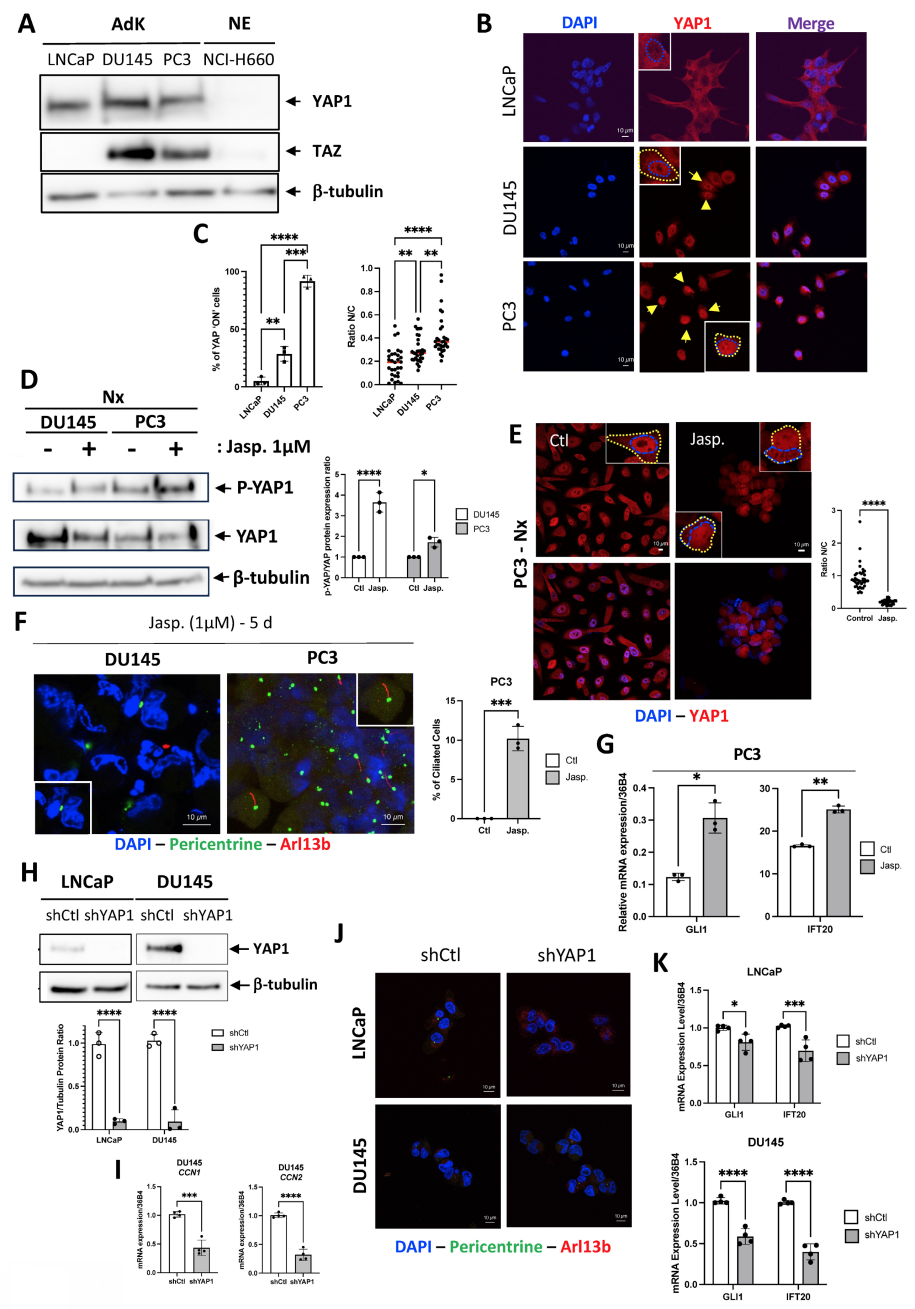
** Exclusive absence of YAP expression does not lead to the reexpression of the PC in adenocarcinoma cells.** (**A**) Cell lysates from LNCaP, DU145, PC3 adenocarcinoma (AdK) and NCI-H660 neuroendocrine (NE) cells were analyzed by immunoblot using YAP1 and TAZ proteins, along with b-tubulin as loading control. (**B**) Immunofluorescence of LNCaP, DU145 and PC3 cells labeled with YAP1 (red) and DAPI (blue) (magnification: x60, scale bar 10 µm). For clarity, nuclei are outlined with a blue dashed line, and whole-cell boundaries are delineated in yellow. (**C**) Quantification of YAP”ON” cells expressing YAP1 in the nucleus in LNCaP, DU145, PC3 cell lines. In addition, the nuclear-to-cytoplasmic intensity ratio was measured for each cell line to further assess YAP1 compartmentalization. (**D**) Cell lysates from DU145 and PC3 cells treated for 5 days with 1µM of Jasplakinolide (Jasp.) were analyzed by immunoblot using YAP1 protein, along with b-tubulin as loading control (left panel). The quantification of P-YAP1/YAP1 ratio in treated *versus* untreated DU145 and PC3 cell lines is also shown (right panel). (**E**) Immunofluorescence of PC3 cells treated for 5 days with 1µM of Jasplakinolide (Jasp.) and labeled with YAP1 (red) (magnification: x60, scale bar 10 µm). Quantification was performed across n=2 independent experiments (Control: 38 cells; Jasp.: 31 cells). (**F**) Immunofluorescence of DU145 and PC3 cells treated for 5 days (5 d) with 1µM of Jasplakinolide (Jasp.) and labeled with pericentrin (green) and Arl13b (red) along with DAPI (blue) (magnification: x60, scale bar 10 µm). (**G**) Quantification of the transcriptional expression of *GLI1* and *IFT20* obtained through RT-qPCR conducted on PC3 cells treated for 5 days with 1µM of Jasplakinolide (Jasp.). (**H**) Cell lysates from LNCaP and DU145 cells stably expressing shCtl or shYAP1 were analyzed by immunoblot using YAP1 protein, along with b-tubulin as loading control. Immunoblot (top) and quantification of YAP1 expression (bottom). (**I**) Immunofluorescence of LNCaP and DU145 cells transfected with shCtl or shYAP1 labeled with pericentrin (green) and Arl13b (red) along with DAPI (blue) (magnification: x60, scale bar 10 µm). (**J**) Quantification of the transcriptional expression of *GLI1* and *IFT20* obtained through RT-qPCR conducted on LNCaP (top) and DU145 (bottom) stably cells expressing shCtl or shYAP1 cells. Expression levels were normalized to the control condition (shCtl). All quantifications were performed using GraphPrism9 software. Statistical analysis: Two-way ANOVA; significant differences are indicated by * p < 0.05, ** p < 0.005, *** p < 0.0005, and **** p < 0.0001.

We have previously demonstrated that YAP1 plays a role in the absence of PC in cancer-like prostate cells 35. Based on these findings, we hypothesized that YAP1 expression could be pivotal not only in NED but also in the re-expression of PC. Because TAZ acts as a paralog and functional co-regulator of YAP withing the Hippo pathway, we also assessed its expression in LNCaP, DU145, PC3 (AdK), and NCI-H660 (NE) cells (Figure 3A). As expected, YAP1 was expressed only in Adk cell lines and not in NE cells. Conversely, TAZ was found to be absent in LNCaP and NCI-H660 cells, present in DU145 and PC3 cells. As a transcriptional co-activator, YAP1 must translocate into the nucleus to stimulate the transcription of its target genes. Therefore, we examined the localization of YAP1 by immunofluorescence in the three adenocarcinoma cell lines of prostate cancer (Figure 3B-C). Interestingly, YAP1 was absent from the nucleus in LNCaP cells, minimally present in the nuclei of DU145 cells, but fully localized in the nuclei and therefore active in PC3 cells. This pattern suggests a progressively increasing degree of YAP1 activity that parallels the aggressiveness of these prostate cancer cell lines. These observations are consistent with the quantitative analysis presented in Figure 3C, which shows a gradual increase in the percentage suggesting a progressive degree in the percentage of YAP1-positive cells across the three models, as well as by the nuclear-to cytoplasmic intensity ratio, which further confirms enhanced nuclear enrichment of YAP1. YAP1 was totally absent from the nucleus in NE cells (data not shown).

To mimic the absence of YAP1 in NCI-H660, we first used Jasplakinolide (Jasp.), an actin-stabilizing compound known to promote F-actin polymerization, and reported to modulate YAP1 cytoplasmic retention and phosphorylation, with context-dependent manner 36, 37. Our observations revealed a slight decrease in YAP1 protein levels in both DU145 and PC3 cells in parallel to an increase in the phosphorylated form of YAP1 (P-YAP1) (Figure 3D). This decrease was associated with a shift in YAP1 localization from the nucleus to the cytoplasm, compared to the control (Ctl) in PC3 cells (Figure 3E). Notably, we observed the presence of a few PC in DU145 cells and approximately 10.2±1.55% of ciliated cells in PC3 cells for the first time (Figure 3F). Finally, we noted significant increase in both GLI1 and IFT20 expressions following treatment with Jasp. (Figure 3G). To reinforce these results, we used LNCaP and DU145 cell lines already depleted for YAP1 38. At the protein level, YAP1 depletion proved to be almost total (Figure 3H). Importantly, LNCaP cells do not express detectable TAZ (Figure 3A), either at baseline or after YAP1 depletion, ruling out potential compensation by TAZ in this model. In contrast, DU145 cells express both YAP1 and TAZ, which may explain why YAP1 knockdown alone does not induce ciliogenesis in this context. YAP1 depletion reduced migration by 10% in LNCaP cells and by 80% in DU145 cells (Figure S5A-B). Interestingly, YAP1 silencing (shYAP1) in DU145 cells did not result in increased CCN1 or CCN2 mRNA expression, strongly suggesting the absence of compensatory TAZ activity (Figure 3I). Nonetheless, PC remained absent (Figure 3J) and both GLI1 and IFT20 expression were reduced in the absence of YAP1 in LNCaP and DU145 cells (Figure 3K). YAP1 was also invalidated in PC3 cells using siRNA. siRNAs used against YAP1 completely invalidated the YAP1 protein (Figure S5C-D), resulting in a total reduction in the migratory capacity of PC3 cells and thus their aggressiveness capacity (Figure S5E). However, PC still remained absent (Figure S5F). Similarly, knock-down of YAP1 alone, TAZ alone or both YAP1 and TAZ did not increase the proportion of ciliated cells (Figure S5G). YAP1 depletion did not allow the re-expression of SYP, one of the NE markers, demonstrating that PC3 cells had not reached a sufficient level of transdifferentiation to express the PC (Figure S5H). Notch4 mRNA, a potential marker of PC due to its role in differentiation and ciliary-related signaling, was detected under all PC-positive conditions but was significantly higher in NE cells (Figure S5I).

In conclusion, YAP1 alone may not fully explain the blockade of PC biogenesis. Although Jasp., considered a specific YAP1 inhibitor, restored PC in cells otherwise lacking it, this likely reflects additional effects beyond YAP1 inhibition.

### Jasplakinolide stabilizes microtubules and suppresses mitochondria to promote ciliogenesis

**Figure 4:**
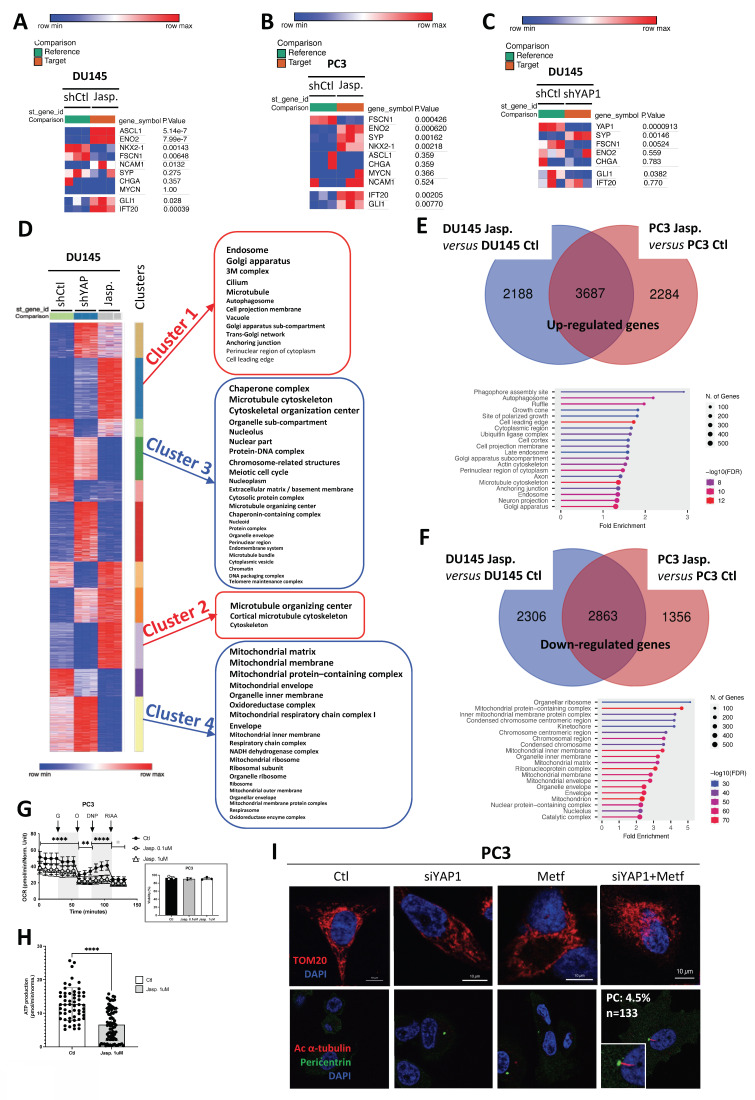
** Jasp. acts on microtubules while suppressing mitochondrial activity to induce PC.** (**A**) Heatmap showing the expression of selected NE genes (*CHGA, ENO2*, *FSCN1* and* SYP*) and PC-related genes (*GLI1*/*IFT20*) in DU145-shCtl cells compared to DU145-shCtl treated with 1µM of Jasplakinolide (Jasp.). Gene expression was analyzed using Phantasus (v1.19.3). (**B**) Heatmap showing the expression of selected NE genes (*CHGA, ENO2*, *FSCN1* and* SYP*) and PC-related genes (*GLI1*/*IFT20*) in PC3 cells in the absence (Ctl) or presence (Jasp.) of 1µM of Jasplakinolide. Gene expression was analyzed using Phantasus (v1.19.3). (**C**) Heatmap showing the expression of selected NE genes (*CHGA, ENO2*, *FSCN1, SYP* and YAP1) and PC-related genes (*GLI1*/*IFT20*) in DU145-shCtl cells compared to DU145-shYAP1. Gene expression was analyzed using Phantasus (v1.19.3). (**D**) Heatmap of genes in DU145-shCtl cells compared to DU145-shYAP1 and DU145-shCtl treated with 1µM of Jasplakinolide (Jasp.). K-means clustering performed using Phantasus (v1.19.3) identified 11 distinct clusters. Clusters 1 and 2 were enriched in upregulated genes associated with “Cellular components,” while Clusters 3 and 4 included downregulated components of similar categories. (**E**) Venn diagram (top) showing the differential distribution of the up-regulated genes detected between DU145 treated by Jasplakinolide (Jasp.) *versus* DU145 not treated (Ctl) cells (in blue) and between PC3 treated by Jasplakinolide (Jasp.) *versus* PC3 not treated (Ctl) cells (in blue). (Bottom) Gene set enrichment list of RNA-Seq data of the 3687 common up- regulated genes using “Cellular components”. (**F**) Venn diagram (top) showing the differential distribution of the down-regulated genes detected between DU145 treated by Jasplakinolide (Jasp.) *versus* DU145 not treated (Ctl) cells (in blue) and between PC3 treated by Jasplakinolide (Jasp.) *versus* PC3 not treated (Ctl) cells (in blue). (Bottom) Gene set enrichment list of RNA-Seq data of the 2863 common down-regulated genes using “Cellular functions”. (G) Mitochondrial respiratory control in PC3 cells. Oxygen consumption rate (OCR) was monitored in real time using the XF96 analyzer. Cells were cultured for 24 h in the absence (Ctl) or presence of jasplakinolide (Jasp.; 1 µM or 0.1 µM). After 1 h of glucose deprivation, glucose (G), oligomycin (O), DNP, and rotenone plus antimycin A (R/A) were sequentially injected at the indicated time points. OCR values were normalized to protein content after each experiment. The OCR profile shown is representative of three independent experiments. Ctl and Jasp. 1 µM conditions were analyzed in three independent experiments (n = 3), each performed with at least 16 technical replicates per condition. The Jasp. 0.1 µM condition was assessed in a single independent experiment (n = 1) and is shown for exploratory comparison only. (**H**) ATP production in PC3 cells. Mitochondrial ATP-linked respiration (ATP_ox_) was estimated based on oxygen consumption associated with ATP synthesis, using the XF96 analyzer. Graphs are representative of at least three independent experiments carried; each performed in octuplicate. Graphs are representative of three independent experiments (n = 3), each performed in octuplicate. Protein standardization was performed after each experiment. Statistical analysis was performed using two-way ANOVA. (**I**) Immunofluorescence of PC3 cells transfected with shCtl or shYAP1, treated with metformin (Metf, 5 mM), or transfected with shYAP1 and treated with metformin (Metf, 5 mM). Cells were labeled with pericentrin (green) and Arl13b (red) along with DAPI (blue) (magnification: x60, scale bar 10 µm). PC% indicates the percentage of primary cilium–positive (ciliated) cells, quantified by manual counting (n = 133 cells). Quantification was performed by manual counting in two independent experiments (Exp1: 5/133 cells; Exp2: 9/200 cells), corresponding to 4.1% ± 0.5 SD (n = 2). All quantifications were performed using GraphPrism9 software. Statistical analysis: Two-way ANOVA; significant differences are indicated by **** p < 0.0001.

To dissect the mechanisms underlying primary cilium regulation, we compared the transcriptional programs induced by YAP1 knockdown and Jasp. treatment through RNASeq in DU145 cells, complemented by analyses in PC3 cells. We first confirmed that Jasp. inhibits YAP1 activity through reduced CCN1 and CCN2 expression in DU145 cells (Figure S6A). Prolonged Jasp. treatment did not induce a full NE phenotype but instead triggered partial NE-like features, including robust ENO2 upregulation and FSCN1 repression in both DU145 and PC3 cells, together with more variable changes in SYP and CHGA expression (Figure 4A-B). Importantly, Jasp. increased GLI1/IFT20 expression in the presence of PC. In contrast, YAP1 knockout also promoted NE-like features but did not induce GLI1/IFT20 expression (Figure 4C), consistent with the absence of PC previously described. Interestingly, DU145 cells displayed a modest but significant induction of SYP mRNA upon YAP1 depletion, while they did not in PC3 cells (Figure S5H) highlighting that the effect of YAP1 loss on NE markers is cell line–dependent.

We then compared the two conditions, shYAP1 and Jasp., in DU145 cells and searched for clusters of genes that were oppositely regulated under these conditions (Figure 4D). Four distinct clusters of genes emerged: two clusters (1 and 2) were upregulated with Jasp. treatment (red), while two clusters (3 and 4) were downregulated (blue). Clusters 1 (Table 4) and 2 (Table 5) expressed more genes related to the PC (cilium, microtubule, cortical microtubule cytoskeleton, microtubule organizing center, centrosome), whereas cluster 3 (Table 6) also seemed to affect genes involved in the chromosome, condensed chromosome, and microtubule cytoskeleton, among others. Cluster 4 (Table 7), on the other hand, appeared to downregulate genes involved in mitochondrial function (mitochondrial respiratory chain complex I, mitochondrial protein-containing complex, mitochondrial matrix...). Similarly, a 5-day treatment with Jasp. on PC3 cells clearly highlighted numerous clusters affecting all the components necessary for PC biogenesis (Figure S6B). As the presence of PC was revealed under Jasp. treatment in both DU145 and PC3 cells, we then compared these two conditions to uncover similarities. A total of 3687 upregulated genes appeared in common between the two cell lines. Not surprisingly, axon and microtubule cytoskeleton emerged among the list of cellular components identify with ShinyGo (Figure 4E). Among the 2863 repressed genes common to DU145 and PC3, genes involved in mitochondrial protein-containing complex, inner mitochondrial membrane protein complex, mitochondrial inner membrane, mitochondrial matrix, mitochondrial membrane, and mitochondrion were characterized, strongly suggesting that the repression of mitochondrial activity could be at the core of Jasp.'s action in PC biogenesis (Figure 4F). To assess the impact of Jasp. on mitochondrial function, we measured oxygen consumption in PC3 cells using the Seahorse XF analyzer. Jasp. treatment markedly reduced overall respiration and mitochondrial ATP production, confirming impaired respiratory capacity (Figure 4G-H), without affecting cell viability under these conditions. To test whether both YAP1 inhibition and impaired mitochondrial function are required for PC induction, PC3 cells were treated with siYAP1, Metformin, or their combination. Metformin, a complex I inhibitor that suppresses mitochondrial oxidative phosphorylation, and Rotenone, a classical and potent inhibitor of mitochondrial complex I, were used to pharmacologically impair mitochondrial respiration. While each treatment alone slightly altered the mitochondrial network, the combined treatments induced heterogeneous mitochondrial morphologies, ranging from a punctate pattern to a more condensed perinuclear distribution. Importantly, PC formation was observed specifically when Metf or rotenone was combined with siYAP1 (Figure 4I, Figure S6C). Mitochondrial integrity was further assessed by FACS analysis of TOMM20 expression and by measuring membrane potential (ΔΨm) with a fluorescence-based assay (Figure S6D). YAP1 silencing induced a marked loss of ΔΨm, whereas Metf alone had only a limited effect, and the combination was not additive. However, imaging revealed pronounced mitochondrial fragmentation under the combined treatment, pointing to a structural–functional dissociation characteristic of mitochondrial stress.

In conclusion, YAP1 is not the sole barrier to the presence of primary cilia in Adk cells, but our results suggest a potential contribution of mitochondrial activity in controlling the biogenesis of the PC. Their combined influence may not only govern structural features such as ciliogenesis but also shape lineage identity, reinforcing a cellular state that resists differentiation and supports tumor progression.

### PC drives cell migration in NCI-H660 and is regulated by mitochondrial inactivity

**Figure 5:**
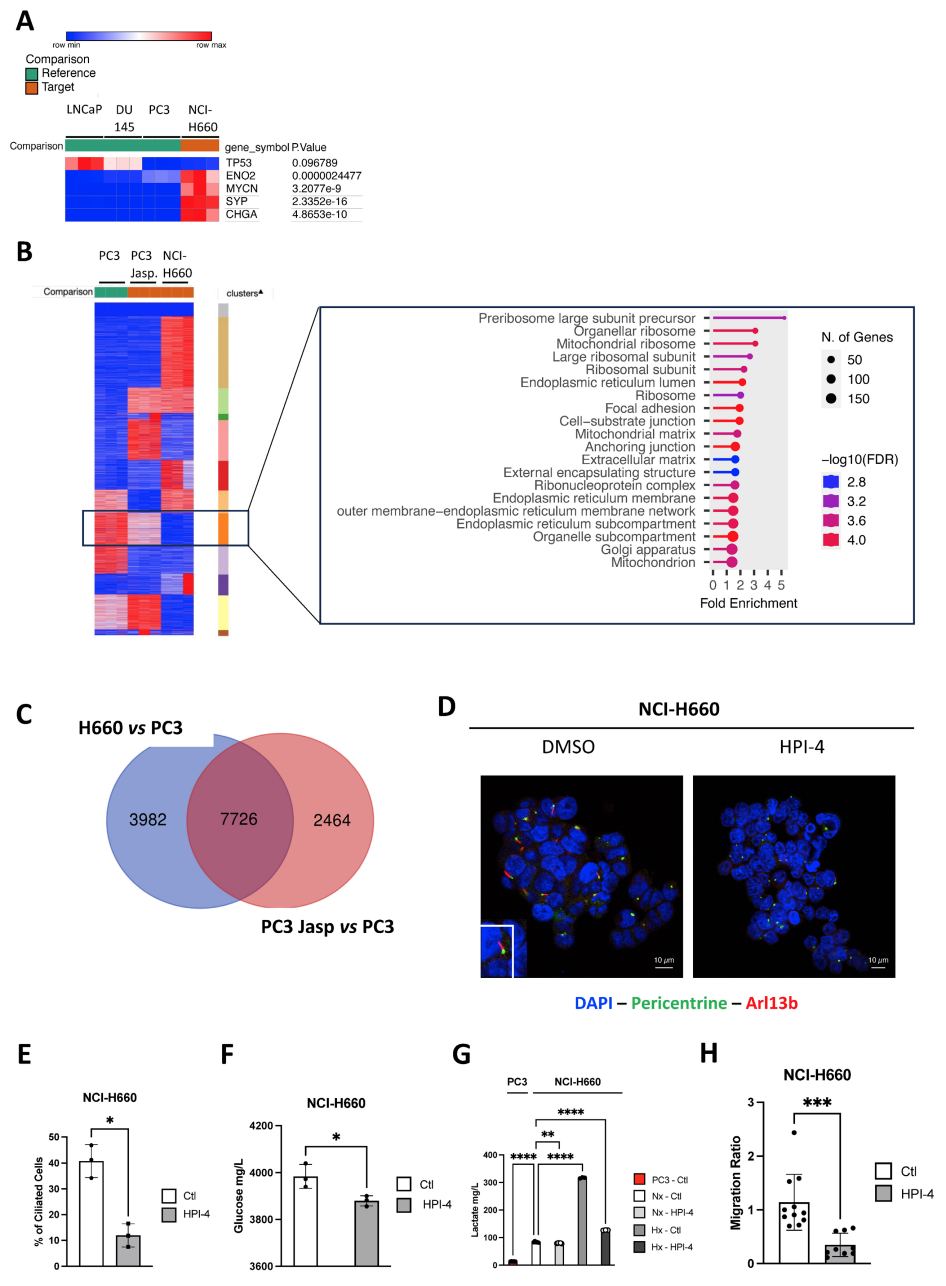
**PC drives aggressiveness in NCI-H660 and is regulated by mitochondrial inactivity.** (**A**) Heatmap showing the expression of selected NE genes (*CHGA, ENO2*, *MYCN*, *SYP and TP53*) in LNCaP, DU145 and PC3 cells compared to NCI-H660. Gene expression was analyzed using Phantasus (v1.19.3). (**B**) Heatmap of differentially expressed genes between cells expressing primary cilium (PC3 cells treated with Jasp. and NCI-H660) and control PC3 cells. Eleven clusters were characterized using K-means *via* Phantasus (v1.19.3) and are indicated on the right. The boxed panel shows the gene set enrichment analysis (GSEA) based on RNA-Seq data, highlighting “Cellular Component” terms enriched in ciliated *versus* non-ciliated cells. (C) Venn diagram showing the overlap of differentially expressed genes associated with “Cellular Component” terms between (i) NCI-H660 and PC3 cells treated with Jasplakinolide (Jasp.) *versus* untreated PC3 cells (blue), and (ii) PC3 cells treated with Jasp. *versus* untreated PC3 cells (blue). (**D**) Immunofluorescence of NCI-H660 cells treated for 48h HPI-4 and labeled with pericentrin (green) and Arl13b (red) along with DAPI (blue) (magnification: x60, scale bar 10 µm). (**E**) Quantification of the percentage of ciliated cells in NCI-H660 cell lines treated with HPI-4 for 48h (n≥300). (**F-G**) NCI-H660 cells were cultured for 48 h under normoxic (Nx) or hypoxic (Hx) conditions, in the absence (Ctl) or presence of HPI-4. The supernatant from PC3 cells was used as a control for OXPHOS-active cells. Glucose (**F**) and lactate (**G**) concentrations (mg/L) were measured in the culture supernatants and normalized to cell number. (**H**) Migration of NCI-H660 cells, either untreated (Ctl) or treated with HPI-4 for 48 h, was assessed using a Boyden chamber assay. All quantifications were performed using GraphPrism9 software. Statistical analysis: Two-way ANOVA; significant differences are indicated by * p<0.05, ** p<0.005, *** p<0.0005, and **** p< 0.0001.

To understand the role of PC in NE cells generally and specifically in NCI-H660, we initially assessed the expression of NE markers (*TP53*, *ENO2*, *MYCN*, *SYP*, and *CHGA*) in NCI-H660 compared to Adk cells. As anticipated, all markers exhibited significant expression in NCI-H660 compared to LNCaP, DU145, and PC3 (Figure 5A). Upon global comparison of PC3, Jasp.-treated PC3, and NCI-H660 cells, we identified a distinct cluster that was highly expressed in PC3, reduced in PC3 treated with Jasp., and absent in NCI-H660 (Figure 5B). This cluster represents a decreasing expression trend across conditions with increasing ciliation. Functional annotation with ShinyGo linked it mainly to mitochondrial components (mitochondrial ribosome, matrix, and mitochondrion) as well as ribosomes (Figure 5B). To further pinpoint genes potentially involved in PC biogenesis, we then compared the transcriptomic changes occurring in two conditions characterized by the presence of a well-developed or reinforced ciliary phenotype: NIC-H660 cells (highly ciliated) *versus* PC3 cells and Jasp.-treated PC3 cells (partially ciliated) *versus* untreated PC3 cells. The overlap shown in the Venn diagram (Figure 5C) represents genes that are differentially expressed in both conditions, and therefore constitute shared transcriptional changes associated with enhanced ciliogenesis. A total of 7726 genes distributed across 61 cellular components confirmed the predominance of mitochondrial components such as Mitochondrion, Mitochondrial envelope, Mitochondrial membrane, Mitochondrial inner membrane, Mitochondrial matrix, and Mitochondrial protein complex, as well as components related to the Microtubule cytoskeleton, Microtubule organizing center, and Centrosome (Table 8). To test the functional link between ciliogenesis and metabolism, we inhibited PC formation with HPI-4, a hedgehog pathway inhibitor. HPI-4 treatment significantly reduced the percentage of ciliated cells (Figure 5D-E). Metabolic analysis with the YSI platform showed that, under normoxia, NCI-H660 cells consumed glucose but produced less lactate, suggesting a preferential use of pyruvate by mitochondria (Figure 5F-G). In the presence of HPI-4 in Nx, NCI-H660 cells consumed glucose but produced significantly less lactate, suggesting that pyruvate was utilized by the mitochondria. This potentially indicates a restoration of mitochondrial activity associated with the disappearance of PC. Moreover, NCI-H660 cells produced 10 times more lactate than PC3 cells, indicating a higher glycolytic profile. To further investigate the metabolic impact of HPI-4, we analyzed lactate production under hypoxic conditions, where glycolysis is strongly increased. In this context, HPI-4 induced a marked reduction in lactate production (≈60%) (Figure 5G), coinciding with a drastic loss of primary cilia. This result indicates that the metabolic effect of HPI-4 becomes more apparent in a highly glycolytic state. Importantly, even when ciliation was nearly abolished, lactate production was not completely suppressed, as expected, since the primary cilium contributes to, but does not exclusively control, glycolytic output. Metabolically, NCI-H660 cells show a profound inability to utilize Krebs cycle intermediates such as pyruvate, citrate, or succinate, pointing to defective mitochondrial oxidative metabolism (Figure S7A-N). In contrast, they preferentially consume phosphorylated sugars like glucose-6-phosphate, suggesting rerouted glycolysis and pentose phosphate pathway activation. This metabolic shift is consistent with a highly plastic metabolic reprogramming toward aerobic glycolysis (Warburg effect). Conversely, PC3 cells retain full metabolic flexibility, efficiently metabolizing all tested substrates, in line with functional mitochondria and greater adaptability. Ultimately, NCI-H660 appeared to exhibit reduced migratory capacity, concomitant with diminished PC presence (Figure 5H).

### Primary cilia emerge during transdifferentiation, coupled to mitochondrial collapse

**Figure 6:**
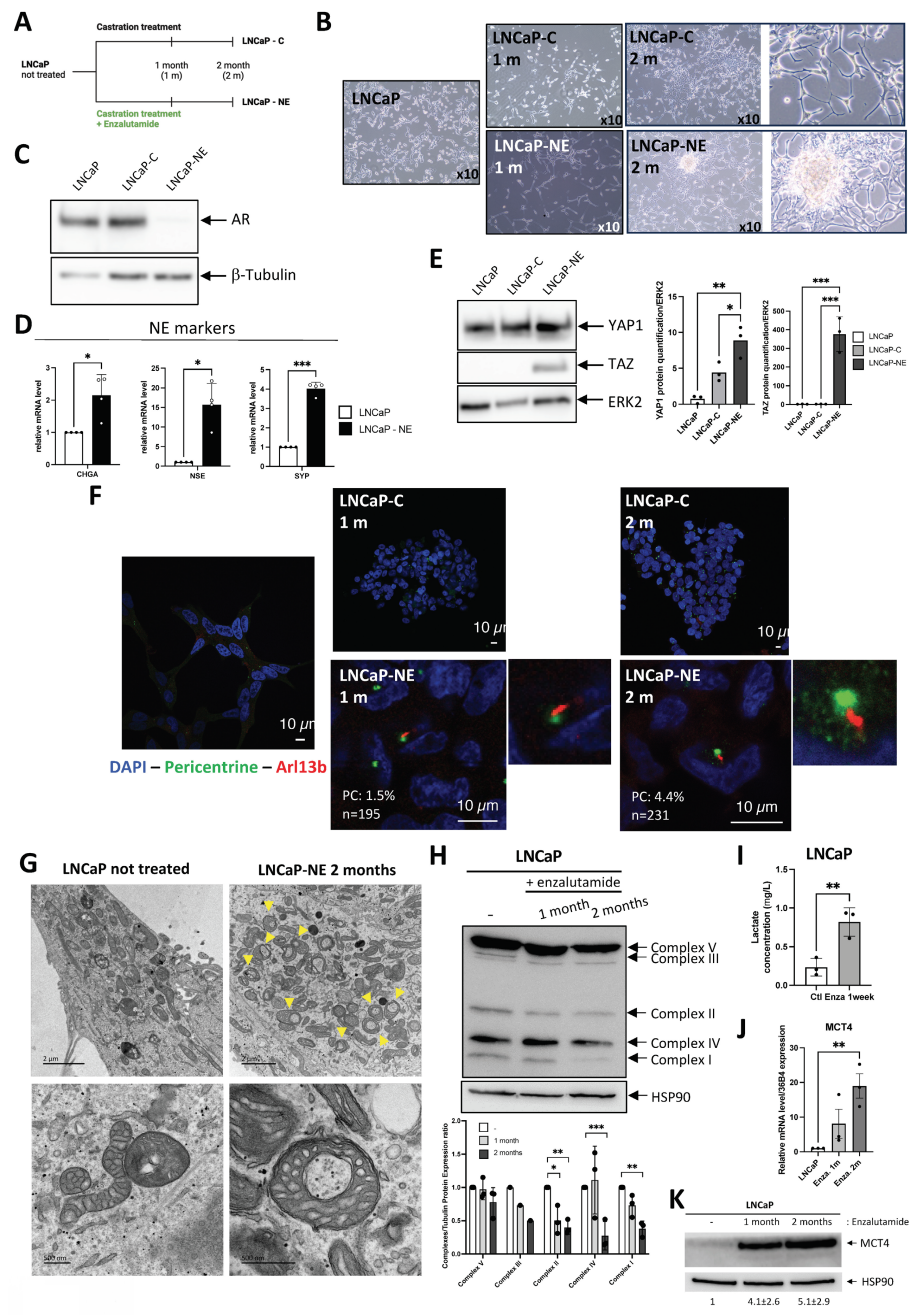
** In the LNCaP model treated with enzalutamide, forced neuroendocrine transdifferentiation facilitates a partial restoration of the primary cilium.** (**A**) Schematic protocol outlined to induce neuro-transdifferentiation of LNCaP cells. LNCaP cells are subjected to castration treatment (green). Following a 2-month treatment duration, cells are designated as LNCaP-C. LNCaP cells treated with castration treatment in the presence of Enzalutamide (red) are labeled as LNCaP-NE. (**B**) Microscopic images (10x magnification) of LNCaP cells untreated, as well as those treated for 1 month or 2 months following the protocol outlined in (A). (**C**) Cell lysates from LNCaP, LNCaP-C and LNCaP-NE cells treated for 2-months with castration treatment in the presence of Enzalutamide were analyzed by immunoblot using AR protein, along with b-Tubulin as loading control. (**D**) Relative expression levels of neuroendocrine markers CHGA, NSE, and SYP in LNCaP cells (white bars) and LNCaP-NE cells (black bars) after enzalutamide treatment. Gene expression was measured by RT-qPCR and normalized to RPLP0. Data represent mean ± SD from four independent experiments. (**E**) Immunoblot analysis of YAP1 and TAZ protein levels in cell lysates from LNCaP, LNCaP-C, and LNCaP-NE cells treated for 2 months with castration conditions in the presence of enzalutamide. ERK2 was used as a loading control. (**F**) Immunofluorescence of LNCaP cells untreated or treated for 1 month or 2 months according to the protocol described in (A). Cells were stained for pericentrin (green) and Arl13b (red), and nuclei (DAPI, blue) (magnification: x60, scale bar 10 µm). The percentage of primary cilium–positive cells (PC%) corresponds to the proportion of ciliated cells and was quantified by manual counting on n = 195 cells at 1 month and n = 211 cells at 2 months. (**G**) Transmission electron microscopy (TEM) images of LNCaP and LNCaP-NE cells. Representative ultrastructural views show normal mitochondrial morphology in LNCaP cells, while LNCaP-NE cells display altered mitochondria with a characteristic donut-shaped structure (inset; scale bar at 500 nm). Main image scale bar: 2 µm. (**H**) Immunoblot analysis of mitochondrial respiratory chain complexes (CI–CV) in NCI-H660 cells cultured under normoxic (Nx) or hypoxic (Hx – 1% O_2_) conditions for 48 h. Antibodies against subunits of complexes I to V were used to assess the impact of hypoxia on mitochondrial protein expression. Hsp90 served as a loading control. The experiment was performed in three independent biological replicates (n = 3), and the corresponding densitometric quantification is shown. (I) Lactate concentration measured in the culture medium of LNCaP cells under control conditions (Ctl) or after 1 week of enzalutamide treatment (Enza 1 week). Data are presented as mean ± SEM from three independent experiments. (**J**) Relative MCT4 expression in LNCaP treated with Enzalutamide (Enza.) compared with vehicle control. Bars represent mean ± SEM from n = 3 independent experiments; dots indicate individual biological replicates. Statistical significance was assessed using an unpaired two-tailed t-test; p < 0.05. (K) Representative immunoblot showing MCT4 expression in LNCaP cells treated with enzalutamide for 1 month and 2 months, compared with untreated control (–). HSP90 was used as a loading control. Numbers below indicate relative MCT4 protein levels normalized to HSP90 (mean ± SD). All quantifications were performed using GraphPrism9 software. Statistical analysis: Anova; significant differences are indicated by * p < 0.05, ** p < 0.005, and *** p < 0.0005.

Androgen-dependent LNCaP cells provide a relevant in vitro model to study therapy response. We simulated treatment by applying castration without treatment (LNCaP-C) or combined with the AR antagonist enzalutamide (LNCaP-NE), a standard therapy for castration-resistant prostate cancer (Figure 6A). The morphology of the cells was observed after one and two months of treatment (Figure 6B). After two months of treatment, LNCaP-NE displayed a neuronal phenotype with elongated extensions, along with a loss of AR protein expression and gene expression dependent on the AR markers (Figure 6C and Figure S8A), and upregulation of NE markers such as CHGA, NSE and SYP at the mRNA level (Figure 6D). The increase in YAP1 expression during the acquisition of a NE-like phenotype (Figure 6E) was comparable to the elevated YAP1 expression observed in PC3 cells (Figure 3A), which are known to exhibit a more aggressive YAP1-high phenotype. TAZ expression was also detected in LNCaP-NE cells (Figure 6E), mirroring its presence in PC3 cells (Figure 3A). In parallel, we examined the presence of PC. Few PC were detected only in LNCaP-NE as early as the first month (Figure 6F). Subsequently, we conducted RNAseq analysis on samples from LNCaP-NE after two months of treatment compared to LNCaP. Several neuroendocrine markers from LNCaP-NE exhibited significant changes, including *ENO2*, *SYP*, and *MYCN*, with trends also observed in *YAP1*, *TP53*, *NCAM1*, and *CHGA* compared to LNCaP (Figure S8B). Similarly, *GLI1*, *ARL13B*, and *IFT80* exhibited significant increases. Surprisingly, the expression of *IFT20* and *IFT88* was significantly decreased (-71% and -44% respectively; Figure S8B), which could explain the low percentage of PC observed. Not all NE trans-differentiation markers or PC markers detected in NCI-H660 cells were present in LNCaP-NE. Thus, although NE trans-differentiation was initiated, the process was clearly incomplete (Figure S8C).

Altogether, these results suggest that the presence of PC supports a glycolytic metabolic shift, and that their loss may relieve this constraint, potentially allowing the restoration of mitochondrial oxidative phosphorylation, in line with the observations made in patient cohorts.

Could enzalutamide in combination with castration treatment also affect mitochondrial activity? Electron microscopy analysis showed that after two months of treatment, the mitochondria exhibited a characteristic "donut"-shaped morphology a ring-like structure described in the literature in the context of mitochondrial stress or remodeling 39, 40 (Figure 6G). Mitochondrial morpho-functional parameters were extracted using the EmitoMetrix 41 pipeline from transmission electron microscopy images acquired at a spatial scale of 500 nm. Features describing mitochondrial size, shape, intensity, and cristae orientation were z-score–normalized using StandardScaler and visualized using a radar plot to compare control cells with cells treated with enzalutamide for 2 months, with values representing deviations from the global mean expressed in standard deviation units (Figure S9A). Compared with control cells, enzalutamide-treated cells exhibited reduced size-related parameters (Area, Perimeter, Feret diameter), increased shape compactness and regularity (Roundness, Circularity, Solidity), and a marked reorganization of cristae orientation descriptors. In parallel, intensity-related features indicated a more homogeneous mitochondrial signal distribution, consistent with a structurally remodeled and more compact mitochondrial phenotype following long-term enzalutamide treatment. Consistently, SHAP analysis (Figure S9B) identified mitochondrial intensity- and shape-related parameters as the main contributors to the prediction of enzalutamide treatment, with higher mean and median intensities, reduced intensity heterogeneity, and increased mitochondrial compactness contributing positively to the model output. Consistently, immunoblot analysis showed a marked reduction in the expression of mitochondrial complexes I, II and IV, with a decreasing trend also observed for complexes III and V, in enzalutamide-treated LNCaP cells (Figure 6H), supporting a disruption of mitochondrial oxidative phosphorylation. RNASeq comparisons between LNCaP-NE and LNCaP cells showed a clear collapse in the expression of nearly all genes involved in mitochondrial respiration (Figure S8D) like what was previously observed in NCI-H660. A large portion of genes involved in mitochondrion (Figure S8E), mitochondrial envelope (Figure S8F), respirasome (Figure S8G), and inner mitochondrial membrane protein complex (Figure S8H) are repressed in LNCaP-NE, strongly suggesting a mitochondrial deficiency along the transdifferentiation process. Consistently, enzalutamide-treated LNCaP cells exhibited significantly higher extracellular lactate levels after one week of treatment compared with control cells (Figure 6I), further supporting the idea of a metabolic reprogramming toward increased lactate production. To reinforce these observations, we analyzed the regulation of MCT4 expression following enzalutamide (Enza.) treatment. As shown, MCT4 expression was significantly increased in response to Enza at both the mRNA and protein levels (Figure 6J,K), consistent with the well-established role of MCT4 as an HIF-responsive lactate exporter in highly glycolytic cancer cells 42-44. These findings are in line with metabolic adaptations described in tumor cells exposed to stress conditions, where enhanced glycolytic flux and lactate export contribute to cell survival and tumor microenvironment remodeling. Analysis of the GSE197780 dataset 45, which includes 43 patients before and after three months of enzalutamide treatment, further supported our findings (Figure S10). AR markers showed a general decrease**,** while NEPC markers increased in parallel (Figure S10A). Stratification of post-treatment patients according to YAP1 expression further confirmed that, although all samples retained detectable YAP1 levels, only YAP1-high tumors showed a significant increase in early NEPC markers such as NCAM1 and ENO2, consistent with an intermediate yet incomplete NE state (Figure S10B). PC markers were consistently upregulated following enzalutamide treatment (Figure S10C). The impact on mitochondrial envelope markers was more variable and partial (Figure S10D).

Taken together, these results highlight that NE transdifferentiation, even in its intermediate, not fully established form, requires absence of AR markers and is associated with mitochondrial deficiency, ultimately permitting the emergence of some ciliated cells. These findings also emphasize that the molecular features accompanying ciliogenesis are highly context-dependent, supporting the need to adapt ciliary signatures, such as GLI1⁺/IFT20⁺/IFT80⁺, to the specific biology of prostate cancer.

### Single-cell sequencing identifies a ciliogenesis program in prostate cancer

We conducted a comprehensive single-cell RNA sequencing analysis by pooling and integrating datasets from Song *et al. 46* and Dong *et al. 47*, to generate a more complete and comparative transcriptomic profile. From the Song *et al.* dataset (GSE176031), single cell data based on adjacent non-tumor tissue served as the control group. These were integrated with 21,292 cells obtained from biopsies of 6 castration-resistant prostate cancer cases - 3 non-NE and 3 NE- as reported by Dong *et al.* (GSE 137829) 47. Employing the same signature as Dong e*t al.* for Epithelial, AR, and NE, we supplemented it with our own signature for the PC. Given that *IFT20* expression was not as robust as anticipated, we enhanced our signature by incorporating *GLI1*, *IFT20*, *ARL13B*, *IFT80*, and *IFT88*. Our new signature was initially evaluated using CiliaCarta 48 (https://tbb.bio.uu.nl/john/syscilia/ciliacarta/) (Table 9). According to CiliaCarta, our signature was accurate, with a slightly lower score for *GLI1* but satisfactory scores for the other four markers. Notably, *IFT88* emerged as the most reliable marker for detecting prostate cancer (PC).

**Figure 7:**
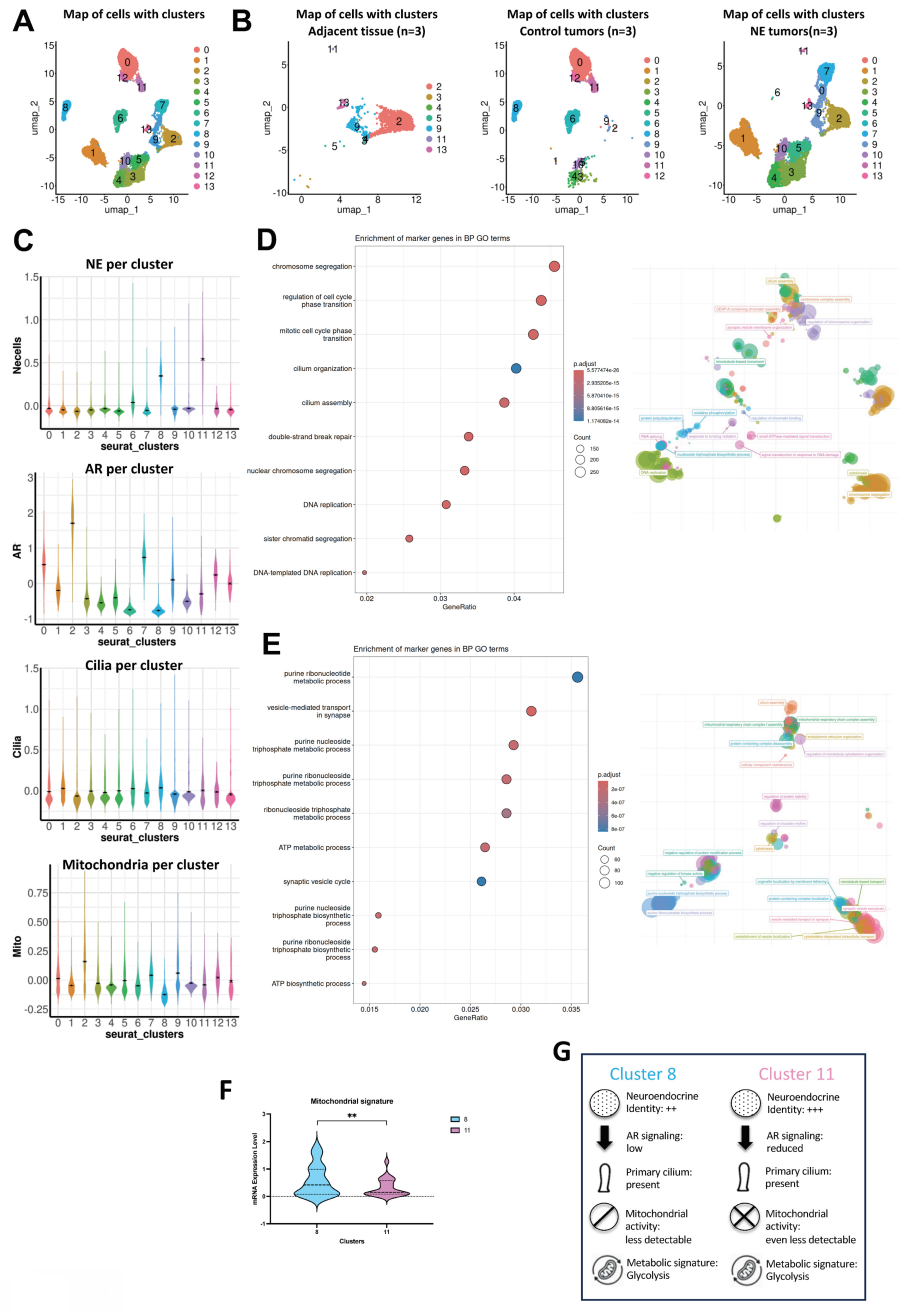
** Identification of primary cilium-enriched cell clusters by single-cell RNA-seq.** (**A**) UMAP plot showing the combined clustering of cells from adjacent tissue, control tumors, and neuroendocrine (NE) prostate tumors (n = 3 patients per condition). Each color corresponds to a distinct transcriptional cluster identified across the integrated dataset. (**B**) UMAP plots showing cell clustering from single-cell RNA-seq data of (left) adjacent normal tissue (GSE181294), (middle) control prostate tumors (GSE137829), and (right) neuroendocrine (NE) prostate tumors (GSE137829) (n = 3 patients per condition). Each color represents a distinct transcriptional cluster identified within each condition. (**C**) Violin plot showing the expression of gene signatures across transcriptional clusters. Expression levels of neuroendocrine (NE), androgen receptor (AR), primary cilium, mitochondrial gene sets across the 14 transcriptional clusters (0–13) identified by integrated single-cell RNA-seq analysis. Each color corresponds to a distinct cluster, highlighting heterogeneity in functional programs among tumor subpopulations. (**D-E**) GO Biological Process enrichment analysis of marker genes from Cluster 8 (D), and Cluster 11 (E). Left: Dot plots showing the top enriched GO biological processes for each cluster. Dot size indicates the number of genes associated with each term, and color reflects the adjusted p-value. Right: Enrichment maps displaying semantically related GO terms, illustrating key functional categories such as mitochondrial organization, oxidative phosphorylation, and vesicle-mediated transport, specific to each cluster. (**F**) Distribution of mitochondrial signature (ALDH1B1, ATP5MC2, ATP5PD, BAK, COX5A, NDUFA2, NDUFAB1, NDUFB1, NDUFB3, NDUFB4, NDUFB6, NDUFC1, NNT, SFXN2, TOMM20, VDAC1) scores across clusters 8 (blue) and 11 (pink). Each violin plot shows the probability density of the signature scores within each cluster. The solid line represents the median, and the dotted lines indicate the first and third quartiles. The width of each violin reflects the density of cells at different expression levels of the mitochondrial signature. Quantification was performed using GraphPrism9 software. Statistical analysis: 2way-Anova; significant differences are indicated by ** p=0.0025. (**G**) Schematic representation of the two neuroendocrine-enriched clusters identified by single-cell RNA-seq. Cluster 8 (blue), and Cluster 11 (pink) exhibit increasing neuroendocrine identity (+ to +++), with varying levels of AR signaling, primary cilium presence, mitochondrial activity, and luminal/basal marker expression. These features reflect the transcriptional and functional heterogeneity among neuroendocrine prostate cancer subpopulations.

UMAP analysis of all single-cells transcriptomes identified distinct cellular populations across conditions (Figure 7A). In samples from adjacent tissues (n = 3), cells distributed broadly across several clusters, reflecting the expected heterogeneity of non-malignant compartments (Figure 7B). Control tumors (n = 3) also contained a mixture of cell populations overlapping with those adjacent tissues but enriched in specific clusters associated with tumor cells. In contrast, NE tumors (n = 3) displayed a strikingly different population, with the majority of cells confined to a limited number of clusters, indicative of a distinct transcriptional identity and reduced intratumoral heterogeneity compared to control tumors. Notably, clusters 8 and 11 displayed a marked enrichment in the expression of neuroendocrine markers, suggesting the presence of a distinct neuroendocrine-like subpopulation within these cellular subsets (Figure 7C). To further clarify whether YAP1 expression occurs in the same cells that express NE markers, we examined YAP1-positive cells within these two clusters. In cluster 8 (n = 1,324), 7 cells expressed YAP1, and in cluster 11 (n = 482), 5 cells expressed YAP1. Within these rare YAP1-positive subsets, NE markers were detectable, although generally at low levels, indicating that YAP1 and NE gene expression are not mutually exclusive. These observations support the existence of mixed or transitional phenotypes rather than distinct, non-overlapping cell states (Figure S11). We then analyzed marker genes from clusters 8 and 11. Cluster 8 showed strong enrichment for primary cilium-related processes, including cilium organization, axoneme assembly, and microtubule-based movement, alongside chromatin remodeling and RNA processing. This suggests a transcriptionally dynamic population with active ciliogenesis (Figure 7D). Cluster 11 was enriched in purine metabolism, ATP biosynthesis, and synaptic vesicle-related pathways, pointing to a biosynthetically active and potentially secretory phenotype (Figure 7E). The presence of microtubule-based transport processes and cilium assembly also suggests involvement in cilium-associated functions, similar to Cluster 8. We analyzed the distinct signatures of the two clusters, further detailing their mitochondrial profiles, which revealed a decrease in mitochondrial activity (Figure 7F). These results were concomitant with induction of glycolysis (PFKFB3 and LDHA), a hypoxic adaptation (CA9) and a regulation of the lactate (MCT4) (Figure S12). The key characteristics of each cluster are summarized in Figure 7G.

Altogether, these results define transcriptionally distinct tumor subpopulations in which ciliogenesis emerges as a hallmark of NE differentiation, link to metabolic rewiring and biosynthetic activity that contribute to the functional heterogeneity of PCa.

## Discussion

While most cancers do not express PC, we previously described that the re-expression of primary cilia in a subset of patients was associated with a more aggressive clinical phenotype 23. We also proposed a simple molecular signature to detect cilia presence, based on the co-expression of *GLI1*^+^ and* IFT20*^+^. This led us to ask two key questions: (i) Could this mechanism represent a recurrent adaptative process in certain tumor contexts across cancers that initially lack primary cilia potentially leading to the development of more aggressive cell populations? (ii) Is this *GLI1*^+^/* IFT20*^+^ signature consistently observed in such contexts?

To address these two questions, we focused on PCa, which, despite its slow progression, exhibits multiple stages of resistance to different anti-cancer treatments available to patients. One of these stages is called neuroendocrine transdifferentiation 10, 49, often triggered by androgen therapies in combination with Androgen Receptor pathway inhibitors such as enzalutamide or abiraterone. While these treatments aim to block androgen signaling, they can inadvertently promote the emergence of neuroendocrine features, leading to more aggressive and treatment-resistant disease.

A previous study had already noted a reduction in the proportion and length of PC in prostatic intraepithelial neoplasia, invasive cancers, and perineural invasion lesions compared to normal tissues 19. Moreover, Zhang *et al.* showed that prostate cancer cell lines (LNCaP, 22Rv1, PC3) lacked PC. We confirmed this and extended the analysis to include DU145 and neuroendocrine NCI-H660 and MDA 114-13 cells (data not shown). Among these, only the NE cell lines displayed robust ciliation, with 60% and 15% of cells, respectively, exhibiting PC. These findings define a specific state in which cilia are re-expressed in PCa, which we were also able to confirm *in vivo* in CRPC patients compared to treatment-naïve individuals.

Taken together, these observations support the hypothesis that re-expression of PC may represent a recurrent adaptative feature observed in distinct tumor contexts. We have now observed this phenomenon in both renal cell carcinoma (RCC) and PCa, two distinct tumor types, where cilia presence is observed in association with increased aggressiveness and therapy resistance. This raises the possibility that acquisition of PC may accompany broader tumor cell plasticity program, potentially facilitating cilia-mediated signaling, transdifferentiation, metabolic reprogramming, or immune escape.

However, the GLI1⁺/IFT20⁺ signature was not as robust in prostate cancer as in ccRCC 23. While GLI1 remained a reliable marker, IFT20 was less consistently expressed. Another member of the IFT-B complex, IFT80, emerged as a potential substitute. Should we therefore broaden the signature to GLI1⁺/IFT20⁺/IFT80⁺ to improve detection across cancer types? Or should we retain GLI1 as a core marker and tailor the IFT component depending on tumor context? Systematic exploration of cancers typically lacking cilia will be necessary to define a comprehensive catalog of context-specific signatures.

To further investigate the heterogeneity of primary cilia expression in NE prostate cancer, we examined CRPC patient samples. Interestingly, in the four CRPC patients we observed a partial dissociation between NE differentiation and ciliogenesis: while some Pan-NE–positive cells displayed PC, others did not, and conversely, some ciliated cells were Pan-NE–negative. This was also observed in Patient 4 from the CAL cohort. These findings indicate that although PC can be present in NE cells, additional ciliated cell populations exist and remain to be characterized. The dissociation between Pan-NE expression and PC presence supports the view that these features arise at distinct stages of the transdifferentiation continuum.^50^. Moreover, this raises the possibility that once established within the tumor, PC could potentially participate in intercellular communication between cancerous and non-cancerous cells. In support of this, a seminal study in *C. elegans* demonstrated that ciliated sensory neurons can shed extracellular vesicles (ECVs) containing polycystin proteins into the environment, triggering specific behavioral responses in neighboring cells, thereby establishing a cilium-dependent communication system 51. Such cilia-associated signaling mechanisms have been proposed to the coordination of tumor plasticity, metabolic adaptation, or even therapeutic resistance. Exploring this hypothesis may open new avenues for understanding tumor heterogeneity and identifying novel intervention strategies.

To gain further insight into the identity of the ciliated cells observed in patient samples, we turned to single-cell transcriptomic data. We focused specifically on tumor-intrinsic processes by restricting our analysis (Dong/Song *et al.* 46, 47) to epithelial cells, excluding stromal, immune, and other non-epithelial populations. This refined analysis confirmed our immunofluorescence observations. Using a robust PC gene signature, we found that only the two clusters displaying a clear NE transcriptional identity also expressed the cilium signature. No such signature was detected in other epithelial clusters with luminal or intermediate features. These findings suggest that primary ciliogenesis is enriched within within NE-like epithelial clusters within the epithelial compartment of CRPC and may correspond to a distinct transcriptional and functional state. However, since non-epithelial populations were excluded from this analysis, we cannot rule out the presence of PC in other cell types within the tumor microenvironment, raising the possibility cilia-mediated interactions between cancerous and non-cancerous cells.

Recently, Pearson *et al.* proposed a pan-cancer classification based on YAP1 expression status 29, defining “YAP^on^” and “YAP^off^” tumors. YAP^off^ solid cancers, which include retinoblastoma, small-cell lung cancer, and NE prostate cancer, are typically neural or neuroendocrine in nature and often harbor *RB1* deficiency. Since YAP1 is a key component of the Hippo signaling pathway 52 and interacts with PC 30, 53, we hypothesized that YAP1 could link between the absence of PC expression in prostate adenocarcinomas to their re-expression in NE cancers. We confirmed that YAP1 is expressed across prostate adenocarcinoma cell lines, with levels correlating with tumor aggressiveness. In contrast, NE cell lines lacked YAP1 expression.

To explore the contribution of YAP1, we used two independent approaches to inhibit its function in prostate adenocarcinoma cells: the small molecule Jasplakinolide (Jasp.)**,** and YAP1-targeting siRNA/shRNA. Interestingly, only Jasp. treatment led to re-expression of PC, while genetic knockdown of YAP1 did not. This suggests that YAP1 repression alone is not sufficient to restore ciliogenesis. Instead, direct modulation of the actin cytoskeleton, as induced by Jasp., appears more effective. By stabilizing F-actin and reducing cortical tension, Jasp. appears to create a more permissive mechanical environment for ciliogenesis.

To further dissect the mechanisms involved, we compared RNA-seq data from cells treated with Jasp. *versus* shYAP1. Despite differences in ciliogenesis outcomes, both conditions induced overlapping transcriptional programs, including pathways involved in PC biogenesis. One intriguing observation was the association between cilia formation and mitochondrial downregulation. While seemingly counterintuitive, since ciliogenesis is energy-dependent, our findings are consistent with the possibility that ciliogenesis in this context is compatible with a predominantly glycolytic metabolic state rather than being strictly dependent on mitochondrial oxidative phosphorylation. This would be consistent with the glycolytic shift seen in NE cells. We further hypothesize that alternative energy sources, such as tryptamine metabolism, could support ciliogenesis in metabolically rewired cells. Derived from tryptophan, tryptamine can fuel glycolytic bypass routes and may provide an auxiliary energy supply in cells with compromised mitochondrial function. This concept is particularly relevant in the context of NED, where enhanced ciliogenesis coincides with suppressed oxidative metabolism.

Among PCa models, PC3 cells occupy a unique intermediate state. Initially classified as poorly differentiated adenocarcinoma, they have since been shown to exhibit features of small-cell neuroendocrine carcinoma 54, 55. These include the absence of AR and PSA, expression of neuroendocrine and *CD44* markers, and androgen independence. However, unlike classical neuroendocrine cells, PC3 cells express both YAP and TAZ, placing them at the intersection between adenocarcinoma and neuroendocrine phenotypes. This dual identity makes them a particularly interesting model to explore transitional states in ciliogenesis, metabolic rewiring, and lineage plasticity.

These mechanistic insights prompted us to consider the translational implications of our findings. Identifying new markers for NE prostate cancer is essential, as this aggressive and treatment-resistant subtype often arises under therapeutic pressure and lacks reliable diagnostic tools. Current markers do not adequately capture early transdifferentiation events or the heterogeneity of NE prostate cancer. Our findings suggest that the presence of the PC, along with a distinct glycolytic shift, characterizes specific cell populations with NE features. These two signatures, structural and metabolic, may represent candidate functional biomarkers that warrant further validation to detect early lineage reprogramming and identify tumors likely to escape androgen receptor–targeted therapies. Their combined use could improve diagnostic precision, enable better patient stratification, and open new therapeutic avenues targeting cilia-related signaling and metabolic dependencies. Beyond their diagnostic relevance, our results identify the PC as a potentially modulable cellular structure in experimental settings. We demonstrated that ciliogenesis can be induced in adenocarcinoma cells or suppressed in NE models, offering new experimental and therapeutic strategies to modulate tumor plasticity.

Building on these insights, our findings provide a conceptual framework for exploring therapeutic strategies targeting cilia-dependent plasticity. For instance, a compound like HPI-4could be used in combination with castration therapies to block neuroendocrine transdifferentiation and prolong patient response. Although HPI-4 remains the only known compound that disrupts ciliogenesis, its short-term cytotoxicity limits clinical use. Nonetheless, our findings show that PC correlate with high metastatic burden and a NE phenotype, which can be identified through FDG-PET imaging, reflecting their enhanced glycolytic metabolism. More systematic use of FDG-PET in patients with aggressive prostate tumors could improve early detection and therapeutic stratification, as previously suggested by Jadvar 56. This opens promising avenues for future investigation into drug repositioning, including the use of Hedgehog pathway inhibitors (e.g., vismodegib 57, sonidegib, or even GLI inhibitors like GANT61 58) to indirectly target the cilium-dependent plasticity axis. The development or repurposing of less toxic agents interfering with ciliogenesis may inform the future design of personalized therapeutic approaches, selectively administered to patients with extensive, ciliated, and NE-like prostate tumors. Such biomarker-driven approaches would mark a significant advance in the treatment of aggressive PCa.

## Methods

### Cell culture

The P69 cell line was derived by immortalization of human primary prostate epithelial cells with simian virus-40 T antigen and was grown in RPMI 1640 with 10% FBS. The normal human cell line RWPE-1 originated from non-neoplastic human prostate epithelial cells, immortalized with human papillomavirus. The RWPE-1 cell line was cultured in Keratinocyte-Serum Free (K-SFM) medium supplemented with Epidermal Growth Factor (EGF) and Bovine Pituitary Extract (BPE). LNCaP, DU145, and PC3 cells were purchased from the ATCC. Upon reception, cells are thawed at low passages. All cells used in this study were within 20 passages after thawing and tested monthly for *Mycoplasma*. DU145 and PC3 cells were cultured in DMEM (Gibco). LNCaP cells were cultured in RPMI1640 medium (Gibco). Cell media were supplemented with 10% FBS (Gibco) and 1% penicillin/streptomycin (100 U/mL and 100 μg/mL, respectively; Gibco) and incubated at 37 °C and 5% CO_2_. LNCaP-shCtl, LNCaP-sh*YAP1* were cultured in RPMI1640 medium (Gibco) and DU145-shCtl and DU145-sh*YAP1* were cultured in DMEM (Gibco) with both 10% FBS and 1µg/ml of puromycin 38.

An INVIVO_2_ 200 anaerobic workstation (Ruskinn Technology Biotrace International Plc) set at 1% oxygen, 94% nitrogen and 5% carbon dioxide were used for hypoxic conditions.

### Patient cohort

- Henri Mondor Hospital

Prostate tissue samples were collected as part of an Institutional Review Board approved protocol at Henri Mondor Hospital in France (CPP no. 16169). In this cohort, 7 PCa tissue samples were collected, including 5 samples from radical prostatectomy of patients that didn’t receive prior hormone treatment at the hospital (Localized PCa, Hormone-naïve Prostate Cancer, HNPC) and 3 tissues collected by transurethral resection from Castrate-Resistant Prostate Cancer (CRPC) patients. The study was conducted in accordance with the guidelines in the Declaration of Helsinki and the use of all patient tissue specimens was carried out according to French laws and regulations.

- Antoine Lacassagne Center (CAL)

Patients who underwent metastatic biopsy at the Antoine Lacassagne Center and had a PET-FDG examination performed as part of routine clinical care were retrospectively identified using a keyword-based search of the institutional database. Clinical and demographic data were extracted from medical records. Standardized uptake values (SUVs) from PET-FDG scans were reviewed and analyzed by the Department of Nuclear Medicine. Representative PET-FDG images were captured with the Snipping Tool and archived for illustrative purposes.

### Pharmacological inhibitors and chemicals

Cells were incubated with 1- and 10 µM of Gefitinib (Gef.) from Sigma Aldrich (France), with 100 μΜ of Clofibrate from Sigma Aldrich (France), with 1μM of Jasplakinolide (Jasp.) from AdipoGen AG (Switzerland) and 120 μM of HPI-4 from Sigma Aldrich (France).

### Cell counting for viability and proliferation assessment

Cells were plated at 100,000 cells/well and treated the following day. At specific times, cells were detached using trypsin-EDTA, suspended in their conditioned medium and evaluated for viability and proliferation using an automatic cell counter (Advanced Detection Accurate Measurement system, Digital bio, NanoEnTek Inc., Seoul, Korea).

Cell viability was quantitatively assessed specifically under the HPI-4 treatment conditions at each time point using an automated cell counter, confirming stable viability (~80%) for the selected 12 h exposure.

Importantly, all downstream experiments (including metabolic measurements) were performed exclusively on live cells under these conditions, and viability was systematically monitored in parallel.

### Respirometry

The cellular oxygen consumption rate (OCR) and extracellular acidification rate (ECAR) were obtained using a Seahorse XF96 extracellular flux analyzer from Seahorse Bioscience (North Billerica, MA, USA) following the manufacturer’s instructions. OCR and ECAR were measured in real time in Nx, Phx, or Hx. Cells were deprived of glucose for 1h, then glucose (G–10mM), oligomycin (O–1µM), 2,4-Dinitrophenol (DNP–10µM), and Rotenone + Antimycin A (R/A–1µM) were injected at the indicated times. OCR values were normalized to protein content after each experiment. No significant differences in protein concentration or cell phenotype were observed between conditions. Cell viability was assessed in parallel under the same experimental conditions.

### OmniLog metabolic assay

Prior to seeding, cell viability was assessed using an automated cell counter, and only cell suspensions with > 90% viability (P3) or ~80% viability (NCI-H660, HPI-4 conditions) were used to ensure that OmniLog readouts reflected metabolic activity rather than differences in cell survival. The assay was performed as described by Biolog Inc. (OmniLog® Phenotype MicroArray System. Biolog, Hayward, CA, USA). Briefly, P3 and NCI-H660 cells were harvested and washed twice with Biolog Inoculating Fluid IF-M1 (Biolog) to remove residual culture medium. A suspension of 40,000 cells in 50 µl IF-M1 was seeded into each well of 96-well PM-M1 plates. For the MitoPlate S-1 assay, cells were permeabilized in MAS buffer (Biolog, cat. 72303) containing saponin (50 µg/mL) and Redox Dye Mix MC (Biolog, cat. 74353). Plates (MitoPlate™ S-1, Biolog) were pre-activated with 30 µL MAS per well for 1 h at 37 °C, then seeded with 2–4 × 10⁴ cells in 30 µL per well.

Kinetics of dye reduction were recorded in the OmniLog PM-M at 37 °C every 15 min for 24 h (A590–A750). Blank wells (no cells) were used for background subtraction; signals were summarized as initial rates and AUC, following the manufacturer’s instructions.

### Migration

The migration assay was performed using cell culture inserts with 8µm pore transparent PET membrane (ref: 353097, CORNING-FALCON). Inserts were incubated in medium without FBS for 1 h at 37°C in a CO_2_ incubator. Overnight serum-starved cells (6×10^5^ cells) were seeded into the top chamber in medium without FBS, while medium with 10% FBS and 10μg/ml Fibronectin was present in the bottom chamber (without FBS for NCI-H660). The cells were incubated for 6h. Media and remaining cells were removed from the top chamber with a cotton swab and washed twice with PBS. Inserts were fixed with 4% PFA. Cells that migrated through the filter and adhered to the lower surface of inserts were stained for 30min with 0.5% crystal violet in PBS. Inserts were rinsed in distilled water until no additional stain leached and were air-dried overnight. Cells staining was captured by EVOS. Cell numbers were manually counted.

### Transfection and siRNA

Cells were transfected with the 21-nucleotide RNAs control (siCtl) chemically synthesized (Eurogentec, Seraing, Belgium) and previously described 59 and the siRNA sequence was as follows: siCtl (forward) 5’-CCU-ACA-UCC-CGA-UCG-AUG-AUG-TT-3’. siYAP1 #1 (ID:107951) from ThermoFisher Scientific (France), or the siYAP1 #2 (sc38637) Santa-Cruz Biotechnology (Germany). Cell viability was systematically assessed by nuclear morphology in immunofluorescence assays and Trypan Blue exclusion was used for complementarity viability assessment in cell-based experiments.

### qPCR

Total RNA was extracted with the RNeasy Mini Kit (QIAGEN, Hilden, Germany). The amount of RNA was evaluated with a NanoDrop™ spectrophotometer (ThermoFisher Scientific, Waltham, MA USA). One μg of total RNA was used for reverse transcription, using the QuantiTect Reverse Transcription kit (QIAGEN, Hilden, Germany), with oligo (dT)_15_ to prime first-strand synthesis. SYBR master mix plus (Eurogentec, Liege, Belgium) and specific oligonucleotides (Sigma Aldrich) were used for qPCR. Primer sequences used were: *GLI1* (forward) 5'-TGCAGTAAAGCCTTCAGCAATG -3' and (reverse) 5'-TTTTCGCAGCGAGCTAGGAT- 3'; *IFT20*** (**forward) 5'-GGTATCGGGTTGAATATGAAG-3' and (reverse) 5'-GACATAGGTCATTGGTCAAG-3'.

### Immunoblotting

Cells were lysed in 1.5x SDS buffer and the protein concentration determined using the BCA assay. 40 µg of protein from whole cell extracts was resolved by SDS-PAGE and transferred onto a PVDF membrane (Millipore). Membranes were blocked in 5% non-fat milk in TN buffer (50 mM Tris-HCl pH 7.4, 150 mM NaCl) and incubated in the presence of the primary and then secondary antibodies in 5% non-fat milk in TN buffer. Mouse monoclonal anti YAP1 (sc-101199) and anti ERK2 (sc-1647) antibodies were from Santa Cruz Biotechnology (Germany). Mouse monoclonal anti acetylated tubulin (T7451) was from Cell Signaling (France). ECL signals were normalized to ERK2. After washing in TN buffer containing 1% Triton-X100 and then in TN buffer, immunoreactive bands were visualized with the ECL system (Amersham Biosciences).

### Immunofluorescence on cells

Cells were seeded in imaging-specific plates 24-well (P24-1.5P) from Cellvis (USA). They were then washed with 1X PBS and fixed with 4% paraformaldehyde (PFA) for 20min, followed by three washes with 1X PBS. Cells were permeabilized for 5min in 0.2% PBS/Triton X-100, washed with PBS, and blocked for 30min with PGB buffer (PBS-Gelatin-BSA). They were then incubated overnight at 4°C with primary antibodies: polyclonal rabbit anti Arl13B antibody from Novus Biologicals (France), monoclonal mouse anti Arl13b antibody from Santa Cruz Biotechnology (Germany), monoclonal mouse anti acetylated α-tubulin antibody from Sigma Aldrich (France), polyclonal rabbit Pericentrin antibody from Bethyl Laboratoires from ThermoFisher (France), and mouse monoclonal anti YAP1 (sc-101199) antibody from Santa Cruz Biotechnology (Germany). After three washes with 1X PBS, cells are incubated in the dark for 1h at room temperature with a secondary antibody conjugated with a fluorophore and DAPI. Plates are then washed three times and stored in PBS at 4 °C before being observed under a NIKON A1R® confocal microscope.

### Immunofluorescence on biopsies

5 μm of FFPE tissue sections were deparaffinized and Heat Induced Epitope Retrieval (HIER) with Epredia™ Dewax and HIER Buffer H. The tissue sections and buffer were placed on the rotating plate in a microwave oven and heated at 900 W for 6 min boiling vigorously followed by 10 min at 350W, resulting in gentle pulsatile boiling. Hereafter the sections were left cooling for 30 min in fresh buffer at RT. Endogenous peroxidase in tissues was blocked with BLOXALL Blocking Solution (Vector Laboratories) for 10 min. Sections were washed 3 times in Tris-buffered saline–0.05% Tween20 (TBST) and blocked with Antibody Diluent (Zytomed-Systems) for 30 min, before incubation with a primary antibody for 60 min. Antibodies were diluted in Antibody Diluent. For multiplex immunofluorescence staining, the sections were incubated in three rounds of staining; in the order of mouse anti-acetylated α-tubulin (Sigma-Aldrich, Basel, Switzerland; 1:500 dilution); rabbit anti-Arl13b (Novusbio, Abingdon, United Kingdom; 1:200 dilution) and mouse anti-TUBB3 (Abcam, Cambridge, UK; 1:200 dilution), Next, tissue sections were washed and incubated for 30 min with horseradish peroxidase (HRP)-conjugated anti-mouse or anti-rabbit polymer (POLYVIEW^®^ PLUS HRP reagent, Enzo life sciences, Lyon, France). Epitope–antibody binding was visualized using separate Opal fluorophore (Opal570, Opal520, Opal650, Akoya Biosciences, Marlborough, MA) according to the manufacturer’s protocol. Tris based antigen unmasking solution (Vector laboratories, Eurobio scientific, Les Ulis, France) was used in between rounds of Opal signal amplification to remove the antibody from the previous round, to avoid any cross-reactivity. Briefly, the antigen unmasking solution was boil in a microwave oven and heated at 900 W for 5 min boiling vigorously followed by 10 min at 350W. Finally, after staining with the three Opal fluorophore, tissue sections were stained with 4′,6-diamidino-2-phenylindole (DAPI) for 5 min and mounted in ProLong Diamond Antifade Mountant (ThermoFisher Scientific).

### Electron microscopy

Cells were fixed using 1.6% glutaraldehyde in 0.1 M phosphate buffer at room temperature (RT) and subsequently at 4 °C for 16 h. After washing with the same buffer, samples were processed for 1h at RT with 1% osmium tetroxide and 1% potassium ferrocyanide in 0.1 M cacodylate buffer. Cells were then washed with distilled water, coated in epoxy resin and prepared for thin sectioning in the conventional way. These sections were observed using a JEM1400 transmission electron microscope (Jeol) with Morada CCD camera (Olympus SIS).

### Big data analysis

**cBioPortal** (http://www.cbioportal.org/index.do).

The database consists of the TCGA of cancer genomic data. We have analyzed the datasets from the TCGA containing 550 adenocarcinoma PCa. Clinical information for all 550 patients.

### Public RNA-seq dataset

RNA-seq data from GEO accession GSE197780 45 were downloaded and processed using the same quality control and normalization pipeline as our in-house datasets. This dataset contains transcriptomic profiles of primary prostate tumors before and after neoadjuvant enzalutamide treatment and was used to validate the transcriptional changes observed in our study.

### RNASeq analysis

The data were analyzed using different platforms. Venn diagram for differentially expressed genes was performed on Bioinformatics and Evolutionary Genomics (Draw Venn Diagram (ugent.be)). Phantasus v1.11.0 (https://artyomovlab.wustl.edu/phantasus) was used for drawing heatmap and performing principal component analysis, ShinyGo 0.76 (ShinyGO 0.76 (sdstate.edu)) was used for molecular component and molecular function analysis.

### Single-cell analysis 47

Data of single cell experiments of prostate cancer and normal prostate tissues from datasets gse137829 47 and gse176031 46 were used for analysis. For gse176031, patients P7N2, P8N2, P10N1, P8T1, P9T1 and P10T2 were used. Raw data were pre-processed with using 10x Genomics Cell Ranger 8.01 software, with default parameters to perform alignment, filtering, barcode counting, and unique molecular identifier (UMI) counting. Reads were aligned on the GRCh38 reference genome. Analysis of scRNA-seq data was conducted using the Seurat package (V5.02) by R programming. Quality control (QC) was performed to remove doublets and low-quality cells based on the number of unique genes and the proportion of transcripts corresponding to mitochondrial RNA and ribosomal genes. After filtration, gene counts were normalized using the NormalizeData function of Seurat R package (logNormalize method and scale factor of 10,000). We centered the expression data from these factors using the Seurat R package ScaleData function (centering true and scaling false). Dimensionality reduction and clustering were performed using UMAP embedding map colored by Seurat clustering. Clusters of cells were identified with Louvain algorithm provided by Seurat method FindClusters. Marker genes between clusters were identified using the FindAllMarkers method of the Seurat package using the Wilcoxon Rank Sum test on genes expressed at least in 10% of the cells, a logFC threshold of 0.25 and a FDR threshold of 0.001. Gene Ontology analysis was performed using ClusterProfiler package (v.4.14.6).

For each sample, epithelial cells were subset based on PCAM, KRT8, KRT5 and CDH1 markers. These subsets were then merged for a complete analysis of all patients at the same time.

### Statistics

All values are the means±SEM. Statistical analyses were performed using unpaired two-tailed t-tests, ordinary one-way ANOVA and two-way ANOVA, as appropriate, using GraphPad Prism 9 software. The specific statistical test used is indicated in each figure legend. All categorical data used numbers and percentages. All categorical data are expressed as numbers and percentages. Statistical analysis: Anova; significant differences are indicated by * *p* < 0.05, ** *p* < 0.005, *** *p* < 0.0005, and **** *p* < 0.0001.

## Supplementary Material

Supplementary figures.

## Figures and Tables

**Table 1:** ** Patient characteristics at time of metastatic biopsy.** *NA: Not Available*, *ADT: Androgen Deprivation Therapy*, *AA: Abiraterone Acetate*, *CDDP: Cisplatin, GEMCI: Gemcitabine*, *CARBO: Carboplatin*, *VP16: Etoposide*, *Syn: Synaptophysin*, *ChgA: Chromogranine A*, *NSE: Neuron-specific enolase.* ^1^Age at biopsy (years), 2Gleason score at the first prostate biopsy or prostatectomy, ^3^PSA level at the time of the biopsy (ng/mL), ^4^Localisation of visceral metastases at metastatic biopsy, ^5^Number of hormone therapy lines before biopsy, 6Number of chemotherapy lines before biopsy, ^7^NE marker staining of the biopsy.

Patient No.	Age^1^	Gleason score^2^	PSA at biopsy^3^	Visceral metastasis^4^	No. of HT lines^5^	No. of chemotherapy line^6^	NE marker staining^7^
1	60	NA	651	No	1) AA 2) Enzalutamide	1) Docetaxel 2) Cabazitaxel 3) Mitoxantrone 4) Navelbine	No
2	66	8 (4+4)	< 0.04	Liver and Lung	1) ADT alone	1) CARBO-VP16	Yes
3	73	7 (3+4)	53	Liver	1) AA 2) Enzalutamide	1) Docetaxel 2) Cabazitaxel 3) Mitoxantrone 4) PSMA lutetium	Yes
4	67	9 (5+4)	94	No	1) ADT alone	None	Yes
5	73	NA	2179	No	None	None	Yes

**Table 2:** Biopsy characteristics and Standard Uptake Value (SUV).

					Total lesion volume		Most intense lesion			Biopsied lesion			
Patient No.	Usable paraffin-embedded tissue sample	Biopsy date	PET-FDG date	Local biopsy	SUVmean	Total metabolic volume (ml)	SUVmax (Most intense)	SUV pic	Location (if different)	SUVmax (Biopsied)	SUVmean (Biopsied)	Biopsied location	Number of lesions
1	+	23/04/2015	24/06/2015	Left external iliac lymph node	FDG-negative lesion	FDG-negative lesion	2.39	1.8	—	2.21	1.67	Left external iliac	Do not uptake FDG
2	+	29/07/2020	08/07/2020	Liver	Not Known	Not Known	37.23	27.41	Left para-aortic	16.25	7.51	Liver	> 50
3	+	01/03/2023	15/07/2022	Liver	3.22	210	15.52	10.39	—	15.52	6.38	Liver (central)	around 20
4	+	30/08/2013	30/08/2013	Left subclavian lymph node	8.67	78	10.04	6.38	Right superior mediastinal	6.73	4.61	Left supra clavicular	around 15
5	+	17/06/2021	21/05/2021	Left retroperitoneal lymph node	4.1	1124	11.44	7.35	—	11.44	4.01	Left retroperitoneal	> 50

**Table 3:** Stratification of patients based on lesion burden, FDG Uptake, primary cilium presence, and neuroendocrine features.

Group	Characteristics	No. of Hormone Therapy lines	No. of chemotherapy line	Interpretation	Patient Examples
Signature 1 (Low aggressiveness)	- Very low or no detectable lesions - No primary cilium - Little or no FDG uptake	2	4	Less aggressive phenotype, low metabolic activity, possibly still differentiated	Patient 1
Signature 2 (High aggressiveness)	- > 50 lesions - Primary cilium present - High metabolic engagement, but SUV not discriminatory - NE+ markers (Pan-NE+)	0 to 2	0 to 4	Highly aggressive phenotype, possibly proliferative and neuroendocrine-stabilized	Patients 2, 3, 4 & 5

**Table 4:** List of the up-regulated genes from Cluster 1 obtained by comparing DU145-shCtl cells, DU-shYAP1 and DU145-shCtl treated with 1µM of Jasplakinolide (Jasp.).

nGenes	Pathway Genes	Fold Enrichment	Pathway	Genes
33	464	1.989	Microtubule	MARK4, HOOK2, DNAL4, TTLL1, NINL, TUBB1, SNPH, NDRG1, CLIP3, DNM1, LZTS2, DNAH1, ARHGEF2, KIF21B, EML2, MAP1B, TPT1, APC, FHDC1, TRIM54, KIFC3, CSNK1D, BCL2L11, PBXIP1, REEP3, GOLGA2, KIFC2, HID1, RGS14, GABARAP, SVIL, KIF13B, TTLL3
44	696	1.768	Cilium	MARK4, RABL2B, TNPO1, RPGRIP1, WHRN, CATSPERG, DNAL4, TTLL1, CACNA1F, PDE4C, IQCE, AMBRA1, GUCA1B, DNAH1, CEP104, BBOF1, BBS2, NPHP4, MAP1B, ARMC9, RNF38, FHDC1, FAM149B1, AK7, CCDC40, CSNK1D, PRKACB, GPR161, PIP4K2A, DNAI4, LCA5L, CFAP251, SQSTM1, DHRS3, MXRA8, GABARAP, CFAP53, CATSPERE, DNHD1, IFT140, RILPL1, CFAP43, CYS1, TTLL3

**Table 5:** List of the up-regulated genes from Cluster 2 obtained by comparing DU145-shCtl cells, DU-shYAP1 and DU145-shCtl treated with 1 µM of Jasplakinolide (Jasp.).

nGenes	Pathway Genes	Fold Enrichment	Pathway	Genes
3	5	29.414	Cortical microtubule cytoskeleton	CLASP1, NUMA1, PDE4DIP
28	658	2.086	Centrosome	SPPL2B, PPP2R5A, FBXW11, CLASP1, IL4R, KEAP1, STX1B, SNAP29, MIB1, ARL2BP, CDK5RAP3, NEK11, ODF2L, PATJ, DYSF, CEP350, KIF13A, NUMA1, NLRC5, SERINC5, CCDC88B, BBS1, PDE4DIP, PLA2G6, PDE4B, TMEM63A, UVRAG, DYNLT2B
34	868	1.920	Microtubule organizing center	SPPL2B, SPATA7, PPP2R5A, FBXW11, CLASP1, IL4R, KEAP1, STX1B, SNAP29, MIB1, ARL2BP, CDK5RAP3, NEK11, ODF2L, PATJ, DYSF, CEP350, KIF13A, NUMA1, NLRC5, CFAP410, SERINC5, CCDC88B, BBS1, PDE4DIP, PLA2G6, PDE4B, DYNLT4, TMEM63A, UVRAG, FANK1, TTC23L, DYNLT2B, CFAP206

**Table 6:** List of the down-regulated genes from Cluster 3 obtained by comparing DU145-shCtl cells, DU-shYAP1 and DU145-shCtl treated with 1µM of Jasplakinolide (Jasp.).

nGenes	Pathway Genes	Fold Enrichment	Pathway	Genes
128	1489	2.851	Nuclear protein-containing complex	POLR3B, NUP160, SNRNP40, TAF2, WDR18, MCM10, POLR1A, CLNS1A, TIPIN, NUP37, ORC1, SUPT16H, CDC45, NUP188, CWF19L1, HNRNPM, RANBP1, SNRPD3, SNU13, PHF5A, E2F1, CSTF2, SUPT20H, NUP93, POP1, INTS10, POLD2, NUP88, GAR1, NUP107, CHD4, NUP155, HDAC1, CDC20, EXOSC8, EXOSC9, MPHOSPH10, NUP153, NUP85, SNRPB2, XRCC3, XPO7, EXOSC2, DKC1, GINS2, SNRPA1, NUP210, CCNH, CDK4, IMP4, TEX10, PPIL1, RTF1, BARD1, NUP54, WDR61, SAE1, SNRPG, NHP2, ZMAT2, SSRP1, DSN1, ANAPC1, BUB3, TSEN2, NUP205, MED27, MAGOH, PBRM1, TERT, TRA2A, ORC5, INTS6L, POP5, EXOSC10, POLE, SF3A3, TAF9B, FANCA, GTF2F2, NOC2L, RRP7A, HDAC2, BRD9, PPIH, GCFC2, RAD51, YBX1, EPB41L2, DYNLL1, HSP90AB1, PRMT5, SUV39H1, ERCC2, EZH2, BCCIP, UBE2S, NUP98, RAD23B, RPAP2, MYBBP1A, SYNCRIP, WRAP53, PIP5K1A, MMS22L, SENP3, MAD2L1, STOML2, LEO1, UBE2C, TEAD1, DDX20, MYBL2, CEBPZ, TTF2, RBM17, API5, NOLC1, POP7, FMC1-LUC7L2, BIRC5, BUB1B, MCM7, HNRNPAB, HNRNPH3, RAN, IPO5, SNUPN
111	1695	2.172	Catalytic complex	NDUFAB1, POLR3B, PSMA4, SNRNP40, TAF2, POLR1A, RAD18, PIGS, DYNLL1, DLD, SNRPD3, KCTD17, PSMC6, PSMC1, PIGU, PRPS2, SUPT20H, POP1, TUSC3, POLD2, PSMD11, NDUFC1, GAR1, CHD4, CCND3, HDAC1, CDC20, RPN2, EXOSC8, CDK2, EXOSC9, PIGT, SPCS3, EXOSC2, DKC1, CCNH, TEX10, PSMB7, PPIL1, DCUN1D5, RTF1, BARD1, PPCDC, WDR61, SAE1, SNRPG, CCNA2, SKP2, NHP2, PDSS1, PFKM, ANAPC1, TSEN2, CCNB2, MAGOH, PSMD6, PBRM1, TERT, PSMC3, POP5, EXOSC10, NAA20, CCNE2, POLE, TAF9B, GTF2F2, PIK3R4, HDAC2, BRD9, RAD51, CLNS1A, HNRNPM, PRMT5, ERCC2, EZH2, BCCIP, UBE2S, RPAP2, SNRPB2, SNRPA1, MYBBP1A, STT3A, SYNCRIP, CDK4, PNPT1, SENP3, VCP, KBTBD6, LEO1, POP7, UBE2C, ATAD5, UBE2N, SF3A3, P4HB, MTARC1, UQCC3, RAD23B, PSME3, PRKAA1, UBQLN1, GMPR, MED27, NOLC1, RRM2, RIOK1, IFIT5, GLMN, BCKDK, BCS1L, BUB1B
109	1126	3.210	Nucleolus	RPL7, NOP16, MRTO4, POLR1A, TCOF1, NLE1, PUM3, DDX18, POP1, NPM3, GTPBP4, FTSJ3, GAR1, CCDC86, BYSL, BRIX1, WDR75, PNO1, RPF1, EBNA1BP2, RCL1, UTP20, MPHOSPH10, TTF1, KRI1, NGDN, DKC1, MYBBP1A, NIP7, GNL2, DDX56, IMP4, NUSAP1, URB1, LYAR, NHP2, UTP14A, WDR43, NOLC1, POP5, EXOSC10, NPM1, NOC2L, RRP7A, EXOSC8, EXOSC9, EXOSC2, GCFC2, NOP58, MCM10, TIMM13, SNU13, VRK1, SUV39H1, GRWD1, UBTF, MACROH2A1, RRP9, SPTBN1, RPAP2, MRM2, NUP153, SNRPB2, PIMREG, ILF3, CEP85, HABP4, RAN, CDCA8, CDK4, PWP1, SMC2, SKP2, MKI67, BTBD10, DSN1, RRP1B, MED27, SENP3, NOL9, TERT, TRA2A, CDCA7L, LEO1, FEN1, XPO6, PA2G4, POP7, UBE2N, JPT1, BLM, PRMT6, HAUS7, DDX47, IPO5, WDR18, HNRNPM, KNOP1, VPS29, TEX10, RTF1, MYG1, PLK4, RPL7L1, SSRP1, TSEN2, TSR1, LRRC34, CENPW
100	1830	1.812	Mitochondrion	CS, PKM, DLD, HSP90AB1, BCKDK, GOT2, PTGES2, VDAC2, PHB1, CYCS, MAOA, VDAC1, NDUFAB1, ELAC2, TOMM34, BAK1, ALDH18A1, KARS1, BCS1L, OXCT1, TIMM13, GSR, CHCHD2, COA1, MTPAP, NDUFC1, GRPEL1, MRPS30, MRPL3, MRPL37, MRM2, FASTKD3, SLC25A19, MGME1, ROMO1, ECHS1, TOMM40, GTPBP3, MTHFS, MRPL47, PNPT1, ABHD10, SFXN2, GTPBP8, SFXN1, PRDX3, DTYMK, PRELID1, HARS1, TMEM126A, COQ2, MRPL48, TMEM70, COX5A, TXNRD2, ATAD3A, UQCC3, LYRM4, MTFP1, NFS1, MRPL4, RAD51, OAT, GCDH, NUDT1, LRRC59, XRCC3, ILF3, NGDN, TEX10, ALDH1B1, MYG1, TMEM177, ABCE1, TERT, STOML2, FEN1, LETM1, UCP2, DCTPP1, MTARC1, ARHGAP11B, CYP24A1, TFRC, DDX1, DYNLL1, ACOT7, DUT, STARD13, NMT1, SORD, PIF1, SPATA5, PDSS1, NRGN, CDC25C, METTL17, TMX2, TUSC3, ADAP2
97	1441	2.232	Microtubule cytoskeleton	SPDL1, WDR62, KIF22, TPX2, DYNLL1, BIRC5, RANBP1, IFT52, MAPRE1, FAM83D, CEP152, KIF20A, CNTRL, KIF18A, TUBA1B, STIL, AUNIP, KNSTRN, MAP7D3, CRACR2A, CEP85, HAUS8, CDCA8, ESPL1, ODF2, NUSAP1, KIF23, PLK4, KIF2C, CEP78, INCENP, SCLT1, CCNB2, PLK1, TUBA1C, C2CD3, KIF5B, CCDC96, CCNE2, AURKB, NPM1, TUBB4B, PRC1, HAUS7, ZWILCH, MKS1, TACC3, RAD18, SPAG5, RIF1, NUDC, CDC45, CDC6, ATP6V1D, E2F1, RAB11A, ERCC2, BCCIP, CHD4, CCT4, CDK2, RAN, CCT7, DCUN1D5, PKP4, SRPRB, SPATA5, CCT6A, CCT5, CENPU, TSEN2, BUB1B, RACGAP1, MAD2L1, CCT2, LEO1, PCLAF, RAB8A, HASPIN, GTF2F2, H2AX, RRP7A, PIK3R4, SPTAN1, HYPK, CCDC124, RAD51, KIF4A, TTLL12, NUP93, TXNDC9, CDC20, NUP85, AJUBA, TEDC2, GAPDH
86	1380	2.067	Organelle envelope	LMNB1, NDUFAB1, NUP160, BAK1, BCS1L, NUP37, NUP188, TIMM13, RANBP1, NUP93, COA1, NUP88, NDUFC1, GRPEL1, NUP107, NUP155, CSE1L, NUP153, NUP85, SLC25A19, ROMO1, TOMM40, XPO7, NUP210, NUP54, NUP205, SFXN2, SFXN1, VDAC2, PRELID1, CYCS, COQ2, TMEM70, COX5A, UQCC3, VDAC1, MRPL4, MRPS30, MRPL3, MRPL37, MRPL47, MRPL48, TOMM34, TNPO3, RIF1, CHCHD2, GTPBP4, NUP98, GAPDH, HABP4, RAN, CDK4, SMPD4, PNPT1, TMEM177, MINDY3, MAD2L1, STOML2, PHB1, LETM1, LMNB2, JPT1, ATAD3A, ITSN1, MTFP1, ALDH18A1, RTCB, NUDT1, LRRC59, TMEM109, STARD13, MNS1, SORD, EI24, NRGN, CDC25C, TMEM126A, BANF1, UCP2, MTARC1, MAOA, TMX2, ADAP2, CYP24A1, IPO5, SNUPN
86	1380	2.067	Envelope	LMNB1, NDUFAB1, NUP160, BAK1, BCS1L, NUP37, NUP188, TIMM13, RANBP1, NUP93, COA1, NUP88, NDUFC1, GRPEL1, NUP107, NUP155, CSE1L, NUP153, NUP85, SLC25A19, ROMO1, TOMM40, XPO7, NUP210, NUP54, NUP205, SFXN2, SFXN1, VDAC2, PRELID1, CYCS, COQ2, TMEM70, COX5A, UQCC3, VDAC1, MRPL4, MRPS30, MRPL3, MRPL37, MRPL47, MRPL48, TOMM34, TNPO3, RIF1, CHCHD2, GTPBP4, NUP98, GAPDH, HABP4, RAN, CDK4, SMPD4, PNPT1, TMEM177, MINDY3, MAD2L1, STOML2, PHB1, LETM1, LMNB2, JPT1, ATAD3A, ITSN1, MTFP1, ALDH18A1, RTCB, NUDT1, LRRC59, TMEM109, STARD13, MNS1, SORD, EI24, NRGN, CDC25C, TMEM126A, BANF1, UCP2, MTARC1, MAOA, TMX2, ADAP2, CYP24A1, IPO5, SNUPN
85	1513	1.863	Supramolecular complex	SPDL1, KIF22, TPX2, BIRC5, MAPRE1, COTL1, KIF20A, KIF18A, TUBA1B, FASTKD3, AIF1L, KNSTRN, AJUBA, HAUS8, KIF23, CENPO, DIAPH3, KIF2C, SNRPG, INCENP, DSN1, BUB3, MAD2L1, TUBA1C, KIF5B, ZWILCH, CSRP2, AURKB, APOBEC3B, TUBB4B, CNOT7, CENPW, KNL1, PBRM1, CLDN11, NUP160, YBX1, NUP37, SPAG5, FBLN1, DDX1, DYNLL1, RPL6, CORO1A, RAB11A, NUP98, NUP107, CENPA, CCT4, FKBP1B, NUP85, SNRPB2, HABP4, TRIM5, ANXA1, CCT7, EIF4E2, BARD1, SRPRB, CCT6A, CCT5, BUB1B, VCP, CCT2, PLK1, HAUS7, EID1, PSMA4, NUDC, KIF4A, CENPM, LMNB1, EIF4G1, SPTBN1, EIF2S1, ODF2, NUSAP1, MNS1, CENPU, RACGAP1, CENPN, LMNB2, HNRNPAB, PRC1, PSMC3
79	807	3.246	Ribonucleoprotein complex	RPL6, RPL7, MRTO4, NOP58, SNRNP40, YBX1, CLNS1A, CWF19L1, HNRNPH3, HNRNPM, SNRPD3, SNU13, PHF5A, POP1, FTSJ3, GAR1, BYSL, MRPS30, MRPL3, RRP9, RPF1, EBNA1BP2, UTP20, MPHOSPH10, RIOK1, SNRPB2, KRI1, NGDN, RPS4Y1, DKC1, SNRPA1, NIP7, EIF2S1, SYNCRIP, MRPL47, IMP4, PPIL1, NOB1, SNRPG, NHP2, ZMAT2, RPL7L1, UTP14A, RRP1B, MAGOH, RPL39L, TERT, TRA2A, POP5, TSR1, NPM1, SF3A3, NOC2L, RRP7A, RBM12, MRPL4, MRPL37, PPIH, MRPL48, NDUFAB1, GCFC2, DDX1, SRRT, GAPDH, ILF3, HNRNPD, WRAP53, POP7, HNRNPAB, TTF2, RBM17, PNPT1, EIF3M, API5, NOLC1, EIF3C, FMC1-LUC7L2, NUP98, EIF4G1
63	888	2.353	Microtubule organizing center	RANBP1, IFT52, MAPRE1, CEP152, CNTRL, STIL, AUNIP, KNSTRN, CRACR2A, CEP85, HAUS8, ESPL1, ODF2, PLK4, KIF2C, CEP78, SCLT1, CCNB2, PLK1, C2CD3, CCDC96, CCNE2, AURKB, NPM1, MKS1, TACC3, RAD18, WDR62, DYNLL1, CDC45, ATP6V1D, E2F1, RAB11A, BCCIP, CHD4, CCT4, CDK2, RAN, KIF23, CCT5, CENPU, TSEN2, LEO1, PCLAF, RAB8A, KIF5B, HASPIN, H2AX, RRP7A, HAUS7, CCDC124, SPDL1, RAD51, SPAG5, TTLL12, NUP93, TXNDC9, CDC20, KIF18A, AJUBA, BUB1B, TEDC2
57	918	2.059	Transferase complex	POLR3B, TAF2, POLR1A, RAD18, DLD, KCTD17, PRPS2, SUPT20H, TUSC3, POLD2, CCND3, CDC20, RPN2, CDK2, CCNH, TEX10, DCUN1D5, RTF1, BARD1, WDR61, CCNA2, SKP2, PDSS1, PFKM, ANAPC1, CCNB2, TERT, NAA20, CCNE2, POLE, TAF9B, GTF2F2, PIK3R4, RAD51, CLNS1A, SNRPD3, PRMT5, ERCC2, EZH2, BCCIP, UBE2S, RPAP2, STT3A, CDK4, SNRPG, SENP3, KBTBD6, LEO1, UBE2C, UBE2N, HDAC2, PRKAA1, MED27, RIOK1, IFIT5, GLMN, BUB1B
56	423	4.390	Chromosomal region	ORC1, GAR1, MACROH2A1, NHP2, ORC5, MCM7, FEN1, SPDL1, OIP5, KNSTRN, CDCA8, CENPO, INCENP, DSN1, BUB3, MAD2L1, ZWILCH, CENPW, XRCC3, KNL1, PBRM1, TERT, NUP160, RAD51, NUP37, SPAG5, DYNLL1, BIRC5, NUP98, NUP107, CHD4, CENPA, NUP85, PIF1, KIF2C, BUB1B, PLK1, AURKB, H2AX, HDAC2, BLM, RIF1, CENPM, SUV39H1, EZH2, NCAPG, KIF18A, CDK2, NGDN, WRAP53, CENPU, SUV39H2, CENPN, ESCO2, KIF22, CDCA5
52	952	1.811	Nuclear body	SRRT, SRSF7, WRAP53, RAD51, NOP58, SNRNP40, RAD18, SPAG5, KIF22, DDX1, RIF1, HNRNPM, SNRPD3, CSTF2, OIP5, NUP98, RPN2, DDX39A, CDK2, SNRPB2, HABP4, SNRPA1, PRKAA1, PSMB7, KNL1, CENPO, BARD1, PIP5K1A, NCAPG2, MKI67, INCENP, DSN1, CDC25C, TERT, API5, RNF34, PPIH, SF3A3, ALYREF, H2AX, BLM, CNOT7, EEF1AKMT2, LYRM4, DDX20, PHF5A, GAR1, DKC1, NHP2, MAGOH, NOLC1, NPM1
51	672	2.517	Centrosome	RANBP1, CEP152, STIL, AUNIP, KNSTRN, CEP85, HAUS8, ESPL1, ODF2, PLK4, KIF2C, CEP78, CCNB2, PLK1, C2CD3, CCNE2, AURKB, NPM1, MKS1, TACC3, RAD18, WDR62, DYNLL1, CDC45, ATP6V1D, MAPRE1, E2F1, RAB11A, BCCIP, CHD4, CCT4, CNTRL, CDK2, KIF23, CCT5, SCLT1, CENPU, TSEN2, LEO1, PCLAF, RAB8A, KIF5B, HASPIN, H2AX, RRP7A, HAUS7, IFT52, NUP93, TXNDC9, CDC20, SPAG5
50	459	3.612	Spindle	SPDL1, WDR62, TPX2, BIRC5, MAPRE1, FAM83D, KIF18A, AUNIP, KNSTRN, CDCA8, ESPL1, NUSAP1, PLK4, KIF2C, INCENP, PLK1, AURKB, PRC1, ZWILCH, TACC3, SPAG5, KIF22, RIF1, DYNLL1, NUDC, CDC6, RAB11A, ERCC2, BCCIP, KIF20A, CEP85, HAUS8, DCUN1D5, KIF23, PKP4, SPATA5, BUB1B, RACGAP1, MAD2L1, HASPIN, NPM1, TUBB4B, HAUS7, KIF4A, TTLL12, CDC20, CNTRL, NUP85, MAP7D3, ODF2
50	884	1.876	Intracellular protein-containing complex	POLR3B, PSMA4, TAF2, POLR1A, RAD18, RTCB, KCTD17, PSMC6, PSMC1, SUPT20H, POLD2, PSMD11, CDC20, CCNH, RNASEH2B, PSMB7, DCUN1D5, RTF1, BARD1, WDR61, SKP2, ANAPC1, PSMD6, PSMC3, FAM98B, NAA20, POLE, TAF9B, GTF2F2, CNOT7, DDX1, RAD51, HSP90AB1, ERCC2, UBE2S, RPAP2, EIF4E2, TERT, VCP, KBTBD6, LEO1, UBE2C, UBE2N, RAD23B, PSME3, PRKAA1, UBQLN1, MED27, GLMN, BUB1B
48	1127	1.412	Supramolecular fiber	KIF22, TPX2, BIRC5, MAPRE1, COTL1, KIF20A, KIF18A, TUBA1B, AIF1L, KNSTRN, HAUS8, KIF23, DIAPH3, KIF2C, TUBA1C, KIF5B, CSRP2, AURKB, TUBB4B, ZWILCH, CLDN11, SPAG5, FBLN1, CORO1A, RAB11A, CCT4, FKBP1B, ANXA1, CCT7, SRPRB, CCT6A, CCT5, CCT2, PLK1, HAUS7, DYNLL1, NUDC, KIF4A, LMNB1, SPTBN1, HABP4, ODF2, NUSAP1, MNS1, INCENP, RACGAP1, LMNB2, PRC1
47	879	1.773	Mitochondrial envelope	NDUFAB1, BAK1, BCS1L, TIMM13, COA1, NDUFC1, GRPEL1, SLC25A19, ROMO1, TOMM40, SFXN2, SFXN1, VDAC2, PRELID1, CYCS, COQ2, TMEM70, COX5A, UQCC3, VDAC1, MRPL4, MRPS30, MRPL3, MRPL37, MRPL47, MRPL48, TOMM34, CHCHD2, PNPT1, TMEM177, STOML2, PHB1, LETM1, ATAD3A, MTFP1, ALDH18A1, STARD13, SORD, NRGN, CDC25C, TMEM126A, UCP2, MTARC1, MAOA, TMX2, ADAP2, CYP24A1
43	287	4.968	Condensed chromosome	SPDL1, RAD51, NCAPG, KNSTRN, SMC2, CENPO, INCENP, DSN1, BUB3, MAD2L1, ZWILCH, CENPW, KNL1, PBRM1, NUP160, NUP37, SPAG5, RIF1, DYNLL1, BIRC5, NUP98, NUP107, CENPA, NUP85, KIF2C, MKI67, BUB1B, PLK1, BANF1, AURKB, HMGB1, BLM, CENPM, MACROH2A1, NCAPH, KIF18A, CDK2, NCAPG2, CENPU, CENPN, H2AX, KIF22, SUV39H1
43	872	1.635	Polymeric cytoskeletal fiber	KIF22, TPX2, BIRC5, MAPRE1, COTL1, KIF20A, KIF18A, TUBA1B, AIF1L, KNSTRN, HAUS8, KIF23, DIAPH3, KIF2C, TUBA1C, KIF5B, AURKB, TUBB4B, ZWILCH, CLDN11, SPAG5, CORO1A, RAB11A, CCT4, ANXA1, CCT7, SRPRB, CCT6A, CCT5, CCT2, PLK1, HAUS7, DYNLL1, NUDC, KIF4A, LMNB1, ODF2, NUSAP1, MNS1, INCENP, RACGAP1, LMNB2, PRC1

**Table 7:** List of the down-regulated genes from Cluster 4 obtained by comparing DU145-shCtl cells, DU-shYAP1 and DU145-shCtl treated with 1µM of Jasplakinolide (Jasp.).

nGenes	Pathway Genes	Fold Enrichment	Pathway	Genes
14	68	6.515	Mitochondrial respiratory chain complex I	NDUFB4, NDUFB3, NDUFA8, NDUFA1, NDUFA10, NDUFA2, NDUFB10, NDUFS2, NDUFS4, NDUFB6, NDUFV1, NDUFV2, NDUFB1, NDUFA7
14	68	6.515	NADH dehydrogenase complex	NDUFB4, NDUFB3, NDUFA8, NDUFA1, NDUFA10, NDUFA2, NDUFB10, NDUFS2, NDUFS4, NDUFB6, NDUFV1, NDUFV2, NDUFB1, NDUFA7
22	114	6.107	Mitochondrial respirasome	NDUFB4, SDHA, NDUFB3, NDUFA8, NDUFA1, NDUFA10, NDUFA2, NDUFB10, SDHC, NDUFS2, NDUFS4, NDUFB6, NDUFV1, NDUFV2, NDUFB1, UQCR10, NDUFA7, UQCRC2, COX7B, COX5B, COX15, NNT
22	121	5.754	Respirasome	NDUFB4, SDHA, NDUFB3, NDUFA8, NDUFA1, NDUFA10, COX7B, NDUFA2, NDUFB10, SDHC, NDUFS2, NDUFS4, NDUFB6, NDUFV1, NDUFV2, NDUFB1, UQCR10, NDUFA7, UQCRC2, COX5B, COX15, NNT
20	110	5.754	Respiratory chain complex	NDUFB4, SDHA, NDUFB3, NDUFA8, NDUFA1, NDUFA10, COX7B, NDUFA2, NDUFB10, SDHC, NDUFS2, NDUFS4, NDUFB6, NDUFV1, NDUFV2, NDUFB1, UQCR10, NDUFA7, UQCRC2, COX5B
31	188	5.218	Inner mitochondrial membrane protein complex	SAMM50, MTX1, NDUFB4, SDHA, MICU1, ATP5F1B, NDUFB3, NDUFA8, NDUFA1, NDUFA10, NDUFA2, TIMM17A, ATP5MC2, NDUFB10, AFG3L2, SDHC, ATP5F1A, ATP5MJ, NDUFS2, NDUFS4, NDUFB6, NDUFV1, ATP5PD, NDUFV2, NDUFB1, UQCR10, ATP5PO, NDUFA7, UQCRC2, COX5B, COX7B
24	154	4.932	Oxidoreductase complex	CYBA, NDUFB4, SDHA, NDUFB3, NDUFA8, NDUFA1, NDUFA10, NDUFA2, NDUFB10, SDHC, NDUFS2, NDUFS4, NDUFB6, NDUFV1, PDHB, NDUFV2, NDUFB1, UQCR10, BCKDHA, NDUFA7, UQCRC2, IDH3B, GPD2, ETFB
51	339	4.761	Mitochondrial protein-containing complex	SAMM50, MTX1, MRPS17, NDUFB4, SDHA, MICU1, ATP5F1B, NDUFB3, NDUFA8, MRPS14, NDUFA1, MRPS26, NDUFA10, NDUFA2, DAP3, TIMM17A, ATP5MC2, NDUFB10, AFG3L2, SDHC, ATP5F1A, ATP5MJ, NDUFS2, TOMM40L, MRPL55, NDUFS4, NDUFB6, MRPL16, NDUFV1, ATP5PD, PDHB, NDUFV2, NSUN3, NDUFB1, UQCR10, ATP5PO, BCKDHA, NDUFA7, MRPS33, MRPS31, MRPS27, MRPL24, MRPL57, MRPL33, MRPS21, UQCRC2, IDH3B, COX5B, MCCC1, COX7B, SUCLG2
15	101	4.700	Organellar ribosome	MRPS17, MRPS14, MRPS26, DAP3, MRPL55, MRPL16, MRPL57, NSUN3, MRPS33, MRPS31, MRPS27, MRPL24, MRPL33, MRPS21, NDUFA7
68	559	3.849	Mitochondrial inner membrane	SAMM50, MTX1, COX15, MCUR1, NDUFB4, SDHA, MICU1, ATP5F1B, NDUFB3, NDUFA8, NDUFA1, NDUFA10, NDUFA2, TIMM17A, ATP5MC2, NDUFB10, AFG3L2, PPOX, SDHC, ATP5F1A, ATP5MJ, NDUFS2, NDUFS4, NDUFB6, NDUFV1, ATP5PD, MTLN, NDUFAF3, NDUFV2, NDUFB1, UQCR10, ATP5PO, NDUFA7, MRPS33, MRPS31, MRPS27, MRPS14, MRPS26, DAP3, MRPL24, MRPL55, MRPL16, MRPL57, MRPS17, MRPL33, MRPS21, COQ5, COQ6, IFI6, UQCRC2, CCDC51, TYMS, SPHK2, FECH, SLC25A11, NNT, SLC25A35, COX7B, ABCB10, COX5B, AIFM1, CPT2, FDXR, DHFR2, NME4, TIMMDC1, GPD2, ACAD11
63	520	3.834	Mitochondrial matrix	MRPS17, ACSM3, MRPS14, MRPS26, DAP3, MRPL55, MRPL16, PDHB, MRPL57, NSUN3, BCKDHA, MRPS33, MRPS31, ETFB, TFAM, MRPS27, PCCB, MRPL24, MRPL33, MRPS21, COASY, MCCC1, COQ5, ATP5F1B, ACADM, BLOC1S1, TYMS, ALAS1, IARS2, ME2, IDH3B, NME4, ACOT13, DARS2, NDUFA10, RIDA, TARS2, ME3, SHC1, DHFR2, SUCLG2, NDUFA7, FECH, FH, ACO2, DECR1, HIBADH, ALDH5A1, LIAS, PPA2, MMAB, ATP5F1A, NDUFS2, FDXR, ACP6, NAXE, GFM2, ALDH7A1, NUDT2, ISCA2, MRM3, GSTK1, HOGA1
25	222	3.563	Ribosomal subunit	RPL3, RPL26, RPS14, RPL38, RPL35A, RPS23, RPL23A, RPL10A, MRPS17, RPL17, MRPS14, MRPS26, DAP3, MRPL55, MRPL16, NSUN3, MRPS33, MRPS31, MRPS27, MRPL24, MRPL57, MRPL33, MRPS21, DHX29, EIF2D
70	630	3.516	Organelle inner membrane	SAMM50, MTX1, COX15, MCUR1, NDUFB4, SDHA, MICU1, ATP5F1B, NDUFB3, NDUFA8, NDUFA1, NDUFA10, NDUFA2, TIMM17A, ATP5MC2, NDUFB10, AFG3L2, PPOX, SDHC, ATP5F1A, ATP5MJ, NDUFS2, NDUFS4, NDUFB6, NDUFV1, ATP5PD, MTLN, NDUFAF3, NDUFV2, NDUFB1, UQCR10, ATP5PO, NDUFA7, MRPS33, MRPS31, MRPS27, MRPS14, MRPS26, DAP3, MRPL24, MRPL55, MRPL16, MRPL57, MRPS17, MRPL33, MRPS21, COQ5, COQ6, IFI6, UQCRC2, CCDC51, TYMS, SPHK2, FECH, SLC25A11, NNT, SLC25A35, COX7B, ABCB10, COX5B, ITPR1, AIFM1, CPT2, FDXR, DHFR2, PLPP6, NME4, TIMMDC1, GPD2, ACAD11
30	283	3.354	Ribosome	RPL3, RPL26, RPS14, RPL38, RPL35A, RPS23, RPL23A, RPL10A, MRPS17, RPL17, MRPS14, MRPS26, DAP3, BTF3, EIF3H, MRPL55, MRPL16, MRPL57, NSUN3, MRPS33, MRPS31, MRPS27, MRPL24, MRPL33, MRPS21, DHX29, DHX9, EIF2D, RPL22L1, NDUFA7
79	828	3.019	Mitochondrial membrane	SAMM50, MTX1, COX15, MCUR1, NDUFB4, SDHA, TMEM14A, MICU1, ATP5F1B, NDUFB3, NDUFA8, NDUFA1, NDUFA10, NDUFA2, TIMM17A, ATP5MC2, TMEM14B, NDUFB10, AFG3L2, PPOX, SDHC, ATP5F1A, ATP5MJ, NDUFS2, TOMM40L, NDUFS4, NDUFB6, NDUFV1, ATP5PD, MTLN, NDUFAF3, NDUFV2, NDUFB1, UQCR10, ATP5PO, NDUFA7, MRPS33, MRPS31, MRPS27, MRPS14, MRPS26, DAP3, MRPL24, MRPL55, MRPL16, MRPL57, MRPS17, MRPL33, MRPS21, COQ5, ACADM, COQ6, IFI6, COX7B, ABCB10, COX5B, UQCRC2, CCDC51, TYMS, ACAD11, SPHK2, FECH, SLC44A1, SLC25A11, NNT, TIMMDC1, SLC25A35, AIFM1, CPT2, CALM3, FDXR, DHFR2, IKBKE, COASY, ACACB, STARD7, NME4, GPD2, CYB5A
82	879	2.952	Mitochondrial envelope	SAMM50, MTX1, COX15, MCUR1, NDUFB4, SDHA, TMEM14A, MICU1, ATP5F1B, NDUFB3, NDUFA8, NLN, NDUFA1, NDUFA10, NDUFA2, TIMM17A, ATP5MC2, TMEM14B, NDUFB10, AFG3L2, PPOX, SDHC, ATP5F1A, ATP5MJ, NDUFS2, TOMM40L, NDUFS4, NDUFB6, NDUFV1, ATP5PD, MTLN, NDUFAF3, NDUFV2, NDUFB1, UQCR10, ATP5PO, NDUFA7, MRPS33, MRPS31, MRPS27, MRPS14, MRPS26, DAP3, MRPL24, MRPL55, MRPL16, MRPL57, MRPS17, MRPL33, MRPS21, NME4, COQ5, ACADM, COQ6, IFI6, COX7B, BLOC1S1, ABCB10, COX5B, UQCRC2, AIFM1, CCDC51, TYMS, ACAD11, SPHK2, FECH, SLC44A1, SLC25A11, NNT, TIMMDC1, SLC25A35, FKBP10, CPT2, CALM3, FDXR, DHFR2, IKBKE, COASY, ACACB, STARD7, GPD2, CYB5A
157	1830	2.715	Mitochondrion	NDUFB4, FH, SAMM50, ACO2, SLC25A11, ATP5F1B, ALDH5A1, UQCRC2, SCCPDH, ATP5F1A, AIFM1, CYB5R1, ATP5PD, MTX1, MRPS17, ATP5PO, BCKDHA, ACSM3, COX15, ALAS1, MCUR1, GATB, ME1, FECH, IARS2, SDHA, MCCC1, ME2, MRPS33, TMEM14A, IDH3B, DECR1, HIBADH, MICU1, MRPS27, TIMMDC1, PCCB, GPD2, ACADM, PRDX6, DARS2, NDUFB3, NDUFA8, COQ6, MRPS14, LIAS, ACO1, NLN, ATPAF1, NDUFA1, MRPS26, IFI6, PNKD, QRSL1, NDUFA10, COX7B, NDUFA2, RIDA, DAP3, TIMM17A, ATP5MC2, NIPSNAP3A, TMEM14B, PPA2, NDUFB10, AFG3L2, PPOX, SDHC, MRPL24, ME3, ATP5MJ, CPT2, NDUFS2, TOMM40L, GATD3, FDXR, TMEM143, ACP6, MRPL55, NAXE, NDUFS4, NDUFB6, TRUB1, ISCA2, MRPL16, NDUFV1, PDHB, SUCLG2, MRPL57, MTLN, TYMS, NDUFAF3, NDUFV2, NSUN3, DHFR2, NDUFB1, UQCR10, NIPSNAP1, GSTK1, ECI2, ACAD11, HOGA1, MRPL33, NDUFA7, MRPS31, ETFB, TFAM, MRPS21, COASY, SLC44A1, ACACB, CPNE3, NME4, COQ5, NNT, NENF, IRF3, BLOC1S1, ABCB10, COX5B, PARP1, CCDC51, ALDH7A1, MRM3, METAP1D, SUGCT, ADSS2, DGAT2, SPHK2, STARD7, ASB9, PON2, ACOT13, PECR, SCP2, APH1A, SLC25A35, ENOSF1, AP3B1, MMAB, FKBP10, TARS2, MSRB2, FADS1, CALM3, SHC1, NCSTN, GFM2, NAIF1, TDRKH, ANXA6, PSENEN, CSKMT, IKBKE, MAPK3, NUDT2, CYB5A
102	1380	2.339	Organelle envelope	SAMM50, MTX1, COX15, TPR, MCUR1, NDUFB4, NUP133, SDHA, SEH1L, MGST2, TMEM14A, MICU1, ATP5F1B, NDUFB3, GLE1, NDUFA8, NLN, NDUFA1, NDUFA10, NDUFA2, TIMM17A, ATP5MC2, TMEM14B, NDUFB10, AFG3L2, PPOX, SDHC, ATP5F1A, ATP5MJ, NDUFS2, TOMM40L, NDUFS4, NDUFB6, NDUFV1, ATP5PD, MTLN, NDUFAF3, NDUFV2, NDUFB1, UQCR10, TOR3A, IPO9, ATP5PO, NDUFA7, MRPS33, MRPS31, MRPS27, MRPS14, MRPS26, DAP3, MRPL24, MRPL55, MRPL16, MRPL57, MRPS17, MRPL33, MRPS21, ENO1, SEPHS1, MAPK3, NME4, COQ5, ACADM, COQ6, IFI6, COX7B, BLOC1S1, ABCB10, COX5B, UQCRC2, PARP1, CETN2, AIFM1, CCDC51, TYMS, THAP7, S100A6, PLPP6, ACAD11, SPHK2, FECH, NUCB2, SLC44A1, SLC25A11, NNT, TIMMDC1, CACYBP, SLC25A35, TEX2, FKBP10, ITPR1, CPT2, CALM3, FDXR, SHISA5, DHFR2, IKBKE, COASY, ACACB, STARD7, GPD2, CYB5A
102	1380	2.339	Envelope	SAMM50, MTX1, COX15, TPR, MCUR1, NDUFB4, NUP133, SDHA, SEH1L, MGST2, TMEM14A, MICU1, ATP5F1B, NDUFB3, GLE1, NDUFA8, NLN, NDUFA1, NDUFA10, NDUFA2, TIMM17A, ATP5MC2, TMEM14B, NDUFB10, AFG3L2, PPOX, SDHC, ATP5F1A, ATP5MJ, NDUFS2, TOMM40L, NDUFS4, NDUFB6, NDUFV1, ATP5PD, MTLN, NDUFAF3, NDUFV2, NDUFB1, UQCR10, TOR3A, IPO9, ATP5PO, NDUFA7, MRPS33, MRPS31, MRPS27, MRPS14, MRPS26, DAP3, MRPL24, MRPL55, MRPL16, MRPL57, MRPS17, MRPL33, MRPS21, ENO1, SEPHS1, MAPK3, NME4, COQ5, ACADM, COQ6, IFI6, COX7B, BLOC1S1, ABCB10, COX5B, UQCRC2, PARP1, CETN2, AIFM1, CCDC51, TYMS, THAP7, S100A6, PLPP6, ACAD11, SPHK2, FECH, NUCB2, SLC44A1, SLC25A11, NNT, TIMMDC1, CACYBP, SLC25A35, TEX2, FKBP10, ITPR1, CPT2, CALM3, FDXR, SHISA5, DHFR2, IKBKE, COASY, ACACB, STARD7, GPD2, CYB5A

**Table 8:** List of the « Cellular components » extracted from the 7726 genes obtained after comparison between NCI-H660 and PC3 treated with Jasp. and PC3 cells and between PC3 treated by Jasplakinolide (Jasp.) *versus* PC3 cells subjected to ShinyGO.

DESCRIPTION	P VALUE	NUMBER OF GENES
Intracellular	9.61E-81	6077
Intracellular organelle	7.64^E^-64	5420
Organelle	1.55E-59	5723
Intracellular membrane-bounded organelle	1.7E-55	4753
Membrane-bounded organelle	3.36E-53	5318
Cytoplasm	3.15E-50	4951
Nucleus	1.08E-27	3270
Cytosol	2.7E-27	2413
Cellular anatomical entity	3.31E-24	6856
Intracellular organelle lumen	5.1E-21	2606
Nucleoplasm	2.06E-20	1855
Non-membrane-bounded organelle	5.1E-17	2180
Intracellular non-membrane-bounded organelle	8.05E-17	2174
Nuclear lumen	6.26E-16	2105
Mitochondrion	6.43E-13	804
Protein-containing complex	6.51E-13	2197
Organelle membrane	8.37E-12	1582
Organelle envelope	1.16E-11	622
Microtubule cytoskeleton	7.38E-11	617
Cytoskeleton	5E-10	1025
Catalytic complex	2.38E-09	648
Endomembrane system	2.68E-08	1917
Microtubule organizing center	6.18E-08	392
Mitochondrial envelope	1.16E-11	622
Mitochondrial membrane	1.67E-07	371
Centrosome	3.06E-07	300
Nucleolus	3.75E-07	434
Mitochondrial matrix	2E-06	255
Organelle inner membrane	3.54E-06	280
Mitochondrial inner membrane	3.54E-06	280
Vesicle	6.15E-06	1615
Anchoring junction	8.95E-06	395
Bounding membrane of organelle	8.99E-06	918
Focal adhesion	1.4E-05	215
Transferase complex	1.46E-05	364
Golgi apparatus	1.55E-05	705
Cell-substrate junction	2.1E-05	217
Extracellular exosome	2.57E-05	907
Nuclear envelope	3.24E-05	241
Mitochondrial protein complex	3.22E-05	148
Extracellular vesicle	3.2E-05	914
Extracellular organelle	3.63E-05	915
Chromosome	0.00013	741
Chromosome, centromeric region	0.00017	111
Spindle	0.00025	181
Perinuclear region of cytoplasm	0.00029	338
Chromosomal region	3E-04	165
Nuclear membrane	0.00039	157
Kinetochore	0.00041	81
Whole membrane	0.00041	733
Vacuole	0.00047	362
Lysosome	5E-04	323
Intracellular vesicle	0.00053	995
Cytoplasmic vesicle	0.00058	993
Cell junction	0.00055	868
Actin cytoskeleton	9E-04	228
Golgi membrane	0.00095	345
Nuclear body	0.0012	355
Microtubule	0.0012	197
Condensed chromosome, centromeric region	0.0014	71
Nuclear periphery	0.0014	77

**Table 9:** Prediction of ciliary genes from CiliaCarta.

Gene name	Description	CiliaCarta Rank	CiliaCarta Score
GLI1	GLI family zinc finger 1	5343	-4.899048466
IFT20	Intraflagellar transport 20 homolog	350	1.819149696
Arl13B	ADP-ribosylation factor-like 13b	13	7.514714013
IFT80	Intraflagellar transport 80 homolog	95	4.863543816
IFT88	Intraflagellar transport 88 homolog	1	11.69564737

## Data Availability

All datasets generated and analyzed during this study are included in this published article and its Supplementary Information files. Additional data are available from the corresponding author on reasonable request.
