# Supplementary material for: Primary Cilium Forces Neuroendocrine Shift in Prostate Cancer through YAP1 Repression and Reduced Mitochondrial Activity
Source: Theranostics. 2026 May 18;16(12):6861–91. doi: 10.7150/thno.126688 (PMC13232477; doi:10.7150/thno.126688)
Supplement: Supplementary file 1 — Supplementary figures. [file thnov16p6861s1.pdf]

Figure S1. (**A**) Gleason scores of the 550 patients analyzed based on the GLI1/IFT20 signature extracted from the TCGA PRAD database. (**B**) Volcano plot of the distribution of all differentially expressed genes based on the $GLI1^{+}/IFT20+$ ciliated cells *versus* non ciliated cell signature ($GLI1^{+}/IFT20^{-}$, $GLI1^{-}/IFT20^{-}$, $GLI1^{-}/IFT0^{-}$). (**C**) KEGG pathway enrichment of genes downregulated in $GLI1^{+}/IFT20^{+}$ prostate tumors (TCGA-PRAD). Bar plot showing the top KEGG pathways enriched among genes significantly downregulated in the 5 % of prostate cancer patients from the TCGA-PRAD dataset co-expressing GLI1 and IFT20. This subgroup also exhibits strong overexpression of Synaptophysin (SYP), repression of RB1, and a trend toward increased YAP1 expression. The most significantly enriched pathway is oxidative phosphorylation. For each pathway, the Gene Ratio (proportion of input genes involved in the pathway) and the adjusted p-value (p.adjust) are indicated (**D**) Reactome pathway enrichment of genes downregulated in $GLI1^{+}/IFT20^{+}$ prostate tumors (TCGA-PRAD). Bar plot showing the top enriched Reactome pathways among genes significantly downregulated in the 5 % of prostate cancer patients from the TCGA-PRAD dataset co-expressing GLI1 and IFT20. The most significantly enriched pathway is Respiratory electron transport. For each pathway, the Gene Ratio (proportion of input genes involved in the pathway) and the adjusted p-value (p.adjust) are indicated (**E**) GO Biological Process enrichment of genes downregulated in $GLI1^{+}/IFT20^{+}$ prostate tumors (TCGA-PRAD). Bar plot showing the top enriched GO biological processes among genes significantly downregulated in the 5 % of prostate cancer patients co-expressing GLI1 and IFT20. The most enriched terms include mitochondrial ATP synthesis coupled electron transport**,** aerobic respiration, and oxidative phosphorylation. (**F**) Schematic representation of the different zones used in localized patients (normal zone, peritumoral zone and tumoral zone) and in CRPC patients (tumoral zone). Created in https://BioRender.com. (**G**) Axial CT scan (non-contrast) from patients 1, 2, 3 and 5 from Antoine Lacassagne Center.

Figure S2. (**A-C**) Representative immunofluorescence of patients 2 (**A**), 3 (**B**), 4 (**C**) and 5 (**D**) from Centre Antoine Lacassagne. Immunofluorescence labeled with Pericentrin (green), Arl13b (red), panNE (yellow), panCK (grey) and DAPI (blue). Samples were studied to evaluate the prediction model of the absence or presence of the PC in a putative NE zone.

Figure S3. (A) Positive control condition showing nuclear staining with DAPI (blue) and robust expression of the proliferation marker KI67 (yellow), confirming correct KI67 detection. Co-staining with pan-cytokeratin (pan-CK) identifies proliferating epithelial cells. **(B)** Immunofluorescence analysis of CRPC patient tumor sections, stained for ARL13B (red), Pericentrin (green), KI67 (yellow) and DAPI (blue), with pan-NE marking neuroendocrine tumor areas. Higher-magnification views highlight NE cell regions lacking KI67 expression.

Figure S4. (**A**) Cell lysates from P69 cells exposed to hypoxia (Hx 1%) treated 48h with Gefitinib (Gef. 10µM) were analyzed by immunoblot using HIF-1α protein, along with β-tubulin as loading control. Immunoblot (inset) and quantification of HIF-1α expression. (**B**) Quantification of the transcriptional expression of CPT1 obtained through RT-qPCR conducted on P69 cells treated with 100µM of Clofibrate. (**C**) Quantification of the percentage of ciliated cells in P69 cell treated with Gefitinib (10µM) and Clofibrate (100µM) in hypoxia (Hx – 1% O_2) for 48h (n>250). (**D**) Cell viability of NCI-H660 cells cultured under normoxic (Nx) or hypoxic (Hx 1%)

conditions for 3 days (D3) and 6 days (D6). (**E**) Immunofluorescence of LNCaP, DU145 and PC3 cells exposed to normoxia (Nx) and hypoxia (Hx – 1% O_2) for 72h and labeled with acetylated α-tubulin (acetylated α-tub.) (red) and Arl13b (green) along with DAPI (blue) (magnification: x60, scale bar 10 μm). (**F**) Cell lysates from DU145 and PC3 cells exposed to hypoxia (Hx 1%) treated 48h with Gefitinib (Gef. 10μM) were analyzed by immunoblot using HIF-1α protein, along with β-tubulin as loading control. Quantification of HIF-1α expression. (**G-H**) Quantification of the transcriptional expression of CPT1 obtained through RT-qPCR conducted on DU145 (**G**) and PC3 (**H**) cells treated with 100μM of Clofibrate. (**I**) Quantification of the percentage of ciliated cells in DU145 cells treated with Gefitinib (10μM) and Clofibrate (100μM) in normoxia (Nx) and hypoxia (Hx) for 48h (n>250). (**J**) Quantification of the percentage of ciliated cells in PC3 cells treated with Gefitinib (10μM) and Clofibrate (100μM) in normoxia (Nx) and hypoxia (Hx) for 48h (n>250).
All quantifications were performed using GraphPrism9 software. Statistical analysis: Two-way ANOVA; significant differences are indicated by * p<0.05 and ** p<0.005.

Figure S5. (**A**) Boyden chamber trans-well migration assay with LNCaP-shCtl and LNCaP-shYAP1 cells on collagen coated membranes with 8 μm porosity. Counts were performed after 24h. (**B**) Boyden chamber trans-well migration assay with DU145-shCtl and DU145-shYAP1 cells on collagen coated membranes with 8 μm porosity. Counts were performed after 24h. (**C**) Cell lysates from PC3 transfected with siYAP or not (siCtl) were analyzed by immunoblot using YAP1 protein, along with β-tubulin as loading control. (**D**) Immunofluorescence of PC3 cells transfected with siCtl or siYAP1 and labeled with YAP1 (red) (magnification: x60, scale bar 10 μm). (**E**) Boyden chamber trans-well migration assay with PC3 transfected with a siCtl or siYPA1 cells on collagen coated membranes with 8 μm porosity. Counts were performed after 24h. (**F**) Immunofluorescence of PC3 cells transfected with siCtl or siYAP1 and labeled with Pericentrine (green), ARL13B (red) and DAPI (blue) (magnification: x60, scale bar 10 μm). (**G**) Cell lysates from PC3 transfected with siYAP1, siTAZ, siYAP1/TAZ or control siRNA (siCtl) were analyzed by immunoblot using YAP1 and TAZ proteins, along with ERK2 as loading control. (**H**) Cell lysates from PC3 transfected with siYAP or not (siCtl) were analyzed by immunoblot using SYP protein, along with b-tubulin as loading control. Cell lysates from NCI-H660 were used as control. (**I**) Quantification of Notch4 (Notch receptor) transcript levels in (i) LNCaP cells untreated (-) or treated with enzalutamide (+), (ii) DU145 shCtl (-) or shYAP1 (+), (iii) PC3 untreated (-) or treated with Jasplakinolide (Jasp.; +) and (iv) NCI-H660 as reference. The presence of the primary cilium (PC) is indicated below each condition: (–) no cilium, (+) <10% ciliated cells, (+++) >50% ciliated cells.
Quantifications shown in panel H were performed using GraphPad Prism 9**.** Statistical analysis was conducted using two-way ANOVA. Significance is indicated as follows: *p < 0.05, **p < 0.005, ***p < 0.0005, ****p < 0.0001. For all other panels, unpaired two-tailed t-tests were used unless otherwise specified.

Figure S6. (**A**) Quantification of the transcriptional expression of CCN1 and CCN2 obtained through RT-qPCR conducted on DU145 cells treated for 5d with 1μM of Jasplakinolide (Jasp.). (**B**) Heatmap of differentially expressed genes in PC3 cells treated with Jasplakinolide. Gene expression profiles of PC3 cells treated with 1 μM Jasplakinolide (Jasp.) were compared to untreated PC3 cells. K-means clustering (Phantasus v1.19.3) identified 11 distinct gene clusters. Among them, five clusters - three with upregulated genes (in red) and two with downregulated genes (in blue) - highlight significant transcriptional changes related to

microtubule organization, centrosome function, and centromeric spindle assembly. (**C**) Immunofluorescence of PC3 cells transfected with shCtl or shYAP1, and treated with Rotenone (Rotenone, 5 mM). Cells were labeled with pericentrin (green) and Arl13b (red) along with DAPI (blue) (magnification: x60, scale bar 10 µm). PC% indicates the percentage of primary cilium–positive (ciliated) cells, quantified by manual counting (n = 388 cells). (**D**) Flow cytometry analysis of TOMM20 expression. Left: Representative histograms of TOMM20 fluorescence intensity in (red) unlabelled PC3 cells, (blue) PC3 control (Ctl), (orange) PC3 cells transfected with siYAP1, (green) PC3 cells treated with metformin (Metf.), and (dark green) PC3 cells with combined siYAP1 and metformin treatment. Right: Quantification of mean fluorescence intensity (MFI) of TOMM20 expression across conditions. TOMM20, a mitochondrial outer membrane marker, was used to assess mitochondrial content or activity. Data are presented as mean ± SD from at least three independent experiments.
Statistical significance was determined using one-way ANOVA followed by Tukey's post hoc test (*$p < 0.05$ and ****$p < 0.0001$).

Figure S7. (**A-F**) Metabolic profiling using Omnilog in PC3 and NCI-H660 cells**.** Total cell growth was measured using MMP1 plates (Biolog), which assess the ability of cells to utilize specific carbon sources. The assay was performed in the presence of (**A**) glucose, (**B**) glucose-6-phosphate (G-6-P), (**C**) glucose-1-phosphate (G-1-P), (**D**) pyruvate, succinate, and (**E**) lactate. PC3 cells are shown in red and NCI-H660 cells in blue. The Omnilog system detects metabolic activity as a readout of overall cell proliferation on each substrate, revealing distinct substrate preferences between the two prostate cancer cell lines. (**G-N**) Mitochondrial substrate utilization profiling using Omnilog in PC3 and NCI-H660 cells. Cellular mitochondrial function was assessed using MITO S1 plates (Biolog), which measure the ability of mitochondria to utilize a single carbon source as the sole energy substrate. The assay included (**G**) citrate, (**H**) Cis Aconitate, glucose-1-phosphate (G-1-P), (**I**) Isocitrate, (**J**) a-Ketoglutarate, (**K**) succinate, (**L**) fumarate, (**M**) Malate and (**N**) Tryptamine. PC3 cells are shown in red and NCI-H660 cells in blue. Experiments were performed with n = 2 independent biological replicates. Statistical analysis was conducted using a two-way ANOVA.

Figure S8. (**A**) Heatmap showing the expression of selected AR marker genes in LNCaP and LNCaP-NE cells compared to NCI-H660. Gene expression was analyzed using Phantasus (v1.19.3). (**B**) Heatmap showing the expression of selected NE and PC marker genes in LNCaP cells compared to LNCaP-NE. Gene expression was analyzed using Phantasus (v1.19.3). (**C**) Heatmap showing the expression of selected NE and PC marker genes in LNCaP-NE cells compared to NCI-H660. Gene expression was analyzed using Phantasus (v1.19.3). (**D-H**) Heatmap showing the expression of selected (**D**) Mitochondrial complexes, (**E**) Mitochondrion, (**F**) Mitochondrial envelope, (**G**) Respirasome and (**H**) Inner mitochondrial membrane protein complex marker genes in LNCaP and LNCaP-NE cells compared to NCI-H660. Gene expression was analyzed using Phantasus (v1.19.3).

Figure S9. Integrated morpho-functional analysis of mitochondrial remodeling following long-term enzalutamide treatment. (A) Radar plot showing z-score–normalized mitochondrial morpho-functional parameters extracted using the EmitoMetrix pipeline from transmission electron microscopy images acquired at a spatial scale of 500 nm. Parameters describe mitochondrial size, shape, intensity distribution, and cristae orientation. Values

represent deviations from the global mean expressed in standard deviation units and enable comparison between control cells and cells treated with enzalutamide for 2 months. **(B)** SHAP (SHapley Additive exPlanations) summary plot illustrating the relative contribution of individual mitochondrial features to the prediction of the enzalutamide-treated condition. Each dot represents a single mitochondrion, with SHAP values indicating the impact of each feature on model output. Feature values are color-coded from low (blue) to high (red). Together, these analyses reveal a coordinated mitochondrial morpho-functional signature associated with long-term enzalutamide treatment.

Figure S10. (**A–D**) Heatmap analysis of gene expression before and after enzalutamide treatment in prostate cancer patients (GSE197780). Z-score normalized expression values are shown for patient samples collected before (orange bar) and after (green bar) enzalutamide treatment. **(A)** Expression of androgen receptor (AR) pathway markers and neuroendocrine prostate cancer (NEPC) markers. **(B)** Stratification of post-treatment samples into YAP1-high and YAP1-low groups using the median YAP1 expression value (7.79). Box-plot analysis compares NE marker expression using Welch's t-test. NCAM1 was significantly higher in YAP1-high tumors ($p = 1.7 \times 10^{-5}$), ENO2 showed a borderline increase ($p = 0.050$), whereas CHGA, TP53 and MYCN showed no significant differences. **(C)** Expression of primary cilium–associated genes. **(D)** Expression of mitochondrial envelope–related genes. Each column represents an individual patient sample.

Figure S11. YAP1-expressing cells within NE-enriched clusters 8 and 11. (**A**) UMAP representation of cluster 8, highlighting the 7 YAP1-positive cells (red) among all cells in the cluster (n = 1,324). (**B**) Violin plot showing YAP1 expression levels in YAP1-positive *versus* YAP1-negative cells within cluster 8. (**C**) UMAP representation of cluster 11, highlighting the 5 YAP1-positive cells (red) among all cells in the cluster (n = 482). (**D**) Violin plot showing YAP1 expression levels in YAP1-positive versus YAP1-negative cells within cluster 11. (**E**) Expression of selected neuroendocrine markers (ASCL1, FOXA2, NKX2.1, MYCN, POU3F, INSM1, SIAH2, NCAM1, CHGA, CHGB, SYP and ENO2) in clusters 8 (blue) and 11 (pink) illustrating the range and heterogeneity of NE marker expression within these rare YAP1-expressing subsets.
All quantifications were performed using GraphPrism9 software. Statistical analysis: Two-way ANOVA; significant differences are indicated by **** $p< 0.0001$.

Figure S12. (**A**) Metabolic signature score based on the expression of *PFKFB3*, *LDHA*, *CA9*, and *MCT4* across all clusters identified by single-cell RNA-seq. Each bar represents the computed signature score for a given cluster. Clusters 8 (blue) and 11 (pink) are highlighted for emphasis.

A

TCGA PRAD - Gleason score

	6	7	8	9	10
**GLI1⁻/IFT20⁻**	32	143	39	61	2
**GLI1⁻/IFT20⁺**	4	49	14	29	0
**GLI1⁺/IFT20⁻**	10	41	10	33	2
**GLI1⁺/IFT20⁺**	0	14	1	14	0

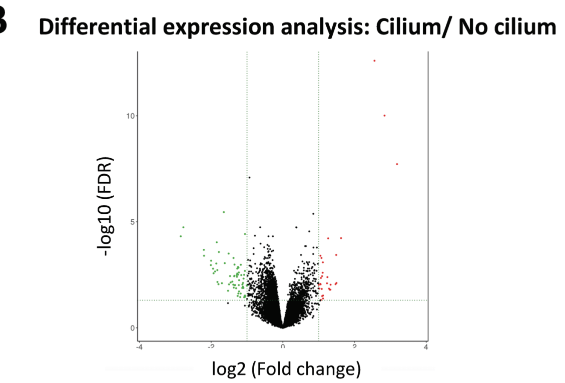

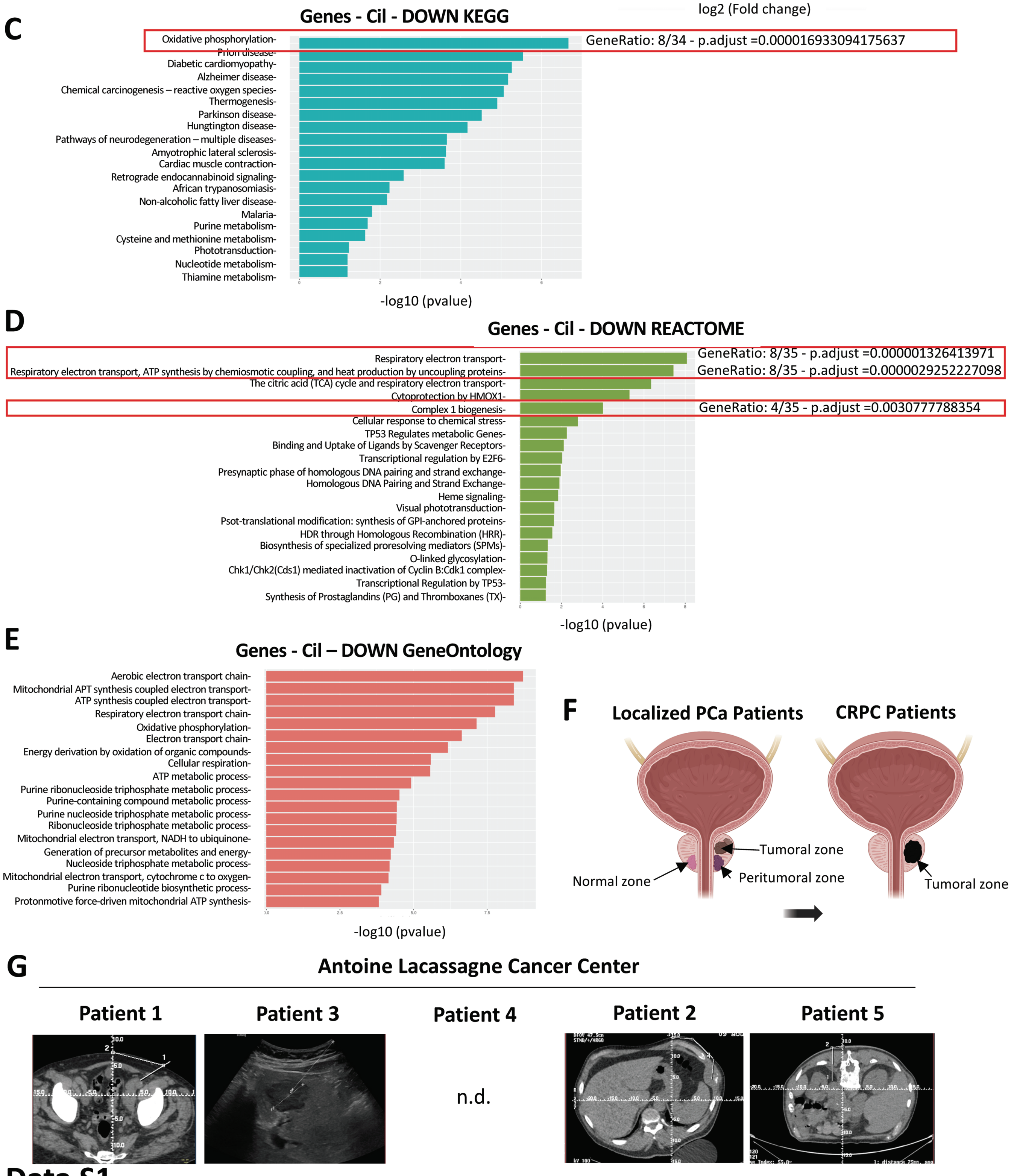

Data S1

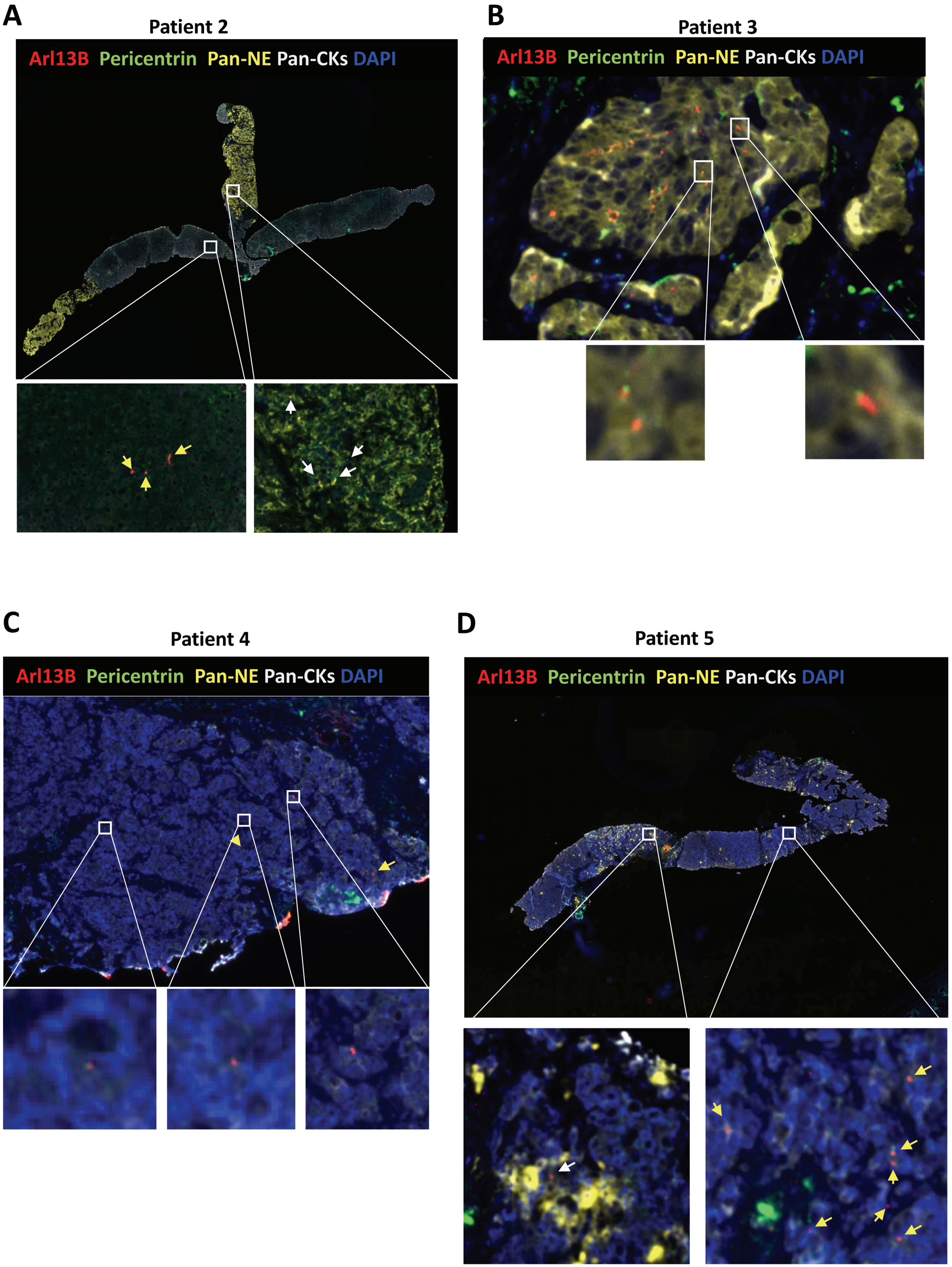
A
Patient 2
Arl13B Pericentrin Pan-NE Pan-CKs DAPI
B
Patient 3
Arl13B Pericentrin Pan-NE Pan-CKs DAPI
C
Patient 4
Arl13B Pericentrin Pan-NE Pan-CKs DAPI
D
Patient 5
Arl13B Pericentrin Pan-NE Pan-CKs DAPI

Data S2

A

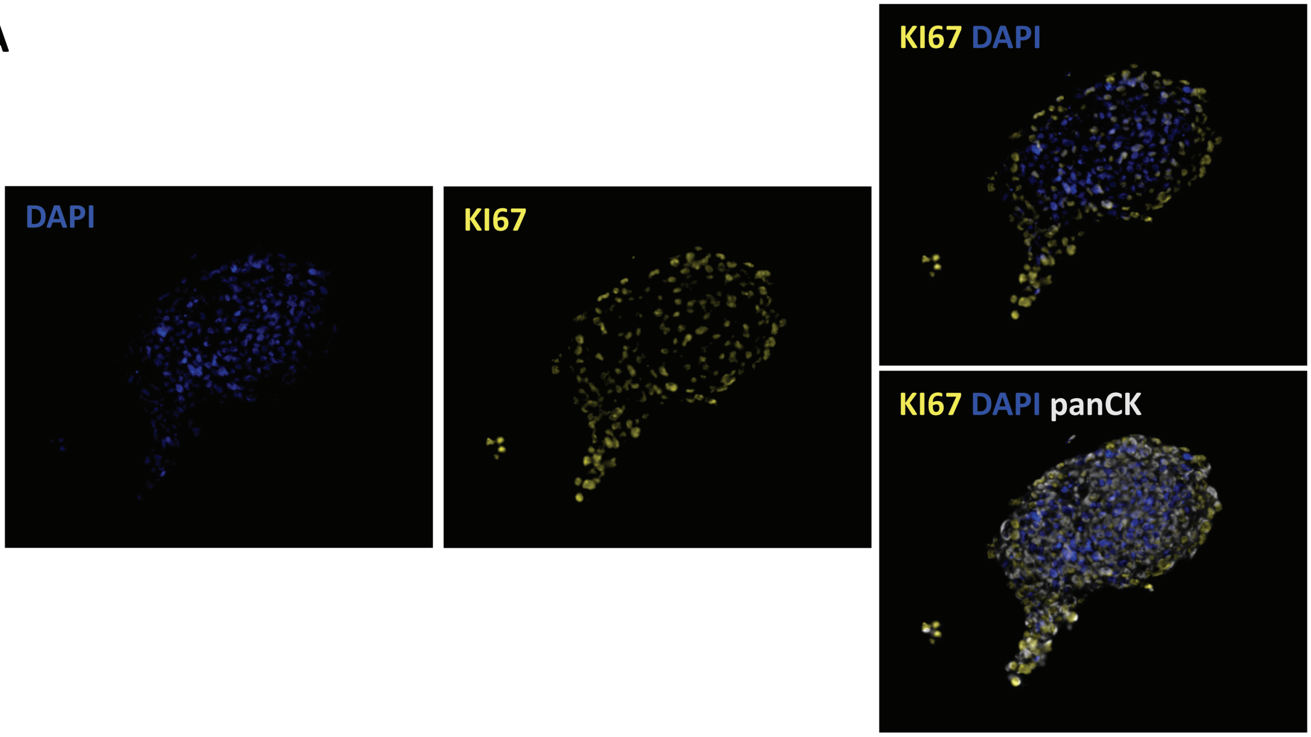
DAPI
KI67
KI67 DAPI
KI67 DAPI panCK

B

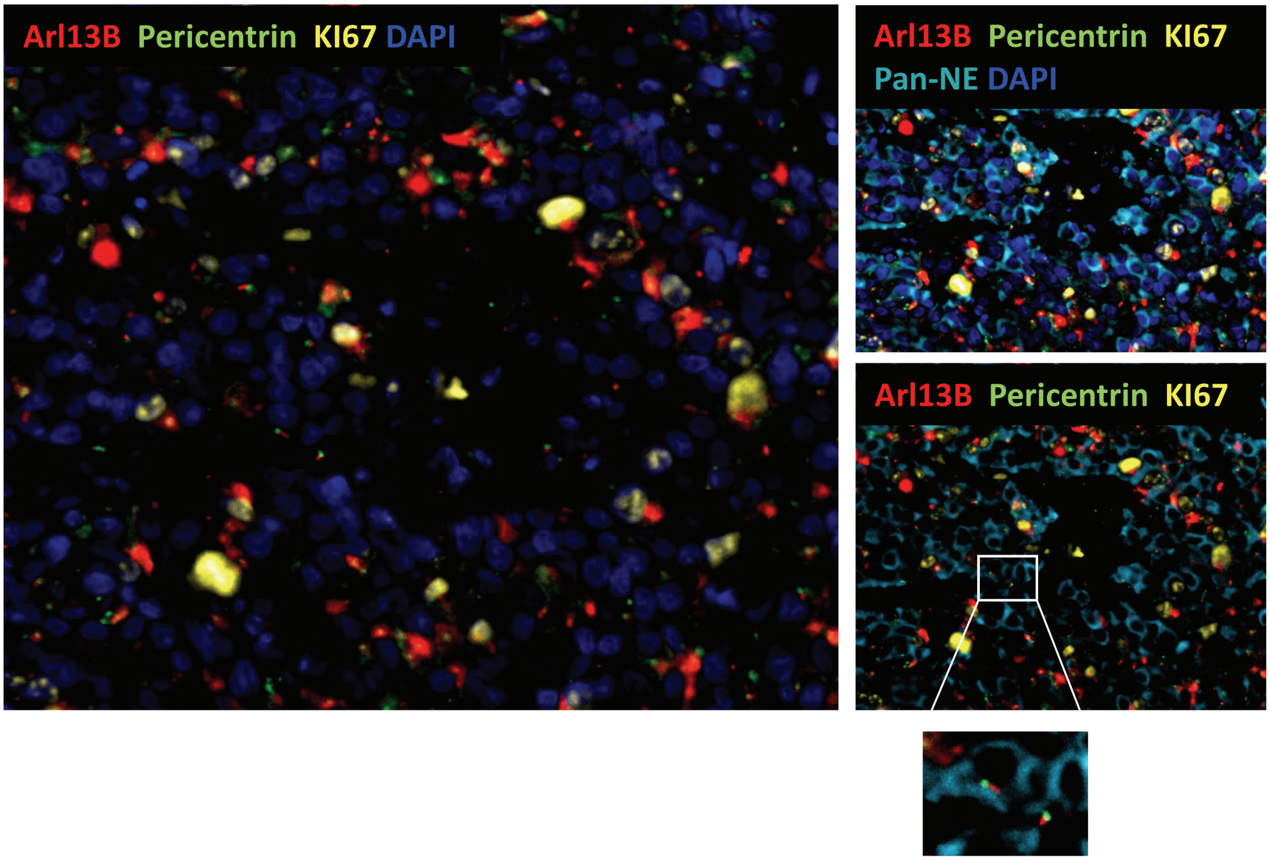
Arl13B Pericentrin KI67 DAPI
Arl13B Pericentrin KI67
Pan-NE DAPI
Arl13B Pericentrin KI67

Data S3

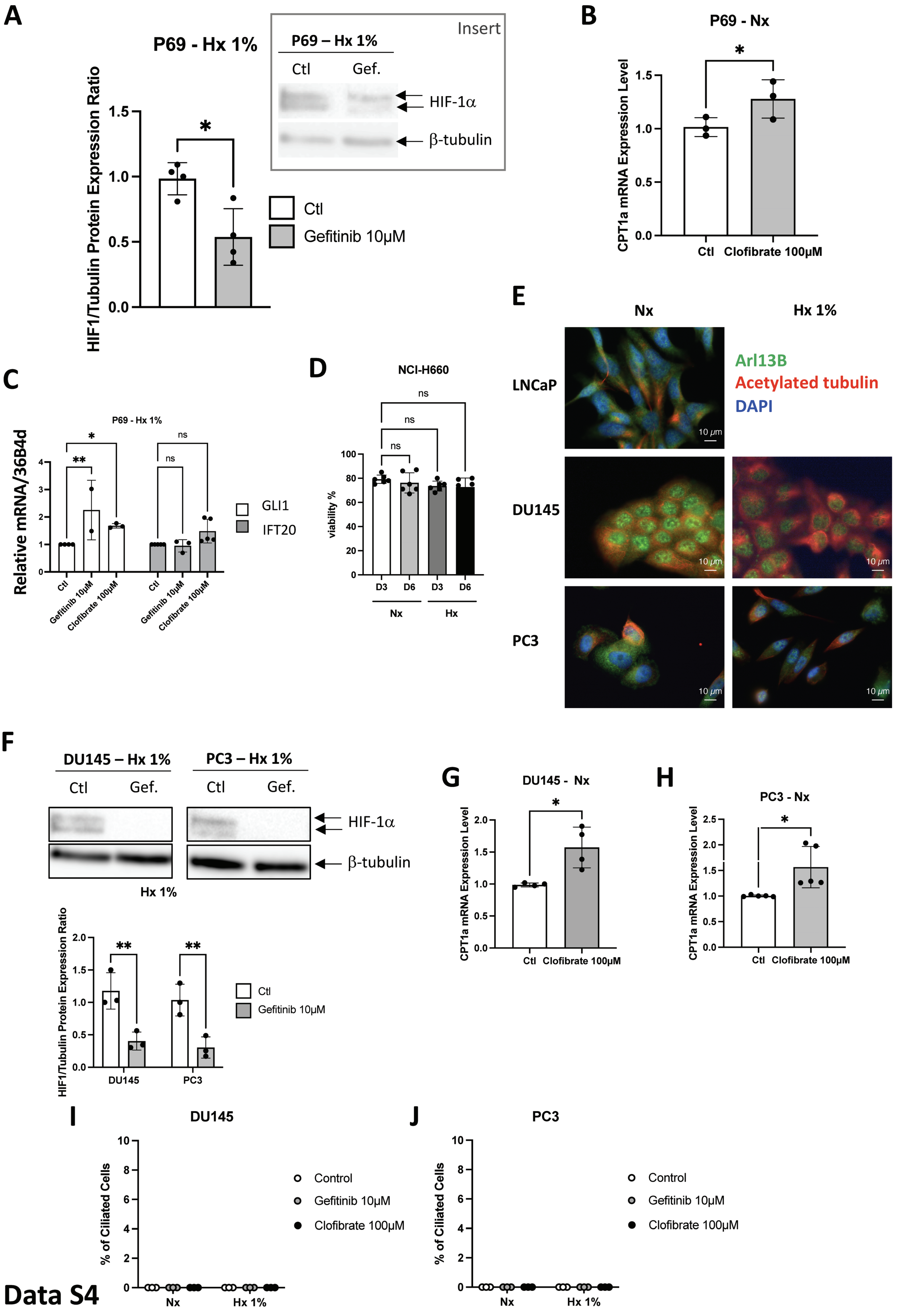
A
P69 - Hx 1%
HIF1/Tubulin Protein Expression Ratio
Insert
P69 – Hx 1%
Ctl
Gef.
HIF-1α
β-tubulin
Ctl
Gefitinib 10μM
B
P69 - Nx
CPT1a mRNA Expression Level
Ctl
Clofibrate 100μM
C
P69 - Hx 1%
Relative mRNA/36B4d
GLI1
IFT20
Gefitinib 10μM
Clofibrate 100μM
D
NCI-H660
viability %
ns
D3
D6
Nx
Hx
E
Nx
Hx 1%
LNCaP
DU145
PC3
Arl13B
Acetylated tubulin
DAPI
10 μm
F
DU145 – Hx 1%
PC3 – Hx 1%
Hx 1%
G
DU145 - Nx
H
PC3 - Nx
I
DU145
% of Ciliated Cells
Control
Gefitinib 10μM
Clofibrate 100μM
J
PC3

Data S4

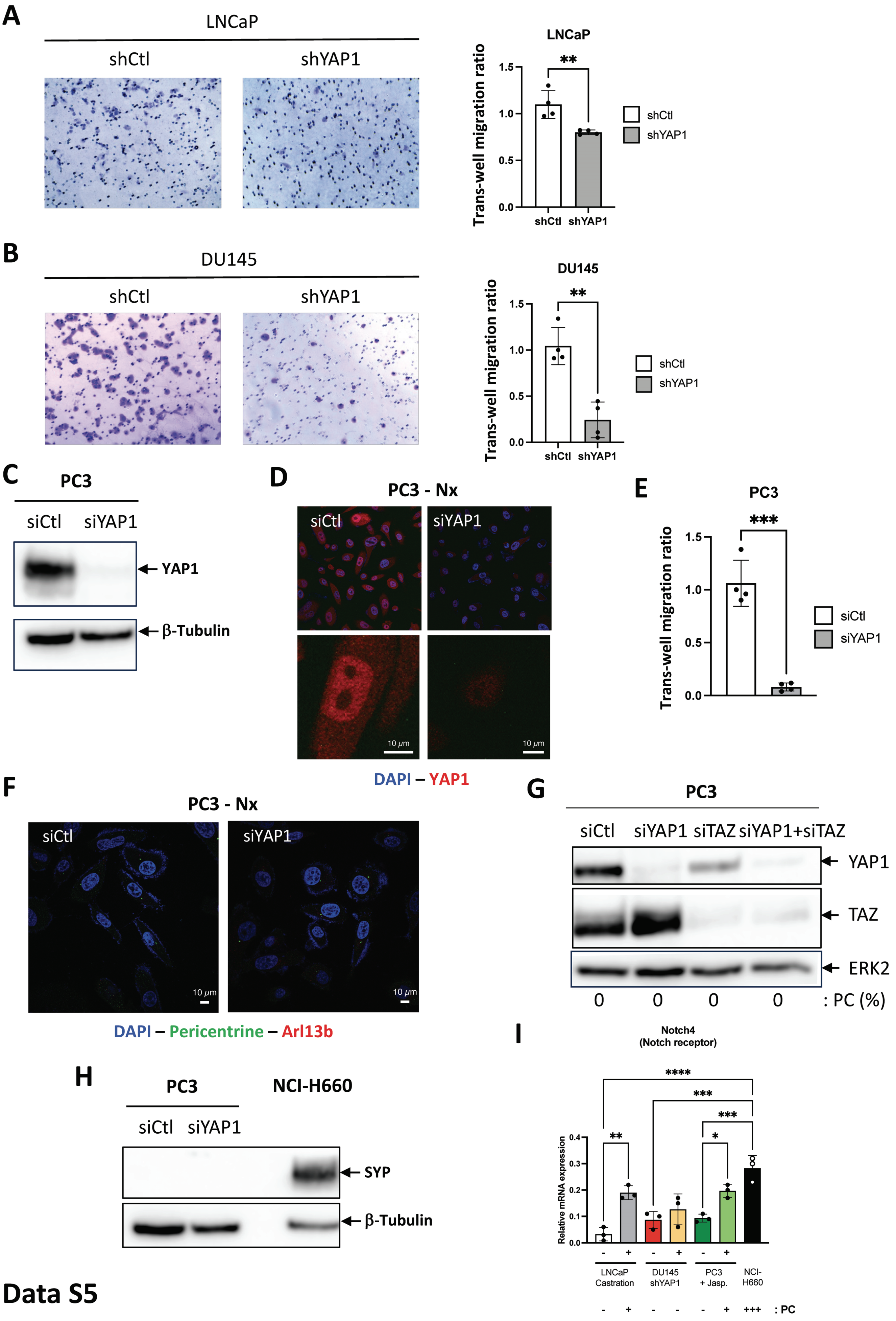
A
LNCaP
shCtl
shYAP1
LNCaP
Trans-well migration ratio
1.5
1.0
0.5
0.0
**
shCtl
shYAP1
B
DU145
shCtl
shYAP1
DU145
**
C
PC3
siCtl
siYAP1
YAP1
β-Tubulin
D
PC3 - Nx
10 μm
DAPI – YAP1
E
PC3
***
siCtl
siYAP1
F
PC3 - Nx
DAPI – Pericentrine – Arl13b
G
PC3
siCtl
siYAP1
siTAZ
siYAP1+siTAZ
YAP1
TAZ
ERK2
0 0 0 0 : PC (%)
H
PC3
NCI-H660
SYP
β-Tubulin
I
Notch4
(Notch receptor)
Relative mRNA expression
****
***
***
**
*
LNCaP Castration
DU145 shYAP1
PC3 + Jasp.
NCI-H660
- + - - - + +++ : PC

Data S5

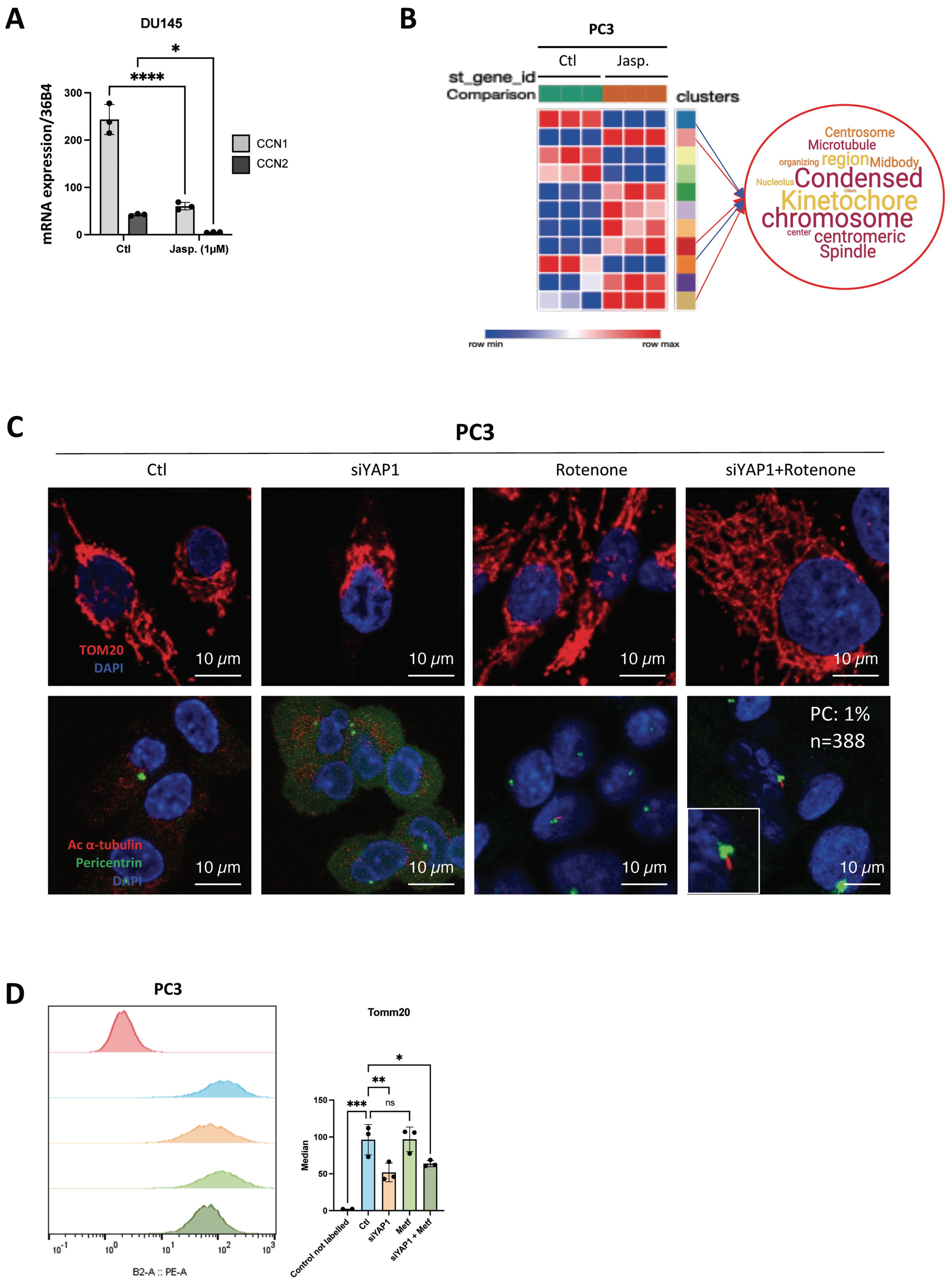
A
DU145
mRNA expression/36B4
300
200
100
0
****
*
CCN1
CCN2
Ctl
Jasp. (1µM)
B
PC3
Ctl
Jasp.
st_gene_id
Comparison
clusters
row min
row max
Centrosome
Microtubule
organizing
region
Midbody
Nucleolus
Condensed
Kinetochore
chromosome
center
centromeric
Spindle
C
PC3
Ctl
siYAP1
Rotenone
siYAP1+Rotenone
TOM20
DAPI
10 µm
Ac α-tubulin
Pericentrin
DAPI
PC: 1%
n=388
D
PC3
B2-A :: PE-A
Tomm20
Median
150
100
50
0
***
**
ns
*
Control not labelled
Ctl
siYAP1
Metf
siYAP1 + Metf

Data S6

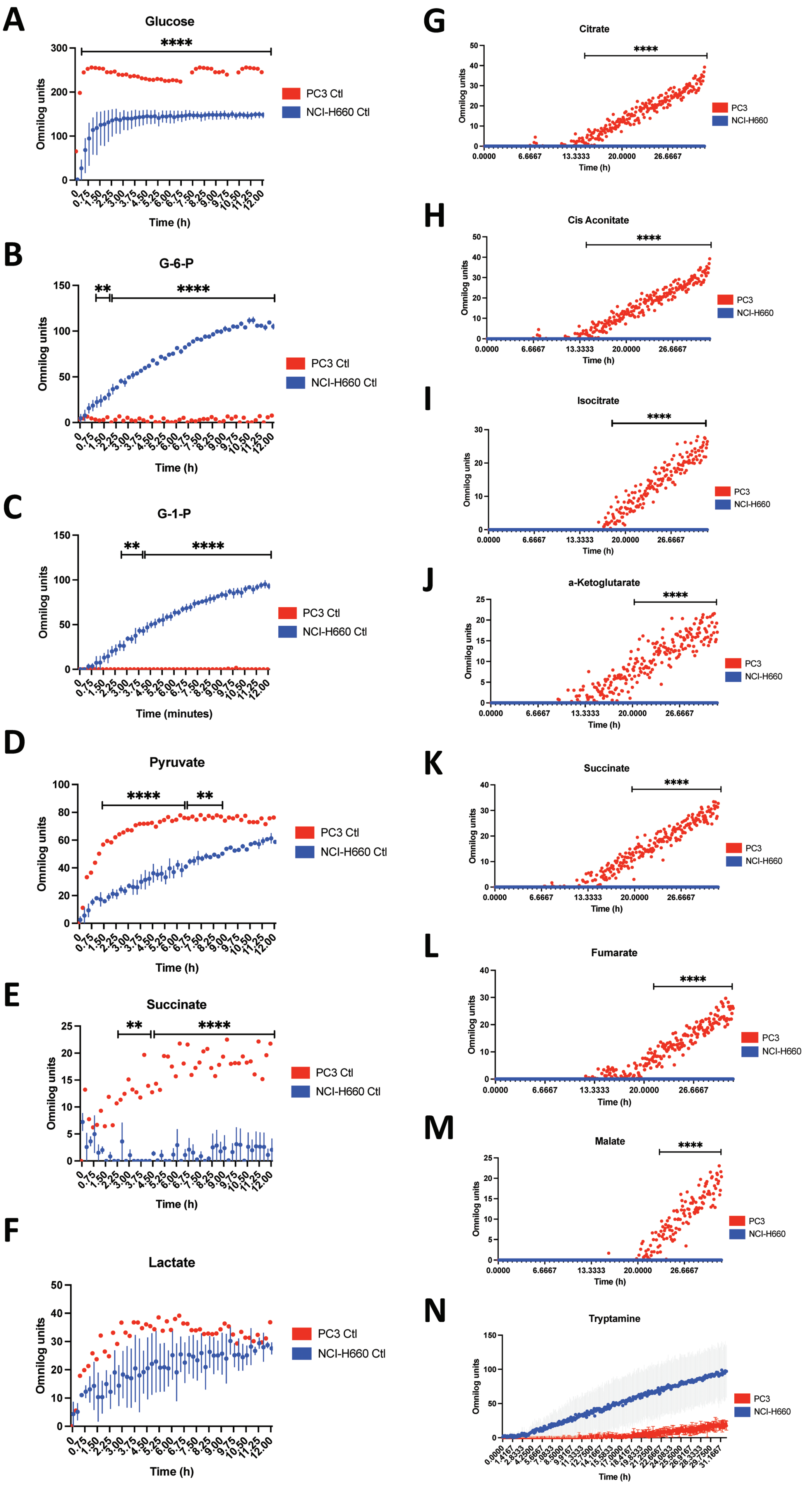

A
Glucose
****
Omnilog units
Time (h)
PC3 Ctl
NCI-H660 Ctl
B
G-6-P
**
****
Omnilog units
Time (h)
PC3 Ctl
NCI-H660 Ctl
C
G-1-P
**
****
Omnilog units
Time (minutes)
PC3 Ctl
NCI-H660 Ctl
D
Pyruvate
****
**
Omnilog units
Time (h)
PC3 Ctl
NCI-H660 Ctl
E
Succinate
**
****
Omnilog units
Time (h)
PC3 Ctl
NCI-H660 Ctl
F
Lactate
Omnilog units
Time (h)
PC3 Ctl
NCI-H660 Ctl
G
Citrate
****
Omnilog units
Time (h)
PC3
NCI-H660
H
Cis Aconitate
****
Omnilog units
Time (h)
PC3
NCI-H660
I
Isocitrate
****
Omnilog units
Time (h)
PC3
NCI-H660
J
a-Ketoglutarate
****
Omnilog units
Time (h)
PC3
NCI-H660
K
Succinate
****
Omnilog units
Time (h)
PC3
NCI-H660
L
Fumarate
****
Omnilog units
Time (h)
PC3
NCI-H660
M
Malate
****
Omnilog units
Time (h)
PC3
NCI-H660
N
Tryptamine
Omnilog units
Time (h)
PC3
NCI-H660

Data S7

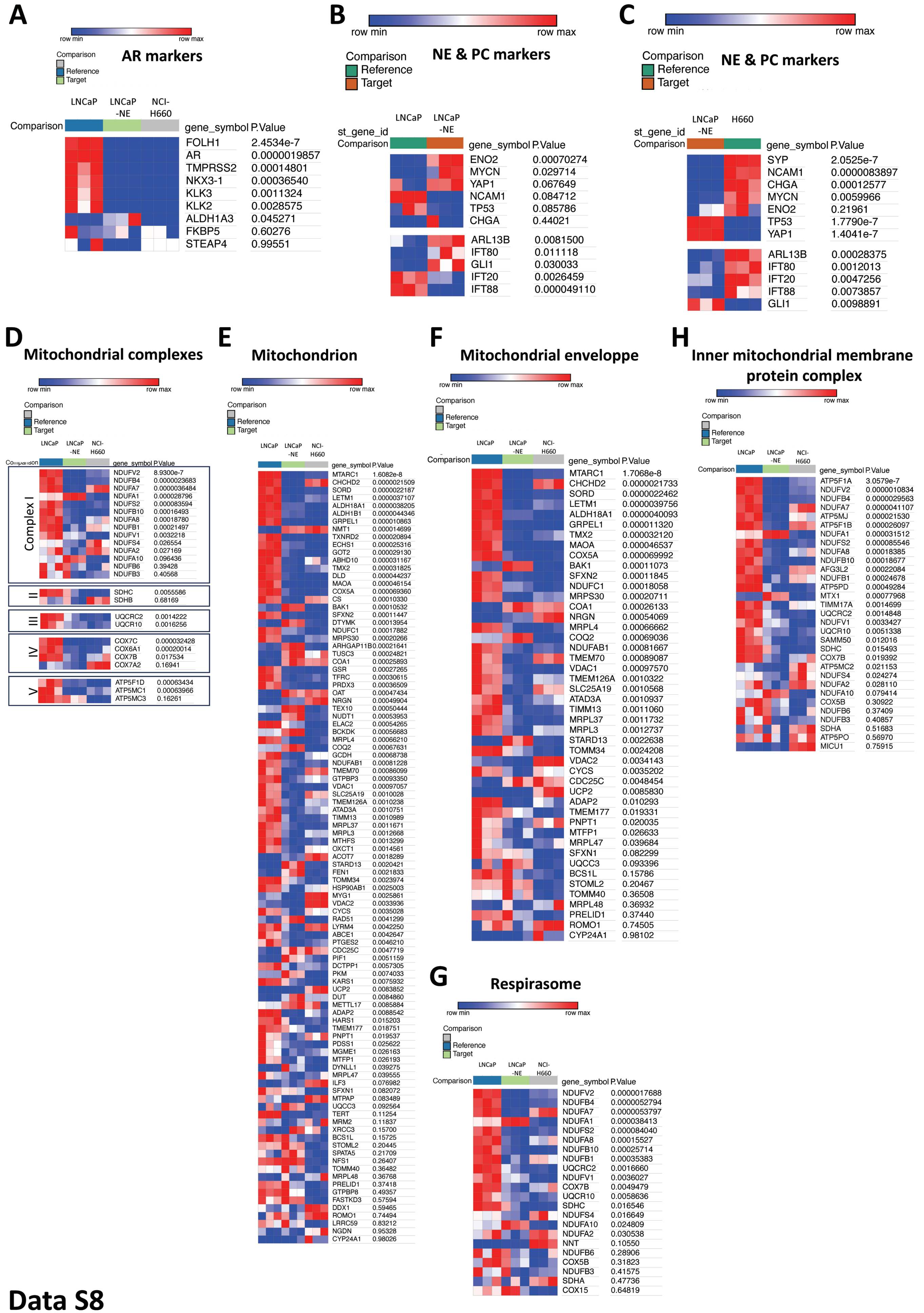
A
row min
row max
Comparison
Reference
Target
AR markers
LNCaP
LNCaP -NE
NCI-H660
Comparison
gene_symbol P.Value
FOLH1 2.4534e-7
AR 0.0000019857
TMPRSS2 0.00014801
NKX3-1 0.00036540
KLK3 0.0011324
KLK2 0.0028575
ALDH1A3 0.045271
FKBP5 0.60276
STEAP4 0.99551
B
row min
row max
Comparison
Reference
Target
NE & PC markers
LNCaP
LNCaP -NE
st_gene_id
Comparison
gene_symbol P.Value
ENO2 0.00070274
MYCN 0.029714
YAP1 0.067649
NCAM1 0.084712
TP53 0.085786
CHGA 0.44021
ARL13B 0.0081500
IFT80 0.011118
GLI1 0.030033
IFT20 0.0026459
IFT88 0.000049110
C
row min
row max
Comparison
Reference
Target
NE & PC markers
LNCaP -NE
H660
st_gene_id
Comparison
gene_symbol P.Value
SYP 2.0525e-7
NCAM1 0.0000083897
CHGA 0.00012577
MYCN 0.0059966
ENO2 0.21961
TP53 1.7790e-7
YAP1 1.4041e-7
ARL13B 0.00028375
IFT80 0.0012013
IFT20 0.0047256
IFT88 0.0073857
GLI1 0.0098891
D
Mitochondrial complexes
row min
row max
Comparison
Reference
Target
LNCaP
LNCaP -NE
NCI-H660
Comparison
gene_symbol P.Value
Complex I
NDUFV2 8.9300e-7
NDUFB4 0.0000023683
NDUFA7 0.0000036484
NDUFA1 0.000028796
NDUFS2 0.000083594
NDUFB10 0.00016493
NDUFA8 0.00018780
NDUFB1 0.00021497
NDUFV1 0.0032218
NDUFS4 0.026554
NDUFA2 0.027169
NDUFA10 0.096436
NDUFB6 0.39428
NDUFB3 0.40568
II
SDHC 0.0055586
SDHB 0.68169
III
UQCRC2 0.0014222
UQCR10 0.0016256
IV
COX7C 0.000032428
COX6A1 0.00020014
COX7B 0.017534
COX7A2 0.16941
V
ATP5F1D 0.00063434
ATP5MC1 0.00063966
ATP5MC3 0.16261
E
Mitochondrion
row min
row max
Comparison
Reference
Target
LNCaP
LNCaP -NE
NCI-H660
gene_symbol P.Value
MTARC1 1.6082e-8
CHCHD2 0.0000021509
SORD 0.0000022187
LETM1 0.0000037107
ALDH18A1 0.0000038205
ALDH1B1 0.0000044346
GRPEL1 0.000010863
NMT1 0.000014699
TXNRD2 0.000020894
ECHS1 0.000025316
GOT2 0.000029130
ABHD10 0.000031167
TMX2 0.000031825
DLD 0.000044237
MAOA 0.000046154
COX5A 0.000069360
CS 0.00010330
BAK1 0.00010532
SFXN2 0.00011447
DTYMK 0.00013954
NDUFC1 0.00017882
MRPS30 0.00020266
ARHGAP11B 0.00021641
TUSC3 0.00024821
COA1 0.00025893
GSR 0.00027265
TFRC 0.00030615
PRDX3 0.00036509
OAT 0.00047434
NRGN 0.00049904
TEX10 0.00050444
NUDT1 0.00053953
ELAC2 0.00054265
BCKDK 0.00056683
MRPL4 0.00066210
COQ2 0.00067631
GCDH 0.00068738
NDUFAB1 0.00081228
TMEM70 0.00086099
GTPBP3 0.00093350
VDAC1 0.00097057
SLC25A19 0.0010028
TMEM126A 0.0010238
ATAD3A 0.0010751
TIMM13 0.0010989
MRPL37 0.0011671
MRPL3 0.0012668
MTHFS 0.0013299
OXCT1 0.0014561
ACOT7 0.0018289
STARD13 0.0020421
FEN1 0.0021833
TOMM34 0.0023974
HSP90AB1 0.0025003
MYG1 0.0025861
VDAC2 0.0033936
CYCS 0.0035028
RAD51 0.0041299
LYRM4 0.0042250
ABCE1 0.0042647
PTGES2 0.0046210
CDC25C 0.0047719
PIF1 0.0051159
DCTPP1 0.0057305
PKM 0.0074033
KARS1 0.0075932
UCP2 0.0083852
DUT 0.0084860
METTL17 0.0085884
ADAP2 0.0088542
HARS1 0.015203
TMEM177 0.018751
PNPT1 0.019537
PDSS1 0.025622
MGME1 0.026163
MTFP1 0.026193
DYNLL1 0.039275
MRPL47 0.039555
ILF3 0.076982
SFXN1 0.082072
MTPAP 0.083489
UQCC3 0.092564
TERT 0.11254
MRM2 0.11837
XRCC3 0.15700
BCS1L 0.15725
STOML2 0.20445
SPATA5 0.21709
NFS1 0.26407
TOMM40 0.36482
MRPL48 0.36768
PRELID1 0.37418
GTPBP8 0.49357
FASTKD3 0.57594
DDX1 0.59465
ROMO1 0.74494
LRRC59 0.83212
NGDN 0.95328
CYP24A1 0.98026
F
Mitochondrial enveloppe
row min
row max
Comparison
Reference
Target
LNCaP
LNCaP -NE
NCI-H660
Comparison
gene_symbol P.Value
MTARC1 1.7068e-8
CHCHD2 0.0000021733
SORD 0.0000022462
LETM1 0.0000039756
ALDH18A1 0.0000040093
GRPEL1 0.000011320
TMX2 0.000032120
MAOA 0.000046537
COX5A 0.000069992
BAK1 0.00011073
SFXN2 0.00011845
NDUFC1 0.00018058
MRPS30 0.00020711
COA1 0.00026133
NRGN 0.00054069
MRPL4 0.00066662
COQ2 0.00069036
NDUFAB1 0.00081667
TMEM70 0.00089087
VDAC1 0.00097570
TMEM126A 0.0010322
SLC25A19 0.0010568
ATAD3A 0.0010937
TIMM13 0.0011060
MRPL37 0.0011732
MRPL3 0.0012737
STARD13 0.0022638
TOMM34 0.0024208
VDAC2 0.0034143
CYCS 0.0035202
CDC25C 0.0048454
UCP2 0.0085830
ADAP2 0.010293
TMEM177 0.019331
PNPT1 0.020035
MTFP1 0.026633
MRPL47 0.039684
SFXN1 0.082299
UQCC3 0.093396
BCS1L 0.15786
STOML2 0.20467
TOMM40 0.36508
MRPL48 0.36932
PRELID1 0.37440
ROMO1 0.74505
CYP24A1 0.98102
G
Respirasome
row min
row max
Comparison
Reference
Target
LNCaP
LNCaP -NE
NCI-H660
Comparison
gene_symbol P.Value
NDUFV2 0.0000017688
NDUFB4 0.0000052794
NDUFA7 0.0000053797
NDUFA1 0.000038413
NDUFS2 0.000084040
NDUFA8 0.00015527
NDUFB10 0.00025714
NDUFB1 0.00035383
UQCRC2 0.0016660
NDUFV1 0.0036027
COX7B 0.0049479
UQCR10 0.0058636
SDHC 0.016546
NDUFS4 0.016649
NDUFA10 0.024809
NDUFA2 0.030538
NNT 0.10550
NDUFB6 0.28906
COX5B 0.31823
NDUFB3 0.41575
SDHA 0.47736
COX15 0.64819
H
Inner mitochondrial membrane protein complex
row min
row max
Comparison
Reference
Target
LNCaP
LNCaP -NE
NCI-H660
Comparison
gene_symbol P.Value
ATP5F1A 3.0579e-7
NDUFV2 0.0000010834
NDUFB4 0.0000029563
NDUFA7 0.0000041107
ATP5MJ 0.000021530
ATP5F1B 0.000026097
NDUFA1 0.000031512
NDUFS2 0.000085546
NDUFA8 0.00018385
NDUFB10 0.00018677
AFG3L2 0.00022084
NDUFB1 0.00024678
ATP5PD 0.00049284
MTX1 0.00077968
TIMM17A 0.0014699
UQCRC2 0.0014848
NDUFV1 0.0033427
UQCR10 0.0051338
SAMM50 0.012016
SDHC 0.015493
COX7B 0.019392
ATP5MC2 0.021153
NDUFS4 0.024274
NDUFA2 0.028110
NDUFA10 0.079414
COX5B 0.30922
NDUFB6 0.37409
NDUFB3 0.40857
SDHA 0.51683
ATP5PO 0.56970
MICU1 0.75915

Data S8

A

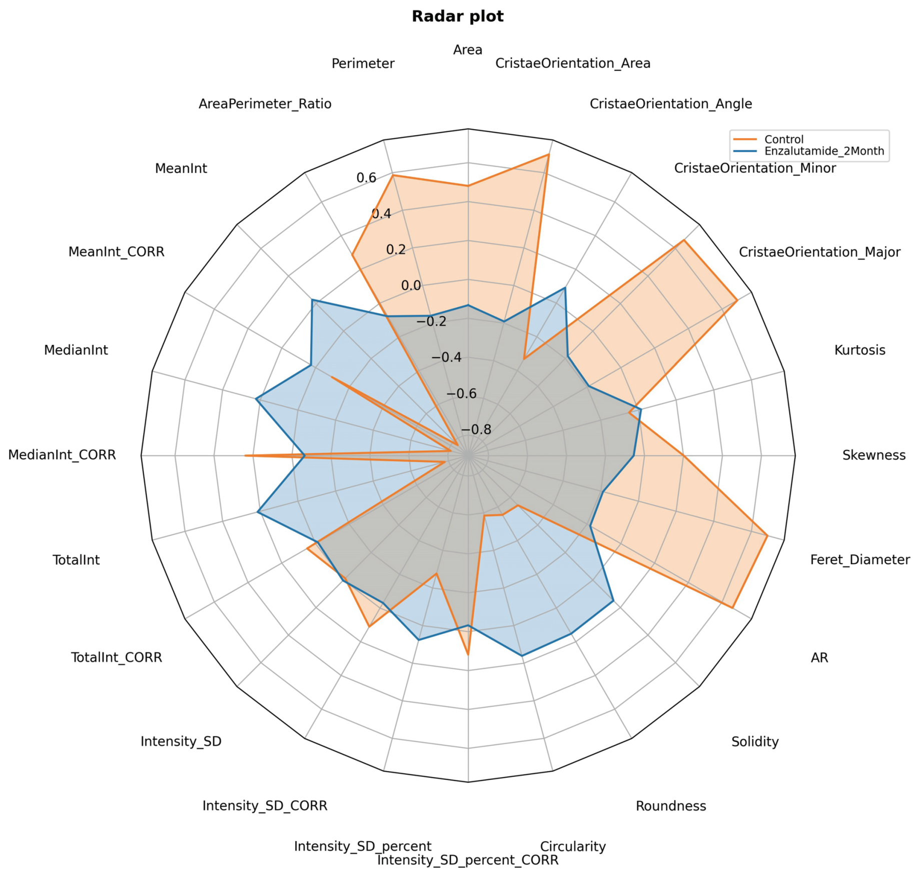

Radar plot
Area
CristaeOrientation_Area
CristaeOrientation_Angle
CristaeOrientation_Minor
CristaeOrientation_Major
Kurtosis
Skewness
Feret_Diameter
AR
Solidity
Roundness
Circularity
Intensity_SD_percent_CORR
Intensity_SD_percent
Intensity_SD_CORR
Intensity_SD
TotalInt_CORR
TotalInt
MedianInt_CORR
MedianInt
MeanInt_CORR
MeanInt
AreaPerimeter_Ratio
Perimeter
Control
Enzalutamide_2Month
0.6
0.4
0.2
0.0
−0.2
−0.4
−0.6
−0.8

B

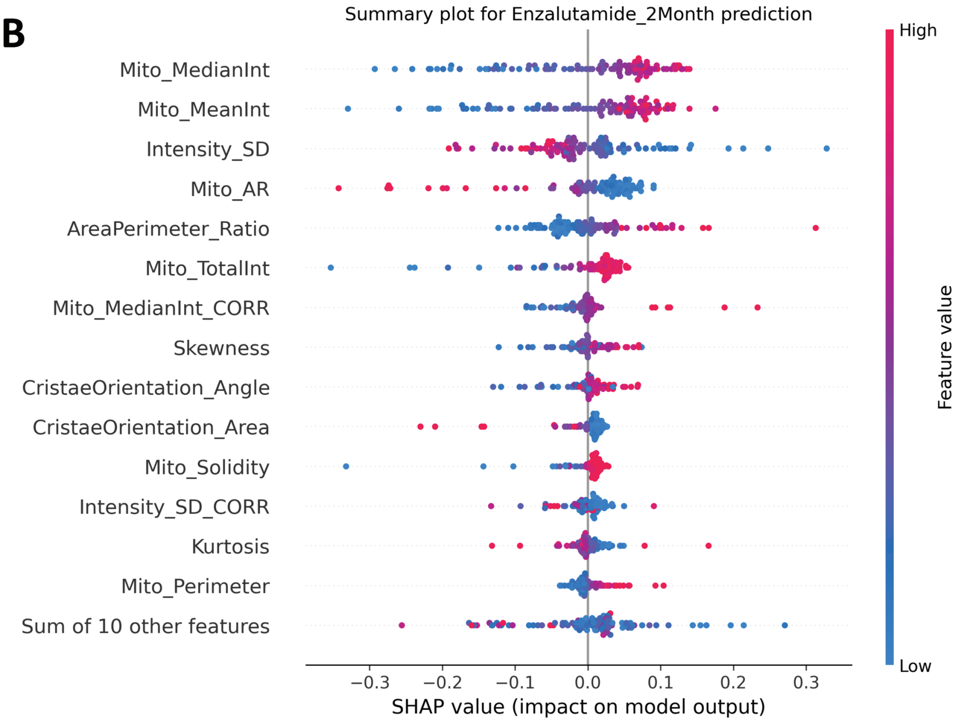

Summary plot for Enzalutamide_2Month prediction
Mito_MedianInt
Mito_MeanInt
Intensity_SD
Mito_AR
AreaPerimeter_Ratio
Mito_TotalInt
Mito_MedianInt_CORR
Skewness
CristaeOrientation_Angle
CristaeOrientation_Area
Mito_Solidity
Intensity_SD_CORR
Kurtosis
Mito_Perimeter
Sum of 10 other features
−0.3 −0.2 −0.1 0.0 0.1 0.2 0.3
SHAP value (impact on model output)
High
Feature value
Low

Data S9

A

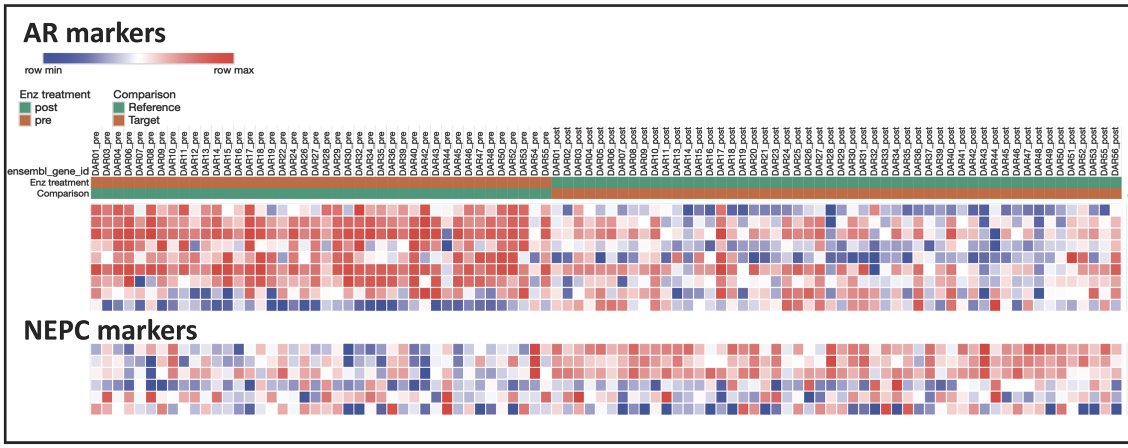

gene_id	t	P.Value	B
TMPRSS2	-9.8229	4.2922e-16	26.153
KLK3	-8.1195	1.7720e-12	17.948
KLK2	-7.1484	1.8542e-10	13.377
ALDH1A3	-6.9219	5.3761e-10	12.333
FKBP5	-6.6944	1.5501e-9	11.295
NKX3-1	-5.9248	5.0905e-8	7.8825
STEAP4	-5.9059	5.5350e-8	7.8009
FOLH1	-0.19211	0.84807	-7.0492
AR	7.7546	1.0312e-11	16.216

YAP1	7.7472	1.0685e-11	16.181
NCAM1	6.0264	3.2401e-8	8.3231
ENO2	5.4328	4.3280e-7	5.8001
CHGA	2.0565	0.042496	-4.9924
TP53	-1.2719	0.20655	-6.2630
MYCN	-1.1254	0.26330	-6.4366

B

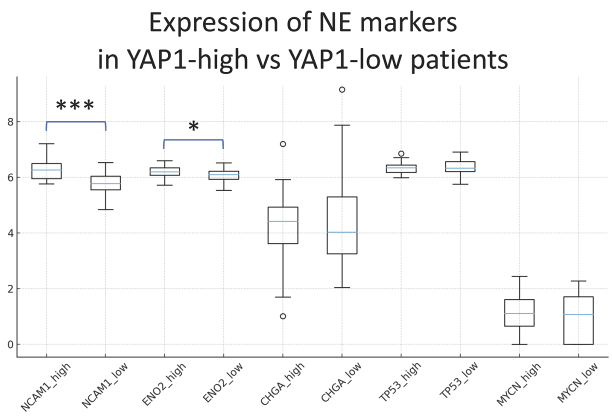

C

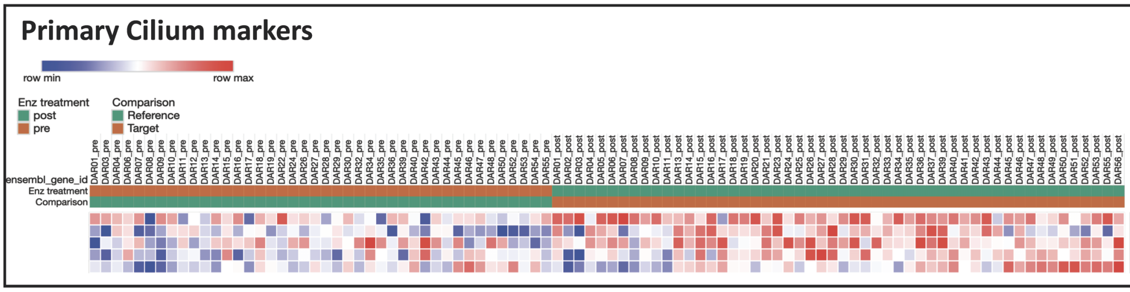

gene_id	t	P.Value	B
GLI1	5.8273	7.8299e-8	7.4629
IFT80	5.1465	0.0000014460	4.6308
IFT20	3.4739	0.00077695	-1.3733
IFT88	3.4579	0.00081887	-1.4224
ARL13B	2.1707	0.032471	-4.7614

D

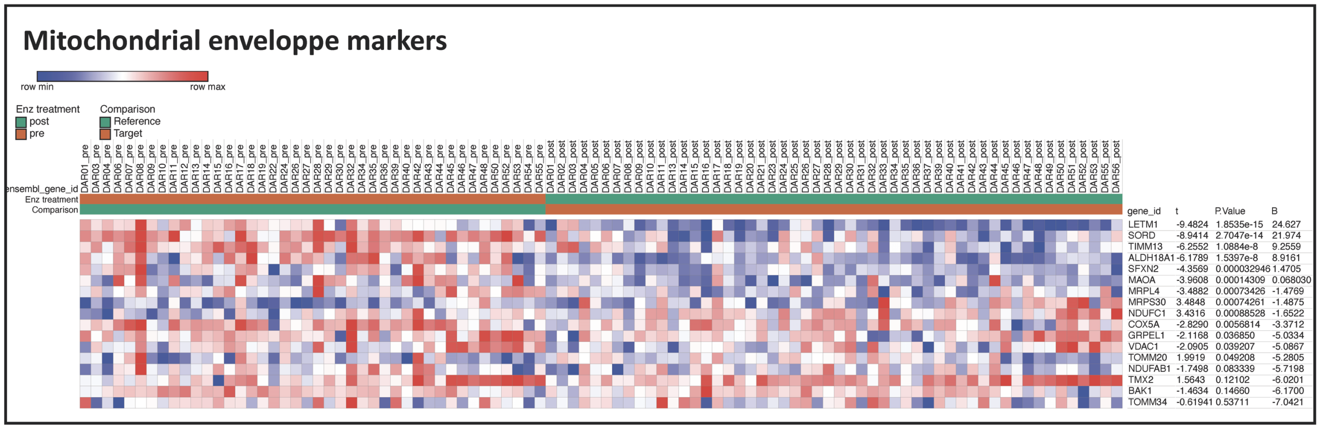

gene_id	t	P.Value	B
LETM1	-9.4824	1.8535e-15	24.627
SORD	-8.9414	2.7047e-14	21.974
TIMM13	-6.2552	1.0884e-8	9.2559
ALDH18A1	-6.1789	1.5397e-8	8.9161
SFXN2	-4.3569	0.000032946	1.4705
MAOA	-3.9608	0.00014309	0.068030
MRPL4	-3.4882	0.00073426	-1.4769
MRPS30	3.4848	0.00074261	-1.4875
NDUFC1	3.4316	0.00088528	-1.6522
COX5A	-2.8290	0.0056814	-3.3712
GRPEL1	-2.1168	0.036850	-5.0334
VDAC1	-2.0905	0.039207	-5.0867
TOMM20	1.9919	0.049208	-5.2805
NDUFAB1	-1.7498	0.083339	-5.7198
TMX2	1.5643	0.12102	-6.0201
BAK1	-1.4634	0.14660	-6.1700
TOMM34	-0.61941	0.53711	-7.0421

Data S10

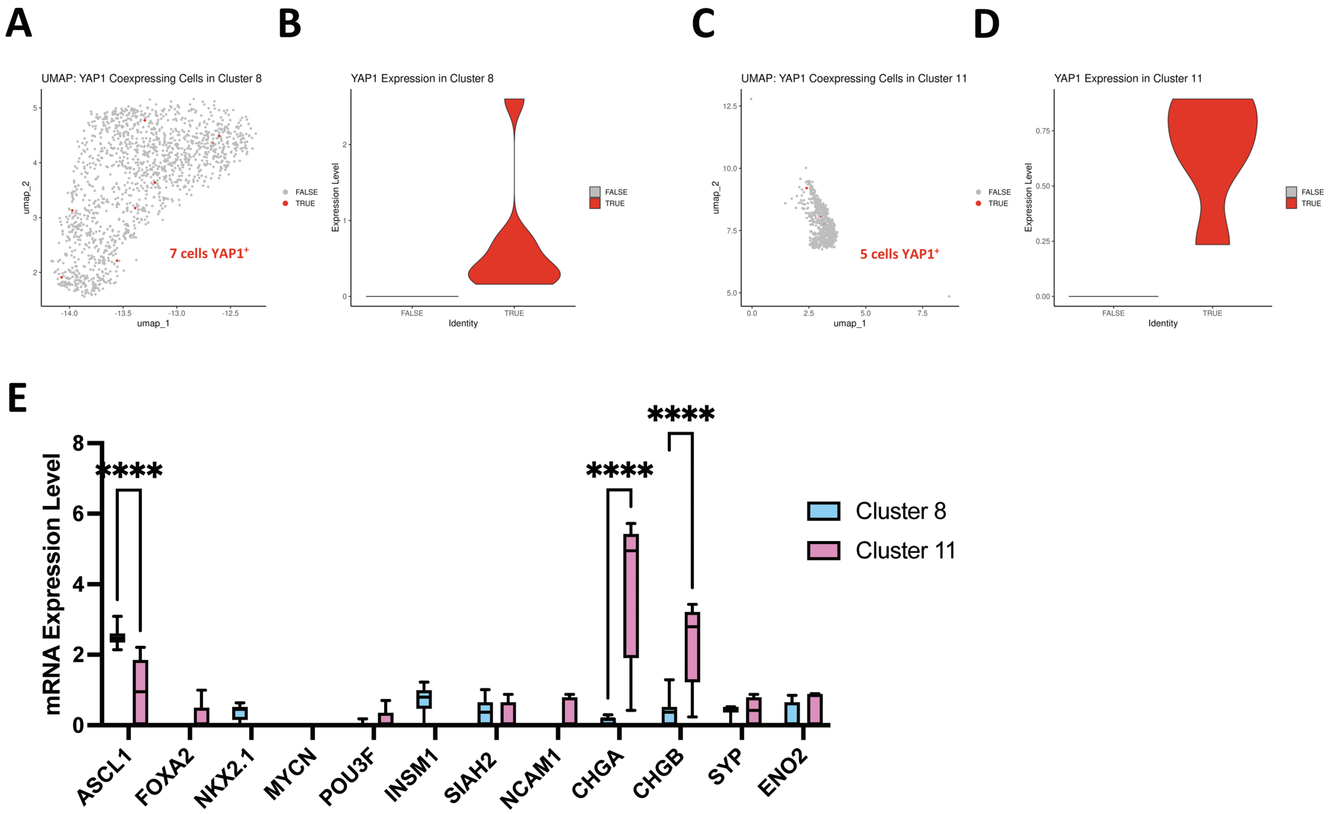

A
B
C
D
UMAP: YAP1 Coexpressing Cells in Cluster 8
7 cells YAP1+
YAP1 Expression in Cluster 8
UMAP: YAP1 Coexpressing Cells in Cluster 11
5 cells YAP1+
YAP1 Expression in Cluster 11
FALSE
TRUE
umap_1
umap_2
Expression Level
Identity
E
mRNA Expression Level
Cluster 8
Cluster 11
****
ASCL1
FOXA2
NKX2.1
MYCN
POU3F
INSM1
SIAH2
NCAM1
CHGA
CHGB
SYP
ENO2

Data S11

A

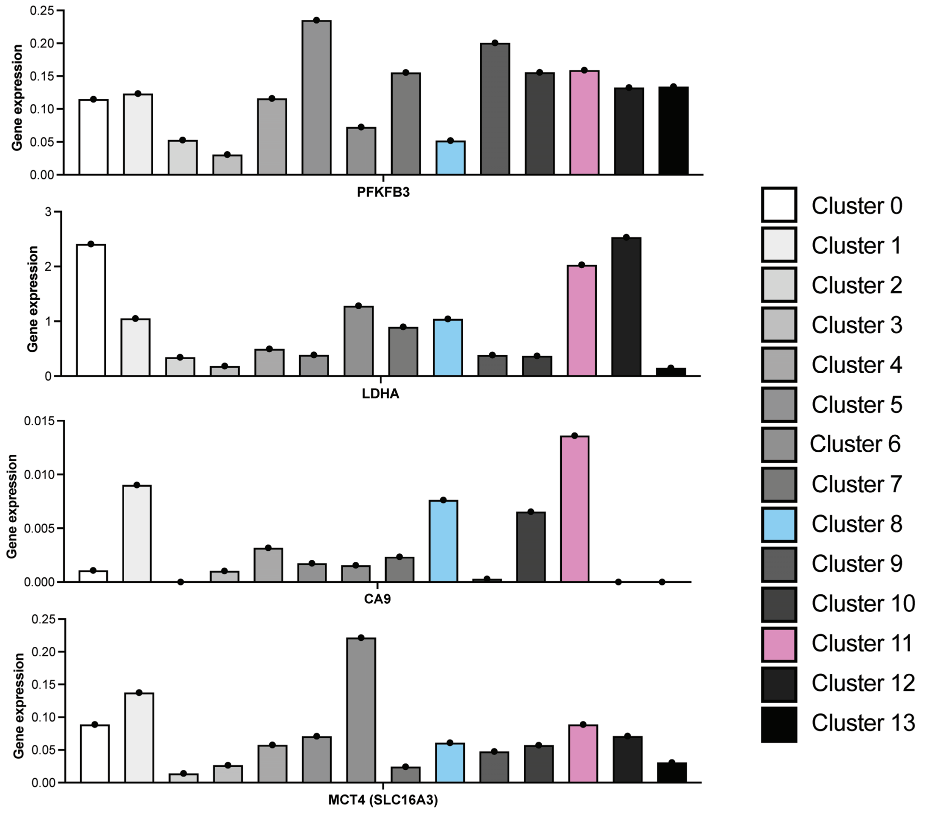

Gene expression
PFKFB3
LDHA
CA9
MCT4 (SLC16A3)
Cluster 0
Cluster 1
Cluster 2
Cluster 3
Cluster 4
Cluster 5
Cluster 6
Cluster 7
Cluster 8
Cluster 9
Cluster 10
Cluster 11
Cluster 12
Cluster 13

Data S12
